# A homoeostatic switch causing glycerol-3-phosphate and phosphoethanolamine accumulation triggers senescence by rewiring lipid metabolism

**DOI:** 10.1038/s42255-023-00972-y

**Published:** 2024-02-19

**Authors:** Khaled Tighanimine, José Américo Nabuco Leva Ferreira Freitas, Ivan Nemazanyy, Alexia Bankolé, Delphine Benarroch-Popivker, Susanne Brodesser, Gregory Doré, Lucas Robinson, Paule Benit, Sophia Ladraa, Yara Bou Saada, Bertrand Friguet, Philippe Bertolino, David Bernard, Guillaume Canaud, Pierre Rustin, Eric Gilson, Oliver Bischof, Stefano Fumagalli, Mario Pende

**Affiliations:** 1 Université Paris Cité, CNRS, Inserm, Institut Necker Enfants Malades (INEM), Paris, France; 2 IMRB, Mondor Institute for Biomedical Research, Inserm U955, Université Paris Est Créteil, UPEC, Faculté de Médecine de Créteil 8, Créteil, France; 3 Sorbonne Université, CNRS, INSERM, Institut de Biologie Paris Seine, Biological Adaptation and Ageing (B2A-IBPS), Paris, France; 4 Platform for Metabolic Analyses, Structure Fédérative de Recherche Necker, INSERM US24/CNRS UAR 3633, Paris, France; 5 Université Côte d’Azur, Inserm, CNRS, Institut for Research on Cancer and Aging (IRCAN), Nice, France; 6 University of Cologne, Faculty of Medicine and University Hospital of Cologne, Cluster of Excellence Cellular Stress Responses in Aging-associated Diseases (CECAD), Cologne, Germany; 7 Institut Pasteur, Plasmodium RNA Biology Unit, Paris, France; 8 Institut Pasteur, Department of Cell Biology and Infection, INSERM, Paris, France; 9 Université Paris Cité, Inserm U1141, NeuroDiderot, Paris, France; 10 Equipe Labellisée la Ligue Contre le Cancer, Centre de Recherche en Cancérologie de Lyon, Inserm U1052, CNRS UMR 5286, Centre Léon Bérard, Université de Lyon, Lyon, France; 11 Unité de médecine translationnelle et thérapies ciblées, Hôpital Necker-Enfants Malades, AP-HP, Paris, France; 12 Department of Medical Genetics, University-Hospital (CHU) of Nice, Nice, France

**Keywords:** Metabolomics, Senescence, Metabolism, Fat metabolism, Lipids

## Abstract

Cellular senescence affects many physiological and pathological processes and is characterized by durable cell cycle arrest, an inflammatory secretory phenotype and metabolic reprogramming. Here, by using dynamic transcriptome and metabolome profiling in human fibroblasts with different subtypes of senescence, we show that a homoeostatic switch that results in glycerol-3-phosphate (G3P) and phosphoethanolamine (pEtN) accumulation links lipid metabolism to the senescence gene expression programme. Mechanistically, p53-dependent glycerol kinase activation and post-translational inactivation of phosphate cytidylyltransferase 2, ethanolamine regulate this metabolic switch, which promotes triglyceride accumulation in lipid droplets and induces the senescence gene expression programme. Conversely, G3P phosphatase and ethanolamine-phosphate phospho-lyase-based scavenging of G3P and pEtN acts in a senomorphic way by reducing G3P and pEtN accumulation. Collectively, our study ties G3P and pEtN accumulation to controlling lipid droplet biogenesis and phospholipid flux in senescent cells, providing a potential therapeutic avenue for targeting senescence and related pathophysiology.

## Abstract (web summary)

Tighanimine et al. perform integrative time-resolved transcriptome and metabolome analysis in senescent cells and find that glycerol-3-phosphate and phosphoethanolamine accumulate and rewire lipid metabolism to promote senescence.

## Main

Senescence is a stable cell cycle arrest in response to diverse forms of non-lethal cellular stress^1,2^. It involves many physiological and pathophysiological processes, including wound healing, embryonic development, age-related diseases and aging. Two potent tumour suppressors, p53 and retinoblastoma (RB), orchestrate establishing and maintaining the senescence phenotype. During senescence, p53 becomes stabilized, inducing the expression of the cyclin-dependent kinase inhibitor CDKN1A (alias p21). RB is instrumental in assembling a co-repressor complex in senescence that inactivates the pro-proliferative transcription factor E2F. RB activity is negatively regulated by CDK-dependent phosphorylation and positively by CDKN2A (alias p16), the expression of which is strongly upregulated in senescence. Unperturbed cell mass accumulation during senescence cell cycle arrest may result in enlarged morphology with a high cytoplasm-to-nucleus ratio^3^. Intracellular compartments are also affected during senescence, with an increase in lysosomal mass and activity reflected in the rise of the senescence biomarker senescence-associated β galactosidase (SABG)^4^.

Senescent cells functionally interact with immune cells via the senescent-associated secretory phenotype (SASP), characterized by the production of inflammatory cytokines (such as interleukin (IL)-6, IL-1α and IL-1β), chemokines (such as CCL2 and CXCL8), immune modulators (prostaglandins) and matrix-remodelling factors (such as MMP, serpin, PAI1 and TIMP)^1^. The SASP may also spread senescence to its cellular environment in a paracrine fashion. Acute responses mostly resolve with the clearing of senescent cells by the immune cells and may represent an important tumour-suppressor mechanism; however, chronic responses maintain an environment promoting tissue inflammation and fibrosis that favours tumour development and other age-related diseases. Therefore, therapeutic strategies aiming to eliminate senescence (senolytic drugs) or offset the SASP (senomorphic drugs) present interesting inroads for treating several age-related pathological conditions^5^.

Cellular metabolism is largely impacted during senescence^6–12^ and the rewiring of these metabolic adaptations may represent a powerful avenue for therapeutic interventions^2^. In response to oncogenic insults and telomere erosion, the mitochondria of senescent cells tend to decrease ATP production and increase reactive oxygen species (ROS)^13^. Hence, energy metabolism is shifted to glycolysis^10^. To regenerate NAD^+^ levels for glycolytic flux and maintain redox status, lactate dehydrogenase (LDH) reduces cytosolic pyruvate to lactate, while malate dehydrogenase 1 (MDH1) reduces oxaloacetate to malate. Another common metabolic hallmark of cellular senescence is the accumulation of lipid droplets^8,12,14^. Profound alterations of triacylglycerol (TAG) metabolism reflect and result from the complex interplay between lipid uptake, synthesis and fatty acid oxidation (FAO)^2^. CD36-mediated free fatty acid (FFA) uptake is upregulated in senescent cells^15^. This effect and increased enzyme activity in fatty acid synthesis may account for TAG accumulation; however, the involvement of fatty acid synthase (FASN) and acetyl-CoA carboxylase (ACC) is variable depending on the cell type and senescence insults, as their activity is rather downregulated during oncogene-induced senescence (OIS)^11,16^. In addition, there is evidence of increased FAO in senescent cells^2^. It is unclear whether FAO upregulation during senescence mainly serves purposes of energy homoeostasis, lipodetoxification, SASP maintenance or epigenetic modifications through histone acetylation^2^.

Similarly, the role of lipid droplet formation may be multi-faceted. It has been proposed as a defence mechanism to cope with metabolic stress. The formation of polyunsaturated fatty acids (PUFAs), enriched in the lipid droplets of senescent cells, could also participate in NAD^+^ recycling^17^. PUFAs are prone to lipid peroxidation in the context of ROS production and may severely harm the integrity of cellular membranes. Thus, lipid droplet formation may represent a protective mechanism to sequester potentially toxic compounds such as glycerolipids and PUFAs away from cell membranes.

On the other hand, lipid accumulation could also contribute to the senescence programme, as the modulation of both CD36-dependent lipid uptake and FASN activity affects the entry into senescence^16^. Furthermore, structural features, such as the stiffness of the extracellular matrix and cell geometry, have been shown to regulate both senescence and lipid droplet formation through changes in the endoplasmic reticulum (ER) and Golgi trafficking^18,19^. In addition, phospholipid (PL) synthesis may be essential to sustain cell growth and organelle remodelling in senescent cells, though this needs to be addressed. Finally, it remains to be established whether strategies altering lipid metabolism and the neutral versus PL switch in senescent cells may act as senolytics and senomorphics or be used as a senogenic anticancer therapy.

In this study, we undertook a time-resolved, multi-layered, integrative profiling approach to identify the clocks driving the senescence programme triggered by distinct senescent stressors. Previously, the combined analysis of transcriptome and epigenome profiles defined the hierarchical organization of the transcription factor network acting on the epigenetic state of enhancers to drive the senescent fate^20^. Here, we used the same experimental setting on human fibroblasts to interrogate the metabolic clock by metabolomic analysis of polar and hydrophobic compounds. Our results identify G3P and pEtN metabolism as potent regulators of the senescent programme at the nexus of TAG and PL metabolism. Furthermore, we show that glycerol kinase (GK) and phosphate cytidylyltransferase 2, ethanolamine (Pcyt2) activities, which catalyse the regulatory steps in TAG and PL synthesis, impact G3P and pEtN levels in a homoeostatic fashion controlling the senescence programme. Finally, we provide evidence that pharmacological inhibitors of GK activity act as senomorphics, thus suggesting a new therapeutic target for interventions in age-related diseases and cancer.

## Results

### The metabolic landscape of senescent cell subtypes

**Fig. 1:**
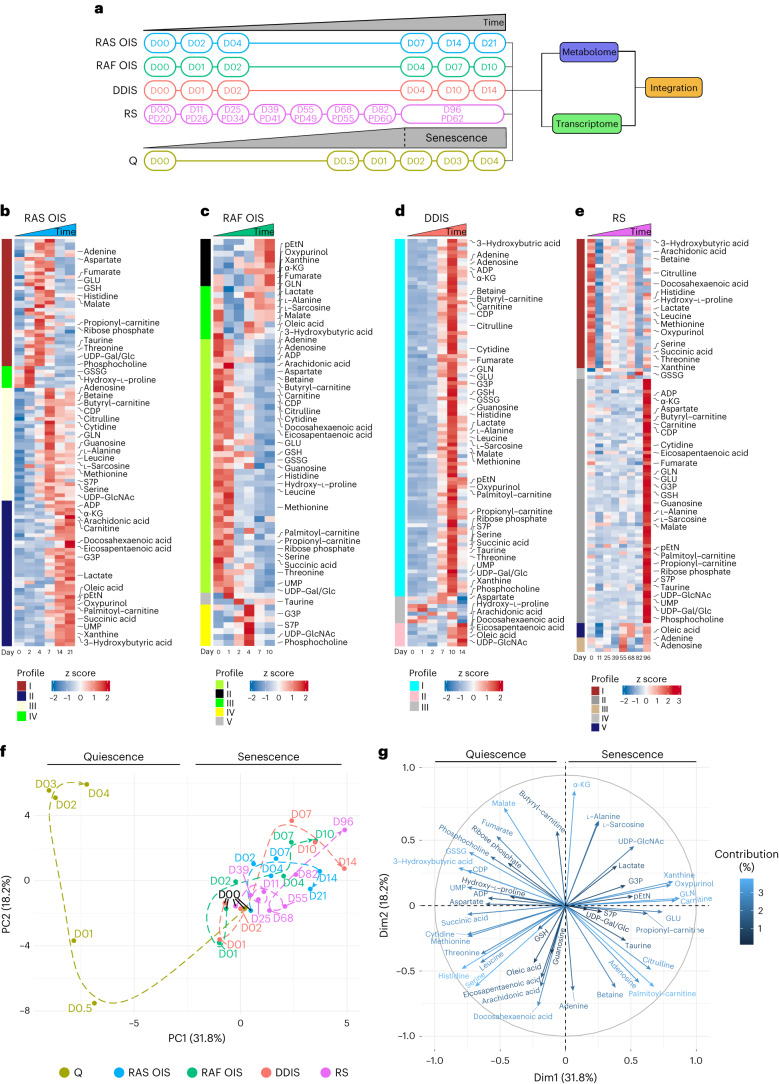
Untargeted metabolomics reveals a shared metabolic signature across several senescence models. **a**, Experimental design for the integrative analysis of time-resolved metabolome and transcriptome datasets obtained from WI38 fibroblasts undergoing RAS and RAF OIS, etoposide-mediated DDIS, RS and quiescence (Q). D, day. **b**–**e**, Heat maps showing modules of temporally coexpressed metabolites in WI38 fibroblasts for the indicated senescence inducers at indicated time points using a hierarchical clustering method (WGCNA). Roman numerals refer to different metabolite modules. Data are expressed as row *z* scores collected from three biologically independent experiments per condition. GSSG, glutathione disulfide (oxidized glutathione); UDP-Gal/Glc, uridine diphosphate galactose/glucose; UDP-GlcNAc, uridine diphosphate *N*-acetylglucosamine. **f**, Integrated dynamic metabolome PCA for cells undergoing RAS and RAF OIS, DDIS, RS and Q as control. Metabolite levels were normalized by the ComBat tool. Dashed lines depict the metabolome trajectory for each treatment. **g**, Correlation circle for the percentage contribution of the indicated metabolites to principal components PC1 and PC2 of **f**.

To identify the metabolic signatures and potential metabolic vulnerabilities of individual senescent cells, we performed targeted, time-resolved metabolic profiling using mass spectrometry (MS)-based analysis in normal, human diploid fibroblasts (strain WI38) exposed to diverse forms of senescence-inducing stress, including hyper-active oncogenes (RAS and RAF OIS), replicative senescence (RS) and DNA damage-induced senescence (DDIS; induced by etoposide). For comparison, we included cells undergoing quiescence by serum withdrawal, as well as proliferating cells (time D00 in each condition) (Fig. 1a). To track senescence dynamics and for downstream metabolome–transcriptome integration, we performed time-series gene expression profiling. As previously published^20^, senescence dynamics are inducer-specific, spanning different time scales. Accordingly, we intermittently sampled cells in line with inducer-specific senescence dynamics to adequately cover the establishment and maintenance phases of senescence (Fig. 1a).

**Extended Data Fig. 1:**
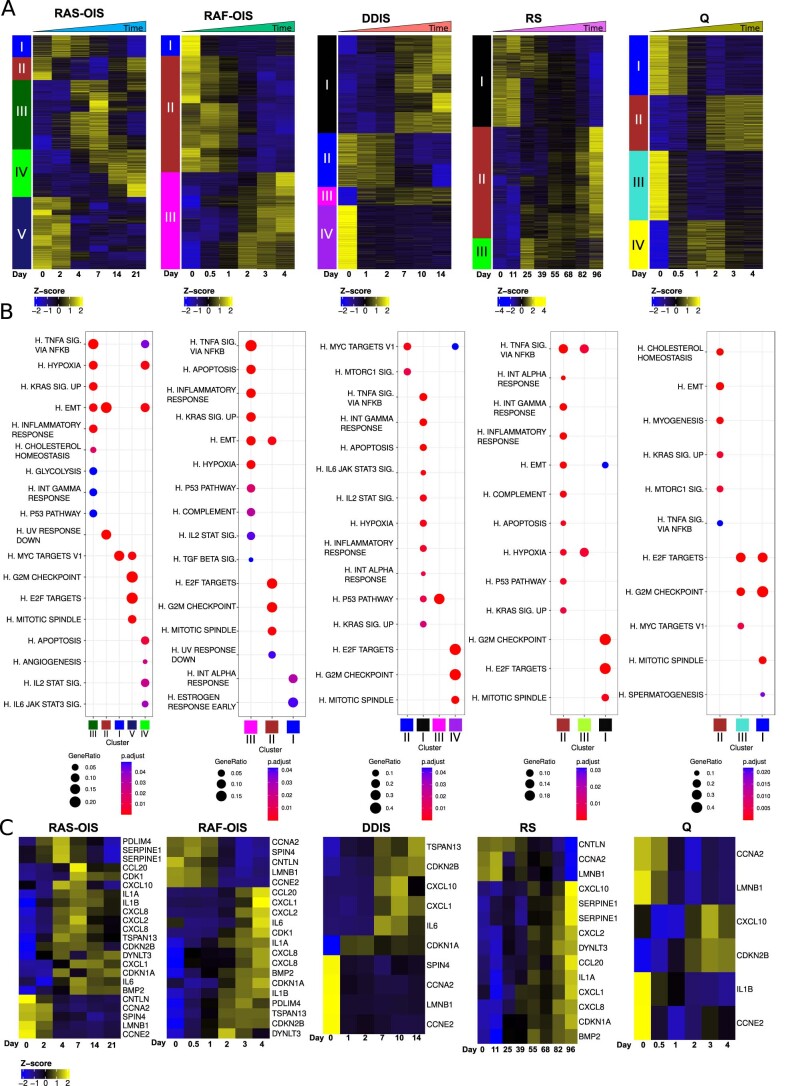
Transcriptome Evolution of Senescence Inducers. A: Heat maps showing modules of temporally coexpressed genes for the indicated senescence inducers and quiescence (Q) at the indicated time points using an unsupervised weighted clustering network analysis (WGCNA) approach. Roman numerals refer to different gene clusters. Data are expressed as row Z scores collected from two biologically independent experiments per condition. B: Functional over-representation map depicting Molecular Signaling Database(MSigDB) hallmark (H.) gene sets associated with each transcriptomic cluster for the indicated senescence inducers. Circles are colour-coded according to the FDR-corrected p value based on the hypergeometric text comparing the overlap between the set of genes in each cluster and the respective list of genes in each MSigDB pathway. Size is proportional to the percentage of genes in the MsigDB gene set belonging to the cluster. N > 100 genes per transcriptomic module for each senescence inducer. Exact values for raw p values, adjusted p values and overlap (absolute and relative) between each pair of sets are reported in Supplementary Table S2. C: Expression heat map of core senescence genes for the indicated senescence inducers and quiescence as control.

Congruent with our published data^20^, differentially expressed genes for each senescent subtype were partitioned into various dynamic gene expression modules (Extended Data Fig. 1a and Supplementary Table 1) with distinct functional over-representation profiles in line with the senescence phenotype (Extended Data Fig. 1b and Supplementary Table 2). In particular, cell-cycle-related (RAS, module V; RAF, module II; DDIS, module IV; and RS, module I) and SASP-related inflammatory transcriptional modules (RAS, modules III and IV; RAF, module III; DDIS, module I; and RS, modules II) were similar across all senescence models, which we further corroborated by expression profiling of a core senescence gene signature (Extended Data Fig. 1c)^21,22^. This analysis confirmed that the cells in our time courses display classic senescence transcriptome features.

**Extended Data Fig. 2:**
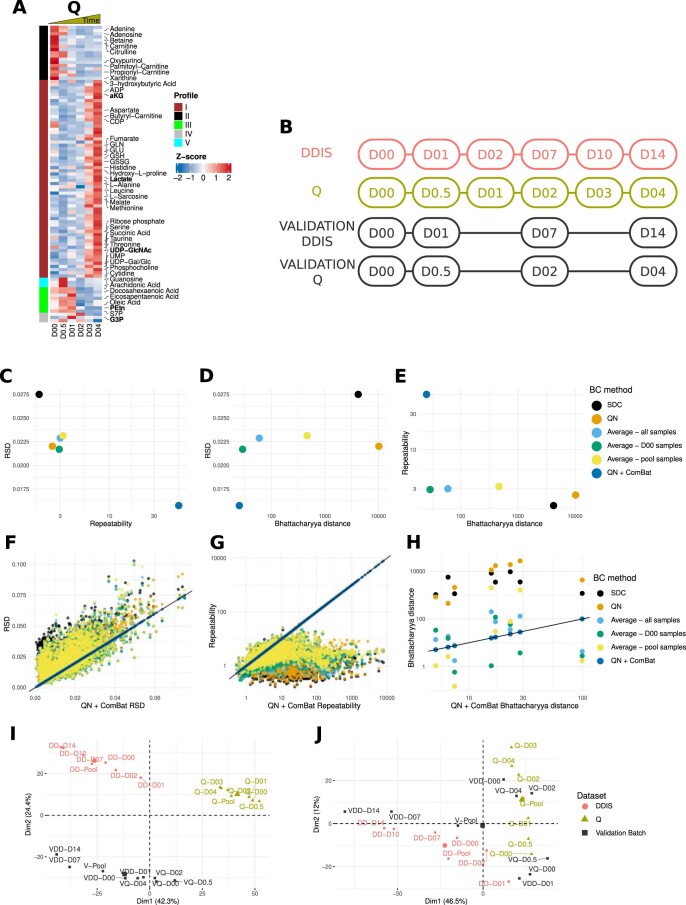
Metabolome profile of quiescent cells and benchmarking batch-effect correction using ComBat. A: Heat map showing modules of temporally coexpressed metabolites in WI38 fibroblasts for quiescence at indicated time points using a hierarchical clustering method (WGCNA). Roman numerals refer to different metabolite clusters. Shown are the top forty metabolites based on the most significant adjusted p values. Data are expressed as row Z scores collected from three biologically independent experiments per condition.B: Experimental design for batch correction validation. DNA damage-induced senescence (DDIS) and quiescence (Q) samples correspond to the same, as shown in Fig. 1a. The validation dataset included technical replicates of a subset of those samples and was measured in the same mass spectrometry run. C-E: Visualization of the average computed values for (C) relative standard deviation (RSD), (D) repeatability, and (E) Bhattacharyya distance for each approach. F-H: Comparison between the obtained values for the ComBat approach following quantile normalization (QN) and the other approaches for each measured peak (or sample) based on three metrics: (F) RSD, (G) repeatability and (H) Bhattacharyya distance. Black lines show the identity function. I- J: PCA plots depicting the average of each sample used for batch correction validation (I) before and (J) after quantile normalization and ComBat.

To characterize the metabolic evolution of the different senescence subtypes, we identified metabolites by a targeted mode in the MS analysis. We independently clustered their time courses using weighted correlation network analysis (WGCNA), identifying metabolite modules with highly correlated metabolite expression trajectories, thereby revealing senescence state- and inducer-specific metabolite patterns (Fig. 1b–e, Extended Data Fig. 2a and Supplementary Tables 3 and 4). The levels of 115 metabolites in RAS OIS, 71 in RAF OIS, 107 in DDIS, 118 in RS and 94 in quiescence were significantly changed (adjusted *P* value < 5%). Consistent with previous studies in various cell biology models of senescence^6–12^, we found increased fatty acid metabolism (such as acylcarnitines), increased glucose shunts to lactate, pentose phosphate pathway (sedoheptulose-7 phosphate; S7P) and uridine diphosphate *N*-acetylglucosamine (UDP-GlcNAc) and altered central carbon metabolism (such as α-ketoglutarate (α-KG) and malate) and the Kennedy pathway (such as pEtN).

Next, we visualized the metabolome dynamics of each senescence inducer to identify commonalities and specificities of individual senescent subtypes, performing an integrated dynamic metabolome principal-component analysis (PCA) (Fig. 1f,g). Because MS-based metabolome analysis is prone to technical variations, making an accurate integration of disparate datasets challenging, we first normalized all metabolome datasets using ComBat (Extended Data Fig. 2b–j)^23^. The PCA illustrated three key points. First, metabolic landscapes of senescence and quiescence are diametrically opposed (PC1, 31.8%). Second, the overall temporal trajectories between senescence subtype metabolomes correspond to neighbouring states, finishing at the top right PCA quadrant (Fig. 1f). Third, plotting the correlation between metabolites and the principal components (Fig. 1g) identifies metabolites that contribute significantly to the quiescence-associated metabolic shifts (top left quadrant of the correlation circle) and senescence-associated metabolic shifts (SAMS) (top right quadrant of the correlation circle), notably α-KG, G3P, pEtN, UDP-GlcNAc, inosine, S7P, oxypurinol, acylcarnitines and lactate.

**Fig. 2:**
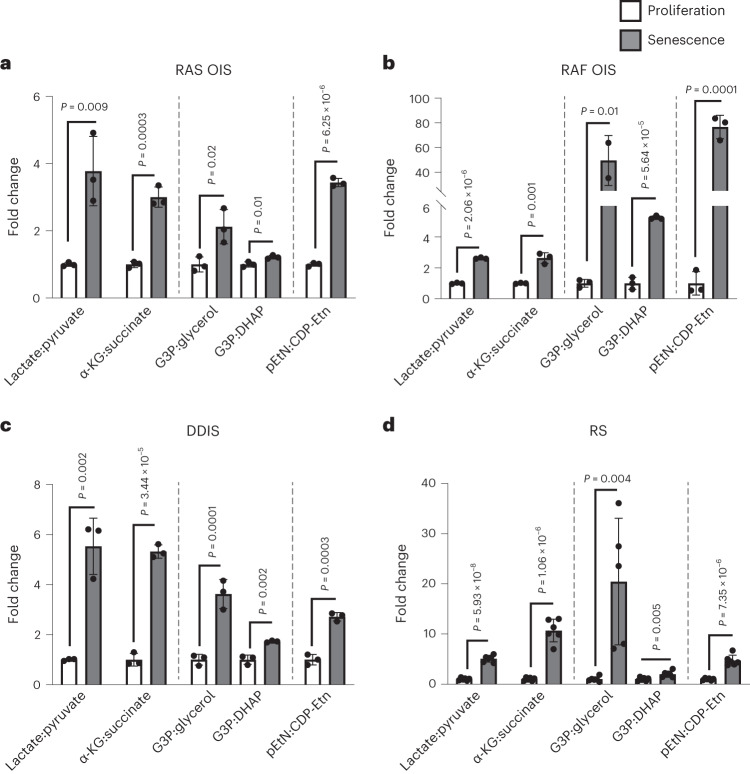
Identification of common SAMS. **a**–**d**, Fold change of the ratios between the indicated metabolites in WI38 fibroblasts undergoing RAS OIS (*n* = 3), RAF OIS (*n* = 3), DDIS (*n* = 3) and RS (*n* = 6) measured for each treatment at the last time point of the kinetics and relative to the values of proliferating cells. The pEtN:CDP-Etn ratio in RS was calculated using D11 as a proliferative control. Bars represent the means of biological replicates ± s.d. Indicated *P* values were calculated using an unpaired two-sided Student’s *t*-test. Source data

**Extended Data Fig. 3:**
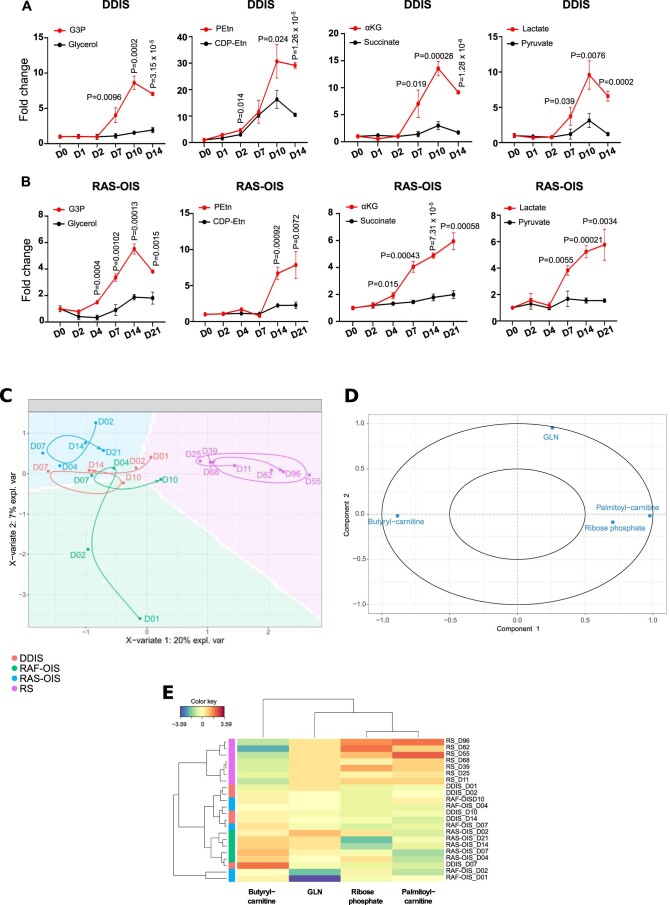
SAMS induction kinetics and specific metabolic response to distinct senescence inducers. Fold changes kinetics of the indicated metabolites belonging to the SAMS in WI38 fibroblasts undergoing DDIS (A) or RAS-OIS (B). n = 3 biologically independent experiments for each time point and condition. Indicated p values were calculated using an unpaired two-sided Student’s t-test. Data are presented as mean values +/- SD. C: Sparse Least Squares Regression – Discriminant Analysis (sPLS-DA) depicting the two orthogonal components that maximize the separation of samples treated with different CS inducers. Metabolite levels were normalized by the Combat tool considering fibroblasts undergoing RAS- and RAF-OIS, DDIS, and RS. Dashed lines depict the metabolome trajectory for each treatment. The background colour depicts the predicted CS inducer given a sample metabolic state. D: Correlation circle depicting the projection of the sPLS-DA selected metabolites in each component. E: Normalized levels for the metabolites which activity best discriminate experimental treatment. Source data

We consolidated the SAMS by calculating, for each senescence subtype, the ratios between metabolites and their immediate precursors or end-products in the same metabolic pathway, using as denominator start (proliferation) and as numerator end point (senescence) metabolite levels of the individual time courses (Fig. 2a–d). Irrespective of the inducer, senescent cells significantly increased lactate:pyruvate, α-KG:succinate, G3P:glycerol, G3P:di-hydroxy acetone phosphate (DHAP) and pEtN:CDP-ethanolamine (Etn) ratios. To underscore the kinetics of these metabolites, we visualized the curve of their fold changes over time compared to day 0 in RAS OIS and DDIS. In both instances, starting 2 days after stress induction, the metabolite shift increased almost linearly before reaching a plateau at 10–14 days of treatment (Extended Data Fig. 3a,b). An increased lactate:pyruvate ratio is consistent with the glycolytic shift and mitochondrial activity decrease observed in senescence^10,13^. In addition, a high proportion of the two oncometabolites, α-KG and succinate, was previously observed as a p53-dependent senescence response of KRAS-mutant cancer cells, leading to the modulation of α-KG-dependent dioxygenases and tumour suppression^23^; however, the mechanistic underpinnings and functional implications of altered G3P:DHAP, G3P:glycerol and pEtN:CDP-Etn ratios in senescence regulation are unknown.

Next, we performed a sparse partial least squares discriminant analysis (sPLS-DA) to identify metabolite signatures that could discriminate between the different senescence inducers (Extended Data Fig. 3c–e). sPLS-DA separated samples according to their treatment in three sectors (Extended Data Fig. 3c), with cells undergoing RS (purple sector), RAS OIS (blue sector) and RAF OIS (green sector) following distinct dynamics. In comparison, DDIS was intercalated between the three sectors. We then associated each sPLS-DA component with its respective metabolite(s) and its/their corresponding median level (Extended Data Fig. 3d,e). We observed that metabolites positively related to the first component (palmitoyl-carnitine and ribose phosphate; Extended Data Fig. 3d) were produced at exceptionally high levels in RS, thus distinguishing it from the other senescence inducers (Extended Data Fig. 3e). Conversely, butyryl-carnitine was negatively associated with the first component (Extended Data Fig. 3d), presenting higher levels, especially in RAS OIS and DDIS (Extended Data Fig. 3e). Finally, GLN was positively associated with the second sPLS-DA component (Extended Data Fig. 3d) and its lower levels specified RAF OIS (Extended Data Fig. 3e).

**Extended Data Fig. 4:**
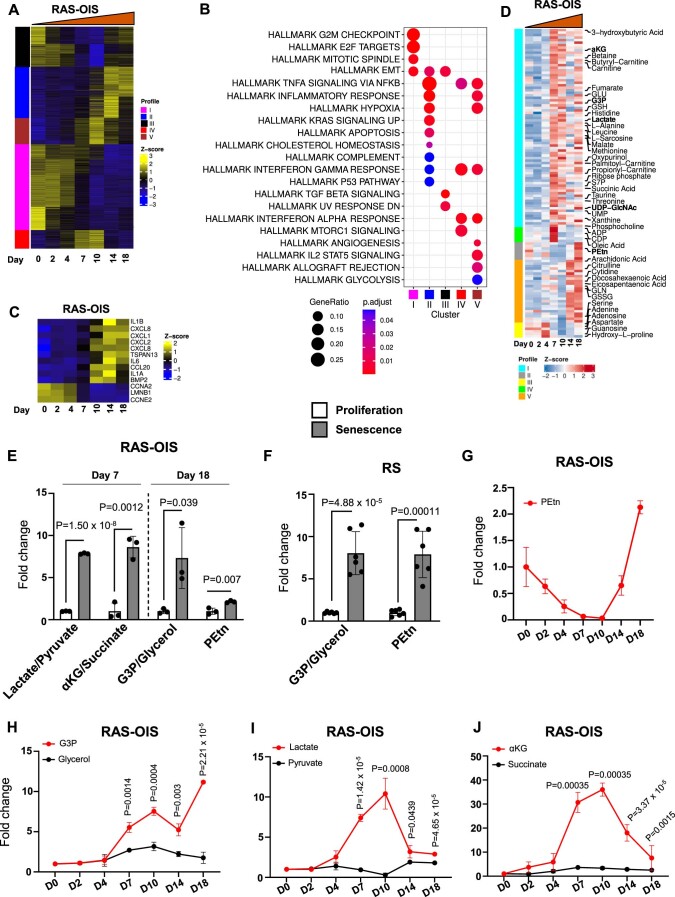
Transcriptome and metabolome profiles of senescent myoblasts. A,D: Heat maps showing modules of temporally coexpressed (A) genes and (D) metabolites for myoblasts undergoing RAS-OIS using an unsupervised weighted clustering network analysis (WGCNA) approach. Roman numerals refer to different coexpression clusters. Data are expressed as row Z scores collected from (A) two and (D) three biologically independent experiments per condition. B: Functional over-representation map depicting Molecular Signaling Database(MSigDB) hallmark (H.) gene sets associated with each transcriptomic cluster for the indicated senescence inducers. Circles are colour-coded according to the FDR-corrected p value based on the hypergeometric text comparing the overlap between the set of genes in each cluster and the respective list of genes in each MSigDB pathway. Size is proportional to the percentage of genes in the MsigDB gene set belonging to the cluster. N > 100 genes per transcriptomic module for each senescence inducer. Exact values for raw p values, adjusted p values and overlap (absolute and relative) between each pair of sets are reported in Supplementary Table S2. C: Expression heat map of core senescence genes for myoblasts undergoing RAS-OIS. E: Fold change of the ratios between the indicated metabolites or of metabolite levels in myoblasts undergoing RAS-OIS (n = 3), measured at the indicated time point of the kinetics and relative to the values of proliferating cells. Data are presented as mean values +/- SD. Indicated p values were calculated using an unpaired two-sided Student’s t-test. F: Fold change of the ratios between the indicated metabolites or of metabolite levels in myoblasts undergoing replicative senescence (n = 6) and relative to the values of proliferating cells. Data are presented as mean values +/- SD. Indicated p values were calculated using an unpaired two-sided Student’s t-test. G-J: Fold changes kinetics of the indicated metabolites belonging to the SAMS in myoblasts undergoing RAS-OIS. n = 3 biologically independent experiments for each time point. Indicated p values were calculated using an unpaired two-sided Student’s t-test. Data are presented as mean values +/- SD. (The panel C contains small letters and numbers that overlap with the heatmap and the X axis. They need to be removed). Source data

We confirmed these results in additional senescence models: human primary myoblasts undergoing RAS OIS and RS (Extended Data Fig. 4a). In particular, cell-cycle- and SASP-related transcriptional modules (Extended Data Fig. 4a,b), senescence index (Extended Data Fig. 4c), SAMS (Extended Data Fig. 4d–f) and its kinetics (Extended Data Fig. 4g–j) were highly similar.

Altogether, our investigation of metabolome dynamics in different cell biology models of senescence defined a dynamic inducer-specific modular organization of the senescence metabolic programme with a shared and pronounced metabolic shift, particularly in central carbon metabolism, toward G3P and pEtN accumulation.

### Inhibition of mTOR and α-KG links SAMS and SASP expression

To test whether SAMS was predictive of senescence status, we administered the mTOR inhibitor rapamycin, an established senomorphic^24–26^, to WI38 cells undergoing DDIS and dimethyloxalylglycine (DMOG), a hypoxia-mimetic and competitive α-KG antagonist^24,27^, to cells undergoing RAS OIS. Hypoxia-mimetic compounds were recently shown to suppress SASP expression in vitro and in vivo^28^.

**Fig. 3:**
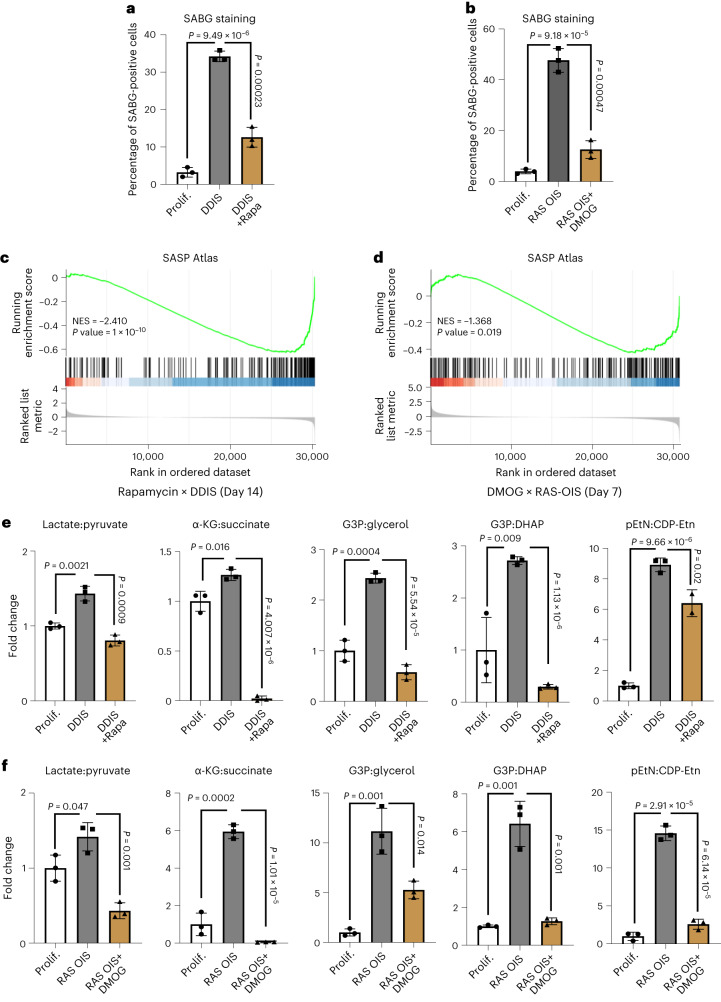
Senescence repression correlates with SAMS repression. **a**, Percentage of SABG-positive cells of cultures of WI38 fibroblasts non-treated (Prolif.) undergoing DDIS in the presence or the absence of rapamycin for 7 days. **b**, Percentage of SABG-positive cells of WI38 fibroblasts non-treated (Prolif.) or undergoing RAS-induced senescence (RAS OIS) treated or not with DMOG for 7 days. **c**,**d**, GSEA enrichment of SASP genes of DDIS rapamycin-treated cells (14 days) (**c**) and RAS OIS DMOG-treated cells (7 days) (**d**). NES, normalized enrichment score. **e**,**f**, Fold changes of SAMS in WI38 fibroblasts undergoing DDIS ± rapamycin for 14 days (**e**) and RAS OIS ± DMOG for 7 days (**f**). For **a**,**b**,**e**,**f**, bars represent the means of three biological replicates ± s.d. Indicated *P* values were calculated using an unpaired two-sided Student’s *t*-test. Source data

**Extended Data Fig. 5:**
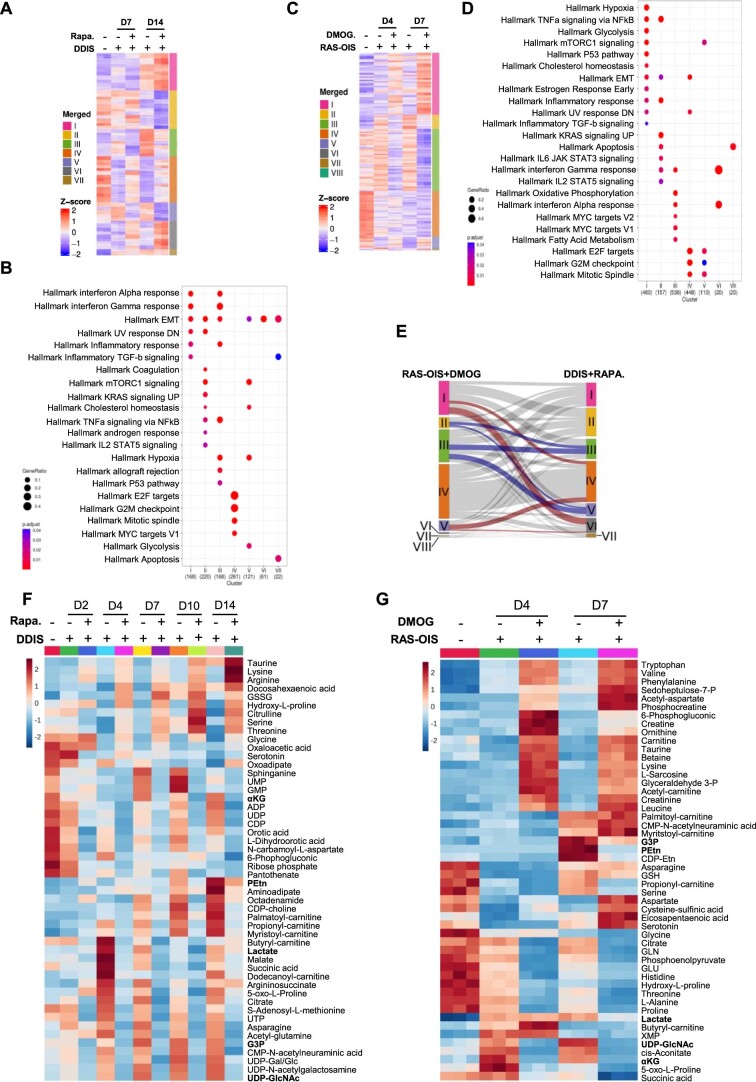
Rapamycin and DMOG revert transcriptomic features of senescence. A: Heat map showing modules of temporally coexpressed genes for WI38 cells undergoing etoposide-mediated DDIS in the presence (+) or the absence (-) of rapamycin (Rapa) at the indicated time points using an unsupervised weighted clustering network analysis (WGCNA) approach. B: Functional over-representation map depicting Molecular Signaling Database (MSigDB) hallmark gene sets associated with each transcriptomic module (Fig. 3c) for cells undergoing etoposide-mediated DDIS in the presence or the absence of rapamycin. C: Heat map showing modules of temporally coexpressed genes for WI38 fibroblasts undergoing RAS-OIS and treated or not with DMOG at the indicated time points using an unsupervised weighted clustering network analysis (WGCNA) approach. For panels A, C, roman numerals refer to different modules. Data are expressed as row Z scores collected from two biologically independent experiments per condition. D: Functional over-representation map depicting Molecular Signaling Database (MSigDB) hallmark gene sets associated with each transcriptomic module (Fig. 3d) for cells undergoing RAS-OIS in the presence or the absence of DMOG. For panels B,D, circles are colour-coded according to the FDR-corrected p value based on the hypergeometric text comparing the overlap between the set of genes in each cluster and the respective list of genes in each MSigDB pathway. Size is proportional to the percentage of genes in the MsigDB gene set belonging to the cluster. N > 100 genes per transcriptomic module for each senescence inducer. Exact values for raw p values, adjusted p values and overlap (absolute and relative) between each pair of sets are reported in Supplementary Table S6 E: River plot depicting the overlaps between gene expression modules in cells undergoing RAS-OIS in the absence or presence of DMOG and cells undergoing DDIS in the absence or presence of rapamycin (Rapa). Blue and red tracks highlight genes down- and upregulated in both senescence perturbation experiments. F: Heat map showing modules of temporally coexpressed metabolites in WI38 fibroblasts undergoing etoposide-mediated DDIS in the presence or the absence of rapamycin for the indicated times using a hierarchical clustering approach. G: Heat map showing modules of temporally coexpressed metabolites for RAS-OIS cells in the presence (+) and absence (-) of DMOG at the indicated time points using a hierarchical clustering approach. For panels F, G, data are expressed as row Z scores collected from three biologically independent experiments per condition and time point.

Rapamycin and DMOG significantly reduced the number of SABG-positive cells by 2.5-fold and fourfold, respectively (Fig. 3a,b). Next, we performed time-resolved gene expression and metabolic profiling to measure rapamycin- and DMOG-mediated changes in the senescence transcriptome and metabolome. Gene clustering, pathway enrichment and gene set enrichment analysis (GSEA) revealed that rapamycin and DMOG shift the transcriptional landscape closer to proliferative control cells, markedly perturbing SASP expression and downregulating the cyclin-dependent kinase inhibitor CDKN1A/p21 (Fig. 3c,d, Extended Data Fig. 5a–d and Supplementary Tables 5–8). We applied a river plot analysis to visualize and quantify the relationship between the two pharmacological treatments. This analysis pinpointed genes in DMOG-treated RAS OIS cells that trend similarly to genes in rapamycin-treated DDIS cells (Extended Data Fig. 5e; blue waves connecting clusters II and III (RAS OIS DMOG) to clusters III and V (DDIS rapamycin), repressed genes; red waves connecting modules I and V (RAS OIS DMOG) to modules IV and VI (DDIS rapamycin), activated genes). In addition, DMOG also provoked a hypoxia-related gene expression programme, which is consistent with its known antagonistic effect on Fe(II)/α-KG-dependent dioxygenases, including hypoxia-inducible factor (HIF) hydroxylases (EGLNs), ribosomal protein hydroxylases (OGFOD), ten-eleven translocation DNA (TETs) and JmjC histone lysine demethylases (KDMs)^23,27,29–32^ (Extended Data Fig. 5c,d; module I).

Congruent with the senomorphic effects on the senescence transcriptome, metabolic profiling demonstrated that rapamycin and DMOG also markedly attenuated SAMS (Fig. 3e,f and Extended Data Fig. 5f,g). In essence, these results highlight that the identified metabolic alterations correlate with the senescence status rather than the nature of the stress inducer and suggest a functional intersection between SAMS and the senescence gene expression programme.

### Glycerol shunt intersects with the senescence programme

**Fig. 4:**
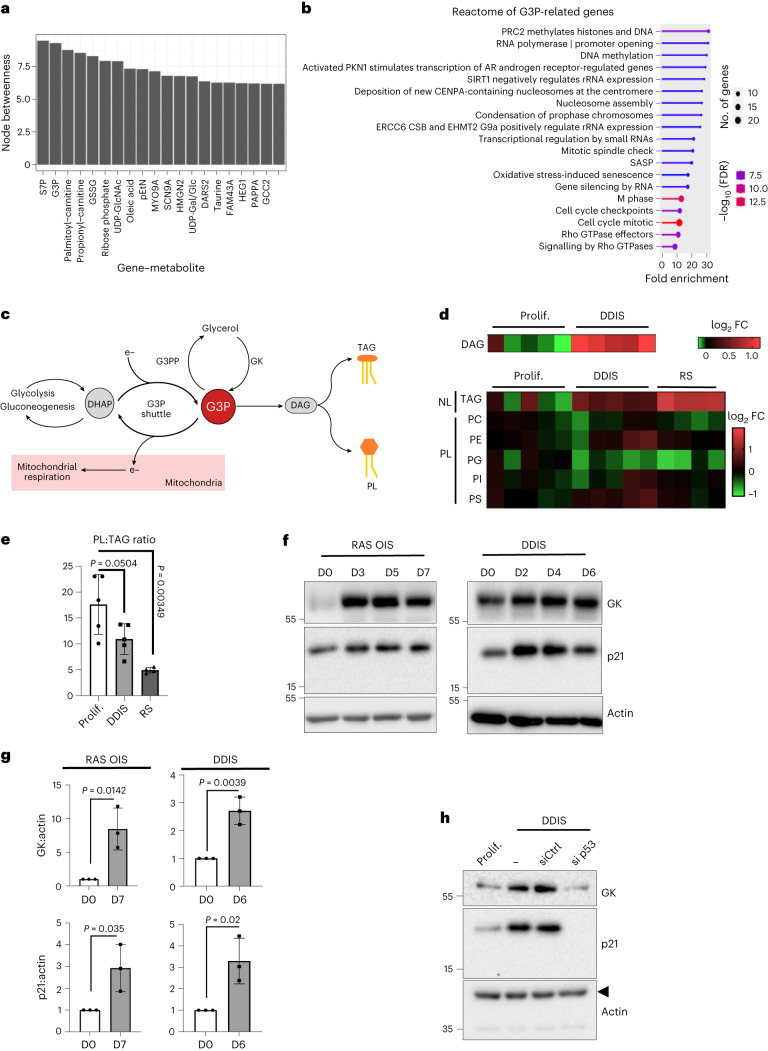
Glycerol-3-P accumulation at the onset of senescence metabolic reprogramming. **a**, Nodes with the highest betweenness values (top 20; Supplementary Table 9) in the gene–metabolite correlation network connecting genes and metabolites presenting a correlation with an absolute value >0.5 for RAS OIS and RAF OIS, etoposide-mediated DDIS, RS and Q. MYO9A, myosin 9-A; SCN9A, sodium voltage-gated channel α subunit 9; HMGN2, high mobility group nucleosomal binding domain 2; DARS2, aspartyl-tRNA synthetase 2, mitochondrial; FAM43A, family with sequence similarity 43 member A; HEG1, heart development protein with EGF-like domains 1; PAPPA, pappalysin 1; GCC2, GRIP and coiled-coil domain containing 2. **b**, Reactome analysis of genes correlating either positively or negatively with G3P accumulation during senescence in WI38 fibroblasts. **c**, Simplified scheme of the metabolic pathways involving G3P. **d**, Heat maps representing levels of indicated lipid species in WI38 fibroblasts proliferating (*n* = 5), undergoing DDIS (*n* = 5) or RS (*n* = 4). PC, phosphatidylcholine; PE, phosphatidylethanolamine; PI, phosphatidylinositol; PS, phosphatidylserine; PG, phosphatidylglycerol; FC, fold change. **e**, Ratio of total PL to TAG levels normalized to protein content in proliferative WI38 fibroblasts compared to DDIS (*n* = 5) or RS (*n* = 4) cells. **f**, Immunoblots showing indicated protein levels in WI38 fibroblasts undergoing RAS OIS (left) or DDIS (right). Sample processing controls (actin) were migrated into different gels from those of GK. **g**, Densitometric quantification of GK and p21 protein levels relative to actin from three experiments, including the one shown in panel (**f**), in RAS OIS (day 7) and DDIS (day 6). **h**, Western blots showing indicated protein levels in extracts from WI38 fibroblasts proliferating or undergoing DDIS and non-transfected (−) or transfected with a control non-silencing siRNA (siCtrl) or an siRNA targeting the p53 mRNA (si p53) for 4 days. The experiment was repeated independently twice with similar results. In **e**,**g**, data are presented as mean ± s.d. Indicated *P* values were calculated using an unpaired two-sided Student’s *t*-test. Source data

**Extended Data Fig. 6:**
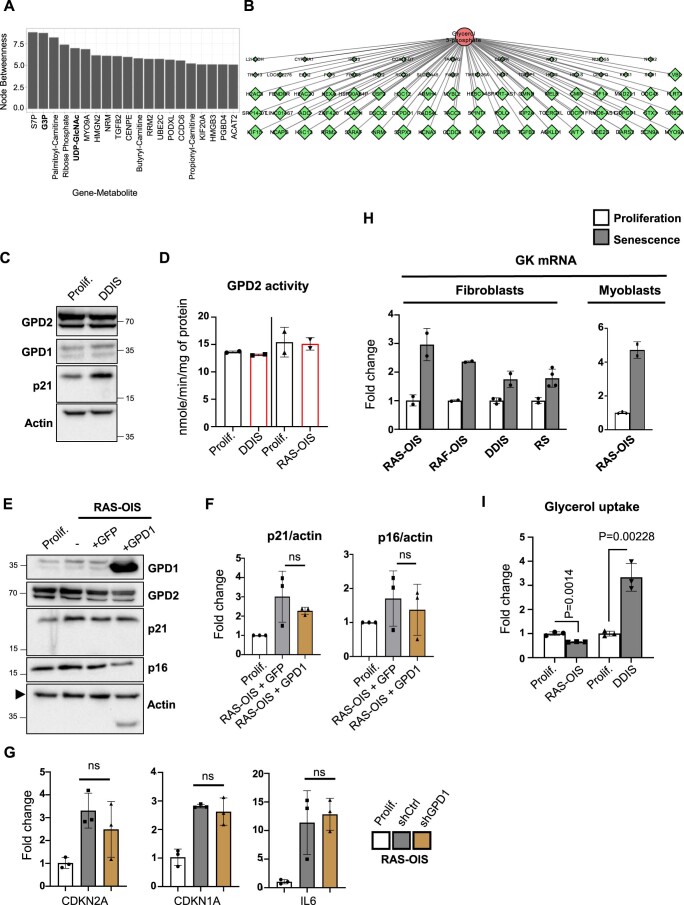
The G3P shuttle is not involved in the establishment of senescence. A: Nodes with the highest betweenness values (top 20) in the overlap network produced by intersecting a Myoblast-only Gene–Metabolite Correlation Network, which considers genes and metabolites presenting a correlation with absolute value higher than 0.5 in myoblast samples, and the Fibroblast Gene-Metabolite Correlation Network, considering all senescence inducers (RAS- and RAF-OIS, DDIS, and RS) and quiescence (Q). B: Genes (green squares) whose expression correlates either positively or negatively with G3P accumulation during senescence in WI38 fibroblasts. The size of green squares is proportional to node betweenness for each target. C: Representative Western blots showing indicated protein levels in extracts of WI38 fibroblasts proliferating or undergoing DDIS for 7 days. D: Activity of mitochondrial Glycerol-3-phosphate dehydrogenase calculated from the measurement of glycerol-3-phosphate cytochrome c reductase in WI38 fibroblasts undergoing DDIS or RAS-OIS during 7 days. n = 2 biological replicates. E: Representative Western blots showing indicated protein levels in WI38 fibroblasts proliferating (Prolif.) or undergoing RAS-OIS induction and not infected (-) or infected with an adenovirus overexpressing GFP or GPD1 for 7 days. F: Densitometric quantification of p16 and p21 protein levels relative to actin from three experiments, including the one of the panel, in proliferative, RAS-OIS cells infected with GFP- or GPD1-overexpressing adenoviruses for 7 days. G: mRNA levels scored by RT–qPCR in WI38 fibroblasts proliferating (Prolif.) or undergoing RAS-OIS and infected with an adenovirus carrying a control scramble shRNA (shCtrl) or an shRNA targeting GPD1 (shGPD1) for 7 days. H: Maximal fold change of the GK mRNA in WI38 fibroblasts and myoblasts induced to senesce under the indicated conditions relative to proliferating cells as measured by Affymetrix microarrays (n = 2 for all the samples, except n = 4 for the senescence sample of RS and n = 3 for proliferative samples of DDIS fibroblasts and RAS-OIS myoblasts). Data are presented as mean values. I: Measurement of glycerol uptake in WI38 fibroblast proliferating, undergoing RAS-OIS (7 days) or DDIS (10 days). The reported values are relative to those of proliferating cells. For panels F, G, E, bars represent the means of 3 biological replicates +/- s.d. Indicated p values were calculated using an unpaired two-sided Student’s t-test. Source data

Measurement and integration of the transcriptome and metabolome in the same cells are increasingly applied to elucidate mechanisms that drive diseases and uncover putative biomarkers (metabolites) and targets (genes). Previous studies have revealed that functionally related genes and metabolites show coherent co-regulation patterns^33,34^. Accordingly, we computed the Spearman correlation between all differentially expressed genes and metabolites, accounting for possible non-linear molecular interactions for the individual senescence subtypes and quiescence. We combined the results obtained from each experiment by selecting gene–metabolite pairs with an absolute correlation higher than 0.5 for all datasets (Supplementary Table 9). Then, we joined these pairs into a gene–metabolite network that connects all molecules with similar profiles over time, regardless of the senescence inducer. The latter allowed us to compute the betweenness centrality, a measure that detects the amount of influence a node has over the flow of information in a graph. Figure 4a shows that S7P and G3P have the highest betweenness centrality in the gene–metabolite network (Supplementary Table 10). This result remains unchanged in the presence of myoblast RAS OIS gene–metabolite network data (Extended Data Fig. 6a). Reactome analysis of G3P-correlated genes (Fig. 4b, Extended Data Fig. 6b and Supplementary Table 11) revealed an association with inflammation and epigenetic regulation of cell cycle genes, thus raising the possibility that G3P acts as a new core ‘hub’ metabolite central to regulating the senescence gene expression programme.

G3P is situated at the crossroads of multiple metabolic pathways. Through the redox conversion to DHAP, G3P can enter glycolysis and gluconeogenesis (Fig. 4c). DHAP reduction to G3P by the G3P dehydrogenase GPD1 regenerates NAD^+^ levels in the cytosolic side of the G3P shuttle, while the mitochondrial side catalysed by GPD2 leads to the formation of DHAP and FADH2 that feeds the electron transport chain. Finally, G3P and FFAs are the critical intermediates for lipogenesis, TAG and PL synthesis. We hypothesized a crucial role of the G3P shuttle in the senescence programme, similar to the malate–aspartate shuttle^35^; however, GPD1 and GPD2 protein levels and GPD2 mitochondrial activity remained unchanged, as exemplified for DDIS and RAS OIS cells (Extended Data Fig. 6c–e). Moreover, adenoviral-mediated GPD1 overexpression or its shRNA-mediated knockdown failed to impact the expression of senescence biomarkers, including CDKN1A/p21, CDKN2A/p16 and IL-6 (Extended Data Fig. 6e–g). These findings thus rule out an involvement of the G3P shuttle in regulating senescence.

Given its role in lipid synthesis, we tested the implication of G3P in the lipid metabolism of senescent cells. Lipidomic analysis revealed a substantial increase of diacylglycerol (DAG) in DDIS compared to control cells. DAG serves as a precursor of neutral lipids (NLs) TAG and PL. Notably, both RS and DDIS also led to the accumulation of TAG. In contrast, the levels of different PL species were not affected substantially compared to control cells, except for decreased phosphatidylglycerol (PG) levels (Fig. 4d). Consequently, the amount of total PL relative to total TAG (NL) was reduced in senescent cells (Fig. 4e). Our data support a view in which senescent cells divert available resources toward converting fatty acids to TAGs stored in lipid droplets^9,12^_._

**Extended Data Fig. 7:**
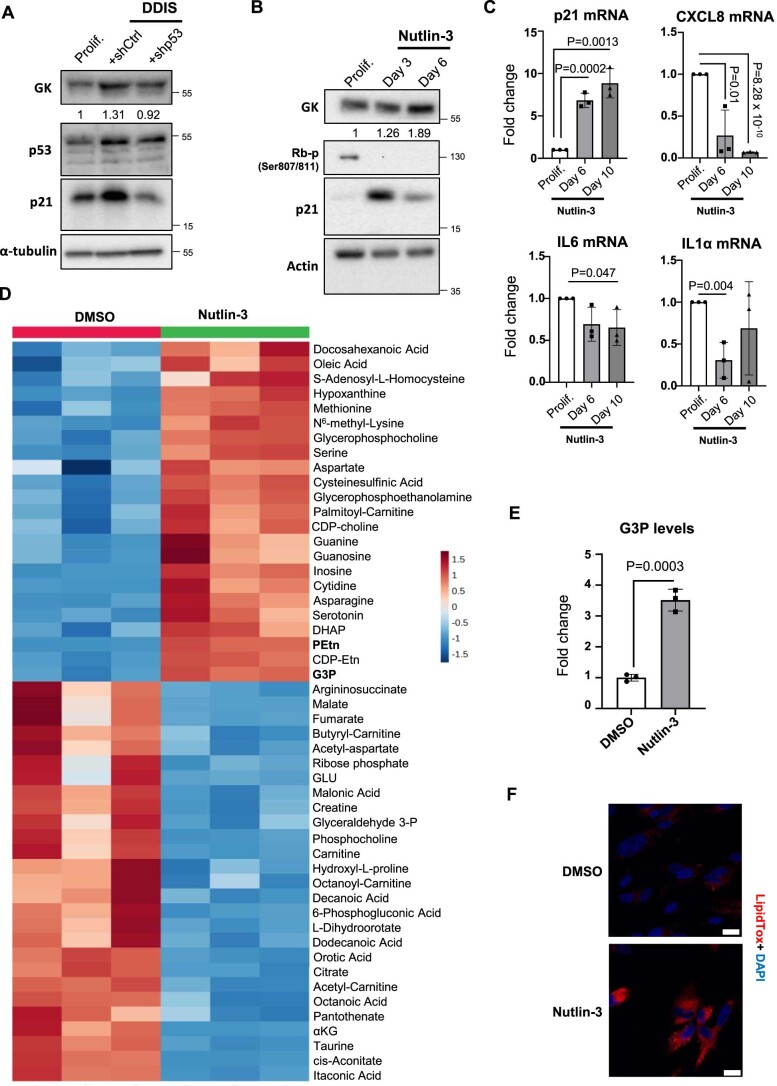
P53 activation recapitulates part of the SAMS. A: Immunoblots showing the levels of the indicated proteins in WI38 fibroblasts proliferating (Prolif.), or undergoing DDIS and infected with an adenovirus driving the expression of a control shRNA (shCtrl) or an shRNA targeting p53 for 7 days. The numbers below the top panel are the quantification of GK levels relative to those of α-tubulin. The experiment was repeated independently twice. B: Immunoblots blots showing the indicated protein levels in WI38 fibroblasts proliferating and treated with vehicle or Nutlin-3 for 3 or 6 days. The numbers below the top panel are the quantification of GK levels relative to those of Actin. C: Levels of the indicated mRNAs as measured by RT–qPCR in WI38 fibroblasts proliferating and treated with vehicle or Nutlin-3 for 6 and 10 days. The reported values are relative to those of proliferating cells. Bars represent the means of 3 biological replicates +/- s.d. Indicated p values were calculated using an unpaired two-sided Student’s t-test. D: Representative heat map using hierarchical clustering showing metabolites in WI38 fibroblasts treated with DMSO or nutliln-3 for 7 days. Data are expressed as row Z scores collected from three biologically independent experiments per condition. E: Fold change of G3P levels in WI38 fibroblast treated with DMSO or nutliln-3 for 7 days, relative to the value of DMSO-treated cells. Bars represent the means of 3 biological replicates +/- s.d. Indicated p values were calculated using an unpaired two-sided Student’s t-test. F: Representative images of DAPI and LipidTox staining of WI38 fibroblasts treated with DMSO or nutlin-3 for 7 days. The experiment was repeated independently twice with similar results. Scale bars represent 20 µm. Source data

GK expression levels were upregulated in all senescence models (Extended Data Fig. 6h), an effect that was confirmed at the protein level in RAS OIS and DDIS (Fig. 4f,g). In contrast, the invariable increase in GK expression was not mirrored by consistent glycerol uptake changes that were downregulated in RAS OIS and increased in DDIS (Extended Data Fig. 6i). The tumour suppressor p53 regulates GK expression, as demonstrated by siRNA- or shRNA-mediated depletion of p53 in DDIS cells (Fig. 4h and Extended Data Fig. 7a). GK catalyses the phosphate transfer from ATP to glycerol to form G3P, controlling whether G3P, a critical intermediate at the crossroad of carbohydrate, lipid and energy metabolism, leaves the cell as glycerol upon its direct dephosphorylation or enters intracellular metabolic pathways. To corroborate the link between p53, GK and G3P accumulation, we analysed the effects of pharmacological activation of p53 by the small-molecule MDM2 antagonist Nutlin-3 (ref. ^36^). This treatment was sufficient to activate p21 expression and upregulate GK levels (Extended Data Fig. 7b,c). Consistent with published data^37–39^, p53 activation suppressed SASP biomarker IL-1α, IL-6 and CXCL8 expression (Extended Data Fig. 7c). In contrast and congruent with other senescence inducers, p53 activation led to a SAMS (Extended Data Fig. 7d), including a rise in G3P levels (Extended Data Fig. 7e) and neutral lipid droplet accumulation (Extended Data Fig. 7f). We conclude that p53 controls a senescence programme involving a p21-dependent cell cycle arrest, GK upregulation, concomitant G3P accumulation and a SAMS independently of its suppressive effect on the SASP.

**Fig. 5:**
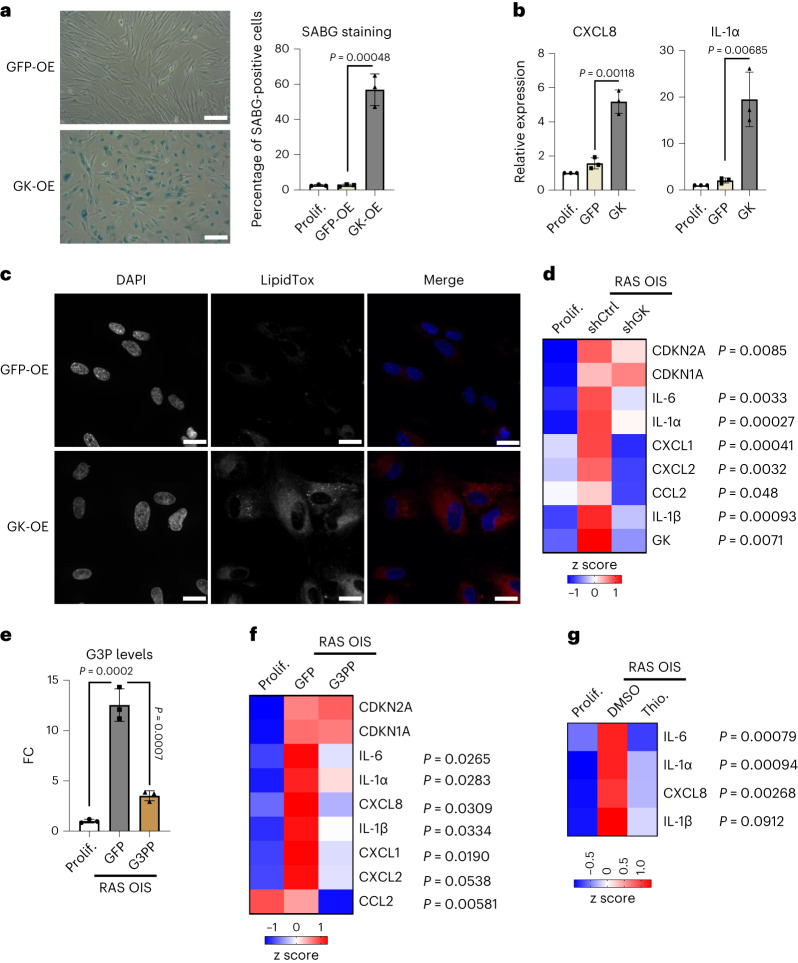
Glycerol-3-P accumulation drives metabolic senescence programme and SASP induction. **a**, Representative images (left) and percentage (right) of SABG-positive WI38 fibroblasts infected with GFP-OE or GK-OE adenoviruses for 7 days. The percentage is also reported for control, non-infected proliferating cells. *n* = 3 biologically independent experiments. Data are presented as mean ± s.d. Indicated *P* values were calculated using an unpaired two-sided Student’s *t*-test. Scale bars, 50 µm. **b**, mRNA levels of the indicated SASP markers as measured by RT–qPCR in WI38 fibroblasts treated as in **a**, relative to the value of non-infected cells (Prolif.). *n* = 3 biologically independent experiments. Data are presented as mean ± s.d. Indicated *P* values were calculated using an unpaired two-sided Student’s *t*-test. **c**, Representative images of 4,6-diamidino-2-phenylindole (DAPI) and LipidTox staining of WI38 fibroblasts infected with GFP-OE or GK-OE adenovirus for 7 days. The experiment was repeated independently three times with similar results. Scale bars, 20 µm. **d**, Heat map of the indicated mRNA levels as measured by RT–qPCR in WI38 fibroblasts proliferating or undergoing RAS OIS and infected with an adenovirus driving the expression of a control scramble shRNA (shCtrl) or an shRNA targeting GK mRNA (shGK) for 7 days (*n* = 3). Indicated *P* values were calculated using an unpaired two-sided Student’s *t*-test between shCtrl and shGK conditions. **e**, FC of G3P levels in WI38 fibroblasts undergoing RAS OIS and infected with GFP-OE or G3PP-OE adenoviruses for 7 days, relative to the value of non-infected cells (Prolif.). *n* = 3 biologically independent experiments. Data are presented as mean ± s.d. Indicated *P* values were calculated using an unpaired two-sided Student’s *t*-test. **f**, Heat map of the indicated mRNA levels as measured by RT–qPCR in WI38 fibroblasts treated as in **e** (*n* = 3). *P* values (unpaired two-sided Student’s *t*-test) in gene expression between GFP and G3PP are indicated. **g**, Heat map of the indicated mRNA levels as measured by RT–qPCR in WI38 fibroblasts subjected to RAS OIS and treated with dimethylsulfoxide (DMSO) or 1-thioglycerol (1 mM) for 7 days, relative to the value of non-infected cells (Prolif.) (*n* = 3). Indicated *P* values were calculated using an unpaired two-sided bilateral Student’s *t*-test between DMSO and 1-thioglycerol conditions. Source data

**Extended Data Fig. 8:**
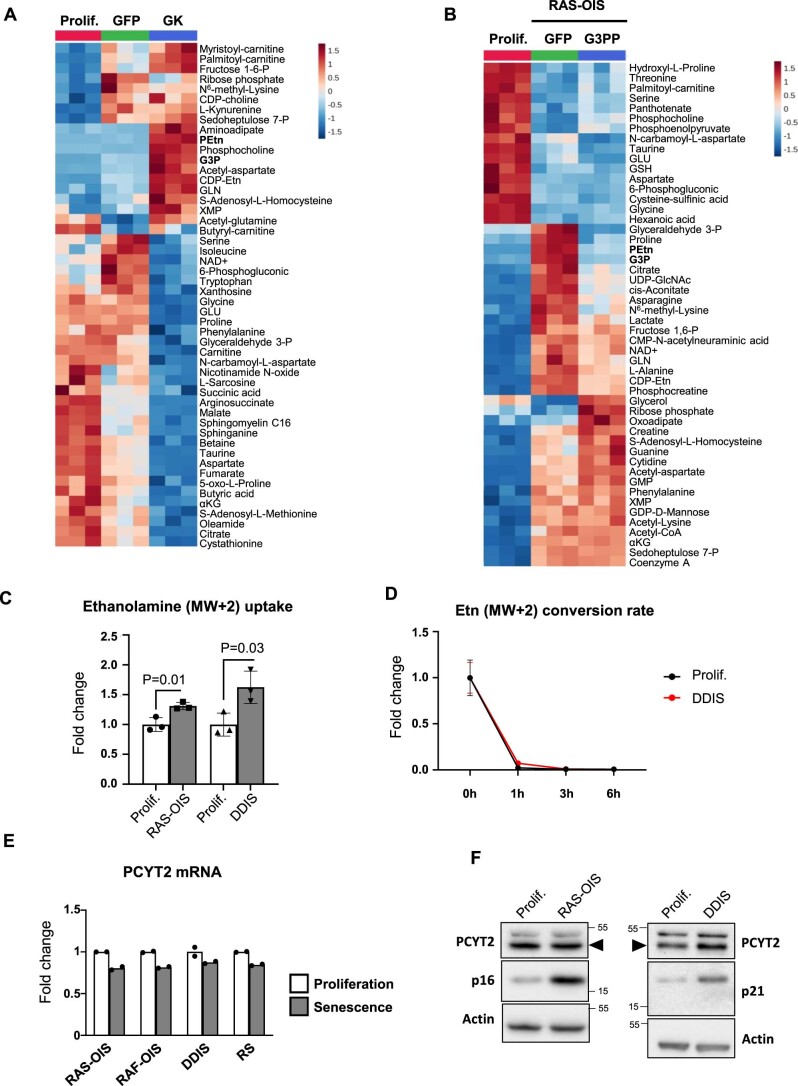
Effects of perturbing G3P levels on metabolome of senescent cells and characterization of the pEtN synthesis pathway. A: Heat map showing modules of metabolites in WI38 fibroblast proliferating or infected with a GFP- or GK-overexpressing adenovirus for 7 days. B: Heat map showing modules of metabolites in WI38 fibroblast proliferating or undergoing RAS-OIS and infected with a GFP- or G3PP-overexpressing adenovirus for 7 days. For both panels, a hierarchical clustering approach was used. Data are expressed as row Z scores collected from three biologically independent experiments per condition. C: Measurement of labelled Etn uptake in WI38 fibroblasts proliferating, undergoing DDIS or RAS-OIS for 7 days after a pulse of 1 hour. The reported values are relative to those of proliferating cells. Bars represent the mean of 3 biological replicates +/- s.d. Indicated p values were calculated using an unpaired two-sided Student’s t-test. D: Curves of decay of labelled Etn in WI38 fibroblasts proliferating or undergoing DDIS, after a pulse of 1 hr followed by a chase for the indicated times. For each treatment the values are normalized to those of the 0 hr chase time. Each point represents the mean of 3 biological replicates +/- s.d. Indicated p values were calculated using an unpaired two-sided Student’s t-test. E: Maximal fold change of PCYT2 mRNA in cells induced to senesce under the indicated conditions relative to proliferating cells as measured by Affymetrix microarrays (n = 2 for each sample). F: Representative Western blots showing the indicated protein levels in WI38 fibroblasts proliferating (Prolif.), undergoing RAS-OIS or DDIS for 7 days. The experiment was repeated independently 3 times with similar results. Source data

To evaluate the functional role of GK in the senescence programme, we first transduced proliferating fibroblasts with an adenovirus overexpressing GK (GK-OE). GK-OE was sufficient to trigger a senescence-like state, as evidenced by the dramatic increase in the percentage of SABG-positive cells and expression of SASP genes CXCL8 and IL-1α (Fig. 5a,b). These effects were accompanied by a considerable accumulation of NLs, consistent with the utilization of G3P in TAG synthesis (Fig. 5c) and a senescence-like metabolic shift, notably of G3P and pEtN levels (Extended Data Fig. 8a). Thus, GK-OE acts senogenic. Conversely, GK knockdown repressed SASP genes in RAS OIS cells. At the same time, p21 and p16 expression were unaffected or minorly affected, respectively (Fig. 5d). Thus, a reduction in GK activity acts as a senomorphic, uncoupling SASP expression and senescence arrest. In line with our above findings, scavenging G3P by overexpressing G3P phosphatase (G3PP-OE)^40^ (forcing G3P conversion to glycerol) had similar effects to GK depletion, reversing G3P accumulation in RAS OIS cells (Fig. 5e and Extended Data Fig. 8b), concomitant with a downregulation of a select number of SASP genes (Fig. 5f). Finally, pharmacological treatment with thioglycerol, a competitive inhibitor of GK^41^, also reduced SASP factors such as IL-1α, CXCL8 and IL-6 in RAS OIS (Fig. 5g). Together, these results reinforce the crucial role of G3P metabolism in senescence regulation.

### G3P and pEtN homoeostatic switch regulates senescence

**Fig. 6:**
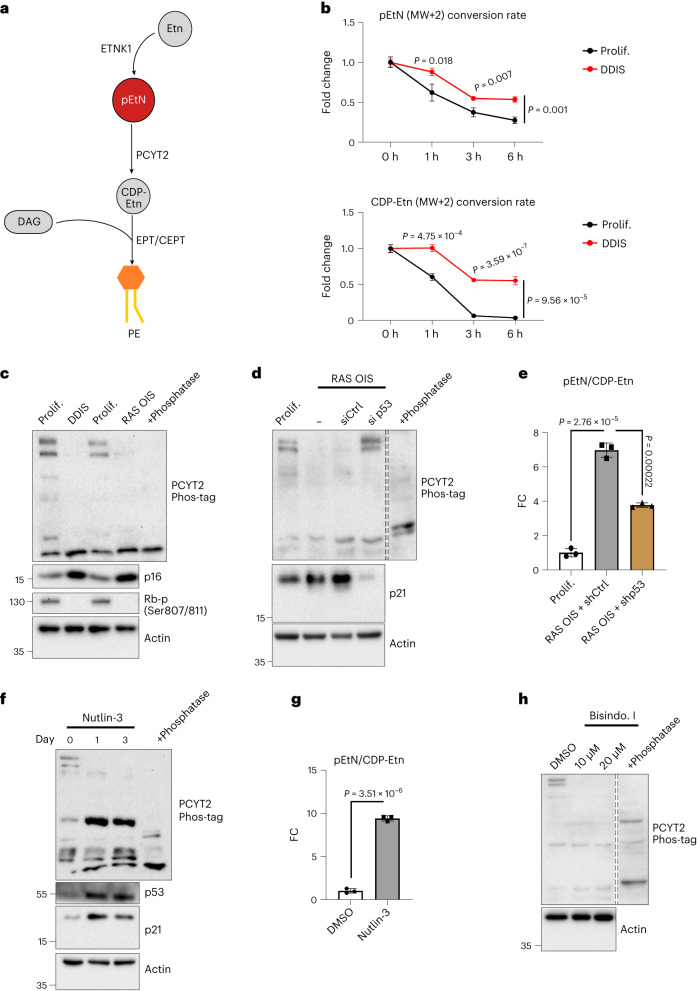
PCYT2 is less active and dephosphorylated in senescent cells. **a**, Schematic overview of the phosphatidylethanolamine pathway highlighting pEtN and the enzymes involved in the pathway. **b**, Curves of decay of labelled pEtN or CDP-Etn in WI38 fibroblasts proliferating or undergoing DDIS (day 10), after a pulse of 1 h followed by a chase for the indicated times. *n* = 3 biological replicates. **c**, Representative western blots of a Phos-tag gel (top) and a conventional gel (bottom) showing the indicated protein levels in extracts from WI38 fibroblasts proliferating, undergoing DDIS (14 days) or RAS OIS (7 days). The phosphatase-treated extract is from proliferating cells. Loading control (actin) was migrated into the same gel as RB. **d**, Representative western blots of a Phos-tag gel (top) and a conventional gel (bottom) showing the indicated protein levels in extracts from WI38 fibroblasts proliferating or undergoing RAS OIS and non-transfected (-) or transfected with a non-silencing siRNA or an siRNA targeting the p53 mRNA for 3 days. The phosphatase-treated extract is from proliferating cells. Dashed lines indicate the cropping of two lanes. Sample processing control (actin) was migrated into a different gel than p21. The experiment was repeated independently twice with similar results. **e**, Fold change of pEtN:CDP-Etn ratio in WI38 fibroblasts undergoing RAS OIS and infected with shCtrl-OE or shp53-OE adenoviruses for 7 days, relative to the value of non-infected cells (Prolif.). *n* = 3 biologically independent experiments. **f**, Representative western blots of a Phos-tag gel (top) and a conventional gel (bottom) showing the indicated protein levels in extracts from WI38 fibroblasts treated by Nutlin-3 (10 µM) for the indicated times. The phosphatase-treated extract is from proliferating cells. The experiment was repeated independently twice with similar results. **g**, FC of pEtN:CDP-Etn ratio in WI38 fibroblasts treated by Nutlin-3 (10 µM) for 7 days. *n* = 3 biologically independent experiments. **h**, Representative western blots of a Phos-tag gel (top) and a conventional gel (bottom) showing the indicated protein levels in extracts from WI38 fibroblasts treated with BisIndo.I at the indicated concentrations for 16 h. Dashed lines indicate the cropping of one lane. For **b**,**e**,**g**, data are presented as mean ± s.d. All the indicated *P* values were calculated using an unpaired two-sided Student’s *t*-test. For **c**,**h**, the experiment was repeated independently three times with similar results. Source data

G3P and pEtN are building blocks for TAG and PL synthesis and accrue in senescent cells. pEtN is utilized in the Kennedy pathway for the biosynthesis of phosphatidylethanolamine (PE), a major component of cell membranes accounting for 25–35% of total PL. Ethanolamine (Etn) is first taken up by cells, subsequently phosphorylated to pEtN by ethanolamine kinase 1 (ETNK1) and finally conjugated to CDP by phosphate cytidylyltransferase 2, ethanolamine (PCYT2) to react with DAG to generate PE by ethanolaminephosphotransferases (EPT and CEPT) (Fig. 6a)^42^.

To probe the role of Etn metabolism in senescence, we first performed flux experiments with exogenous ^13^C-Etn (Etn MW + 2). We did not detect differences in Etn uptake and Etn conversion between proliferating and senescent cells (Extended Data Fig. 8c,d); however, the pEtN and CDP-Etn (MW + 2) conversion were less effective in senescent cells compared to proliferative cells, pointing to decreased Pcyt2 activity (Fig. 6b). Accordingly, we measured the Pcyt2 transcript and protein levels, which were unchanged between proliferating and senescent cells (Extended Data Fig. 8e,f). Previous studies demonstrated that Pcyt2 activity is positively regulated by phosphorylation on several serine and threonine residues^43^. We therefore, determined the Pcyt2 phosphorylation status by Phos-tag analysis. Pcyt2 displayed several band shifts in extracts from proliferating cells, consistent with its phosphorylation on multiple sites (Fig. 6c). Notably, DDIS and RAS OIS cells exhibited a dramatic reduction in Pcyt2 band shifts, indicative of reduced protein phosphorylation compared to control cells. This effect was p53-dependent because (1) p53 silencing (siRNA- or shRNA-mediated) rescued Pcyt2 phosphorylation (Fig. 6d), while reducing the pEtN:CDP-Etn ratio in RAS OIS cells (Figs. 6e) and (2) p53 activation by Nutlin-3 reduced Pcyt2 phosphorylation (Fig. 6f) and increased the pEtN:CDP-Etn ratio in cells (Fig. 6g)^36^. Thus, p53 negatively regulates Pcyt2 phosphorylation and activity in senescence, resulting in pEtN accumulation. PCYT2 phosphorylation was sensitive to treatment with the selective PKC inhibitor bisindolylmaleimide (BisIndo.I) (Fig. 6h). Whether p53 controls PCYT2 phosphorylation by affecting PKC activity or an uncharacterized PCYT2 phosphatase remains to be determined.

**Fig. 7:**
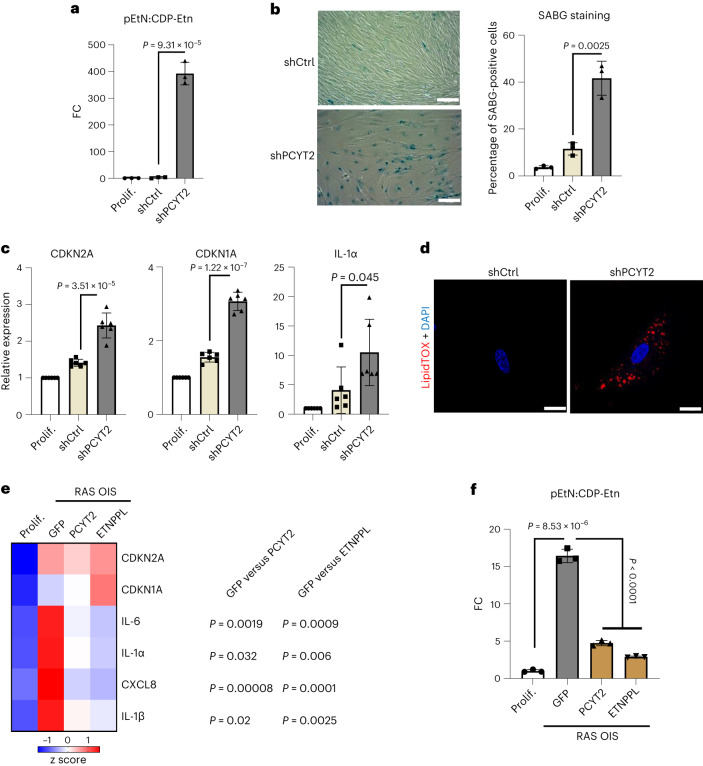
PCYT2 and pEtN modulation regulate the senescence metabolic reprogramming. **a**, FC of pEtN:CDP-Etn ratio in WI38 fibroblasts infected with an adenovirus driving the expression of a control shRNA (shCtrl) or an shRNA targeting the PCYT2 mRNA (shPCYT2) for 7 days, relative to the value of non-infected cells (Prolif.) **b**, Representative images (left) and percentage (right) of SABG-positive WI38 fibroblasts treated as in **a**. The percentage is also reported for non-infected proliferating cells. Scale bars, 20 µm. **c**, mRNA levels of senescence markers scored by RT–qPCR in WI38 fibroblasts treated as in **a**. *n* = 6 biologically independent experiments. Data are presented as mean ± s.d. Indicated *P* values were calculated using an unpaired two-sided Student’s *t*-test. **d**, Representative images of DAPI and LipidTox staining of WI38 fibroblasts infected with an adenovirus driving the expression of a control shRNA (shCtrl) or an shRNA targeting the PCYT2 mRNA (shPCYT2) for 7 days. The experiment was repeated independently three times with similar results. **e**, Heat map of the indicated mRNA levels as measured by RT–qPCR in WI38 fibroblasts proliferating (Prolif.) or subjected to Ras induction and infected with adenoviruses overexpressing GFP, PCYT2 or ETNPPL for 7 days (*n* = 3). *P* values (unpaired two-sided Student’s *t*-test) in gene expression between RAS OIS + GFP and RAS OIS + PCYT2 or between RAS OIS + GFP and RAS OIS + ETNPPL are indicated. **f**, FC of pEtN:CDP-Etn ratio in WI38 fibroblasts treated as in **e**, normalized to the value of non-infected cells (Prolif.). For **a**,**b**,**f**, bars represent the means of three biological replicates ± s.d. Indicated *P* values were calculated using an unpaired two-sided Student’s *t*-test. Source data

**Extended Data Fig. 9:**
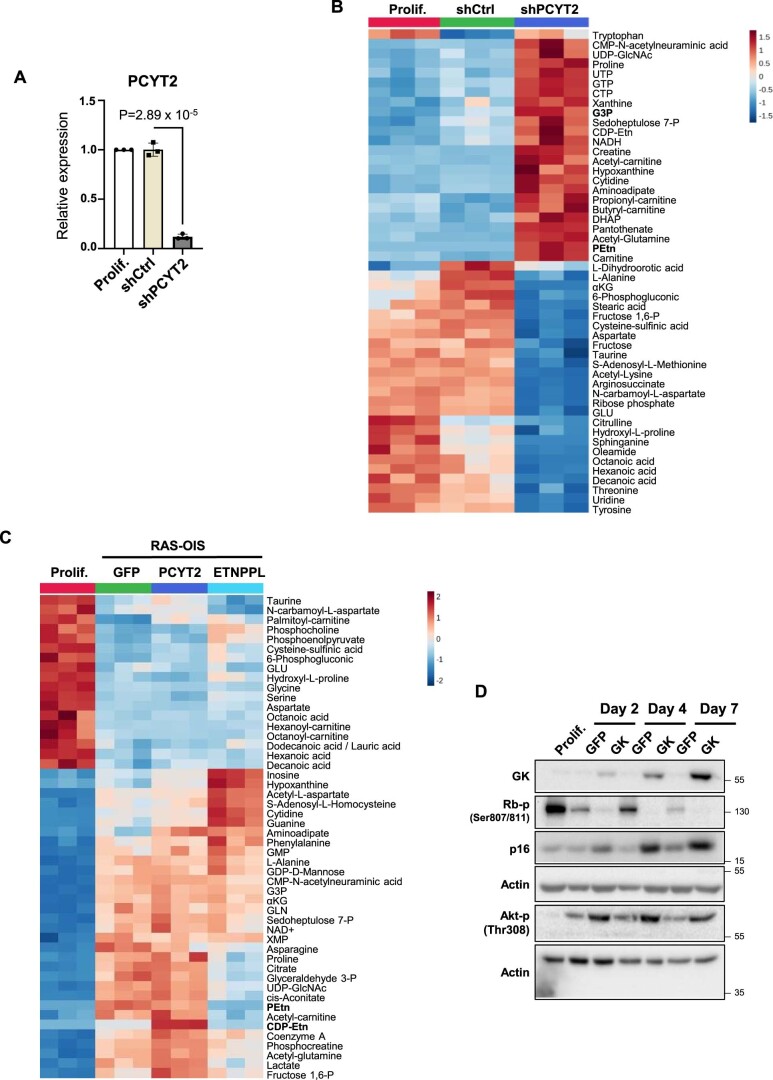
Effects of perturbating pEtN levels on metabolome of senescent cells and upregulation of AKT activity in GK overexpressing cells. A: Levels of the PCYT2 mRNAs normalized to those of RPS14 as measured by RT–qPCR in WI38 fibroblasts proliferating or infected with an adenovirus driving the expression of a control shRNA (shCtrl) or an shRNA targeting the PCYT2 mRNA (shPCYT2) for 7 days. The reported values are relative to those of proliferating cells. Bars represent the means of 3 biological replicates +/- s.d. Indicated p values were calculated using an unpaired two-sided Student’s t-test. B: Representative heat map using hierarchical clustering showing metabolites in WI38 fibroblasts proliferating (Prolif.) and shControl-(shCtrl) or shPCYT2-expressing WI38 fibroblasts at day 7. Data are expressed as row Z scores collected from three biologically independent experiments per condition. C: Representative heat map using hierarchical clustering showing metabolites in WI38 fibroblasts proliferating (Prolif.) or undergoing RAS-OIS and infected with GFP-PCYT2- or ETNPPL overexpressing adenoviruses for 7 days. Data are expressed as row Z scores collected from three biologically independent experiments per condition. D: Representative western blots showing the levels of the indicated proteins in WI38 fibroblasts proliferating or infected with GFP- or GK-overexpressing adenovirus for the indicated times. The experiment was repeated independently 3 times. Source data

To evaluate the functional consequences of pEtN level alterations, we performed Pcyt2 knockdown experiments in proliferating cells, thus blocking pEtN conversion and resulting in an increased pEtN:CDP-Etn ratio (Fig. 7a and Extended Data Fig. 9a,b). Analogous to GK-OE, Pcyt2 knockdown was sufficient to elicit a senescence-like phenotype, as evidenced by an increase in cells staining positive for SABG and in the expression of canonical senescence biomarkers CDKN1A, CDKN2A and IL-1α (Fig. 7b,c). In addition, Pcyt2 knockdown also triggered neutral lipid droplet accumulation (Fig. 7d). By contrast, Pcyt2 OE in RAS OIS cells reduced the expression of SASP factors IL-1α, IL-1β, IL-6 and CXCL8 (Fig. 7e), while re-establishing a pEtN:CDP-Etn ratio similar to that of proliferating control cells (Fig. 7f). To further corroborate that pEtN accumulation in cells directs the senescence fate, we overexpressed ethanolamine-phosphate phospho-lyase (ETNPPL), an enzyme promoting the breakdown of pEtN to ammonia, inorganic phosphate and acetaldehyde^44^. In line with the above results, ETNPPL-OE lowered the pEtN:CDP-Etn ratio (Fig. 7f and Extended Data Fig. 9c), repressing the SASP biomarkers IL-1α, IL-1β, IL-6 and CXCL8 in RAS OIS cells (Fig. 7e). We thus conclude that the senescence phenotype is intricately linked to pEtN homoeostasis.

**Fig. 8:**
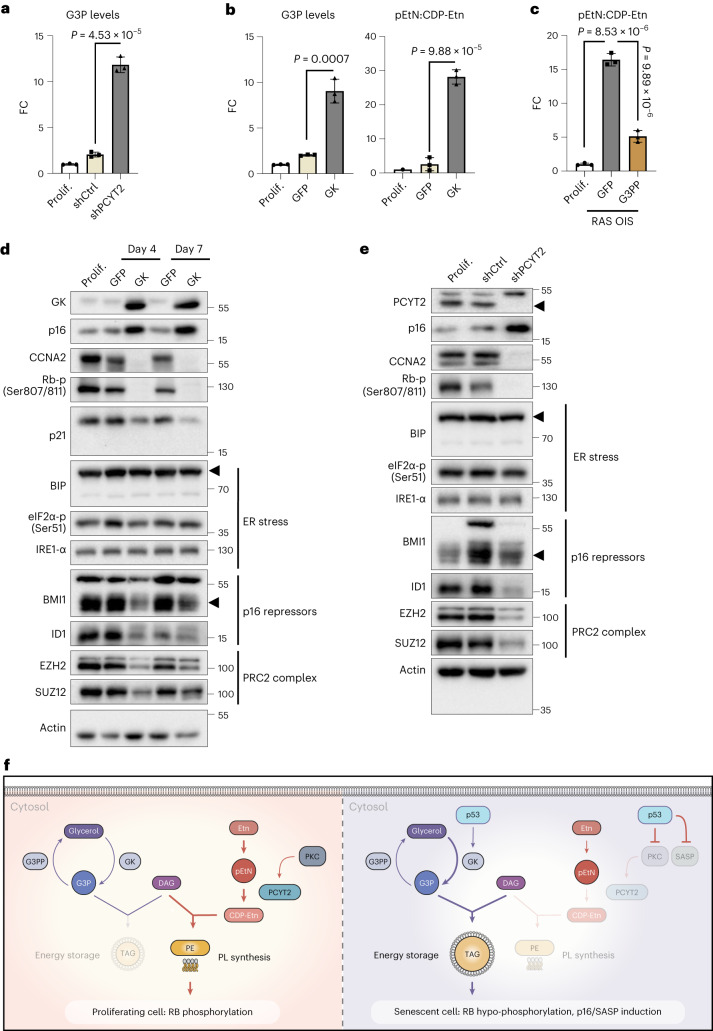
Phosphoethanolamine and G3P accumulation are interconnected and regulate RB phosphorylation during senescence. **a**, FC of G3P levels in WI38 fibroblasts infected with an adenovirus overexpressing a control shRNA (shCtrl) or an shRNA targeting the PCYT2 mRNA (shPCYT2) for 7 days, relative to the value of non-infected cells (Prolif.). *n* = 3 biologically independent experiments. Data are presented as mean ± s.d. Indicated *P* values were calculated using an unpaired two-sided Student’s *t*-test. **b**, FC of G3P levels and pEtN:CDP-Etn ratio in WI38 fibroblasts infected with GFP-OE or GK-OE adenovirus for 7 days, relative to the value of non-infected cells (Prolif.). *n* = 3 biologically independent experiments. Data are presented as mean ± s.d. Indicated *P* values were calculated using an unpaired two-sided Student’s *t*-test. **c**, FC of pEtN:CDP-Etn ratio in WI38 fibroblasts infected with GFP-OE or G3PP-OE adenovirus for 7 days, relative to the value of non-infected cells (Prolif.). *n* = 3 biologically independent experiments. Data are presented as mean ± s.d. Indicated *P* values were calculated using an unpaired two-sided Student’s *t*-test. **d**, Representative western blots showing indicated protein levels in WI38 fibroblasts not infected (Prolif.) or infected with GFP-OE or GK-OE adenoviruses for 4 or 7 days. Loading control (actin) was migrated into the same gel than p16, CCNA2 and GK. The experiment was repeated independently three times with similar results. **e**, Representative western blots showing indicated protein levels in WI38 fibroblasts not infected (Prolif.) or infected with an adenovirus overexpressing a control shRNA (shCtrl) or an shRNA targeting the PCYT2 mRNA (shPCYT2) for 7 days. The arrowhead indicates the position of the band corresponding to the PCYT2 protein. Loading control (actin) was migrated into the same gel as CCNA2 and RB. The experiment was repeated independently three times with similar results. **f**, Representation of G3P and pEtN metabolic interconnections leading to TAG accumulation and senescence. Source data

To decipher how senescence rewired PL metabolism, we revisited our lipidomic analysis. Despite decreased Pcyt2 activity, the phosphatidylethanolamine (PE) level in senescent cells was not altered (Fig. 4d). A similar observation was recently made in Pcyt2^+/−^ cells and could be explained by decreased PE degradation and increased conversion of other PL to PE^45^. Notably, Pcyt2 knockdown recapitulated the alterations of glycerolipid metabolism by senescence inducers, as evidenced by the accumulation of G3P (Fig. 8a) and lipid droplets (Fig. 7d). Similarly, the modulation of G3P levels by GK or G3PP OE affected pEtN levels (Fig. 8b,c), indicating a homoeostatic interconnection between G3P and pEtN in the cell. At the molecular level, the increase in G3P and pEtN promoted CDKN2A/p16 accumulation, hypo-phosphorylation (activation) of RB and the consequent repression of the pro-proliferative RB/E2F target gene Cyclin A2 (*CCNA2*) (Fig. 8d,e), with no sign of ER stress. The inhibitory effect of GK overexpression on RB phosphorylation was rapid, already detectable 2 days after viral transduction, indicating a role of the G3P–pEtN switch in the induction of senescence (Extended Data Fig. 9d). Of note, p16 transcriptional upregulation was accompanied by substantial downregulation of the Id1 transcription factor and Polycomb Group complex 1 and 2 (PRC1 and PRC2) components Bmi1, Ezh2 and SUZ12 (Fig. 8d,e) implicated in p16 repression^46^. GK rapidly activated Akt phosphorylation (Extended Data Fig. 9d), a known negative regulator of PRCs^47,48^. Mechanistically, these data suggest that the G3P–pEtN switch remodels the epigenetic and transcriptional landscape to allow p16 transcriptional activation.

**Extended Data Fig. 10:**
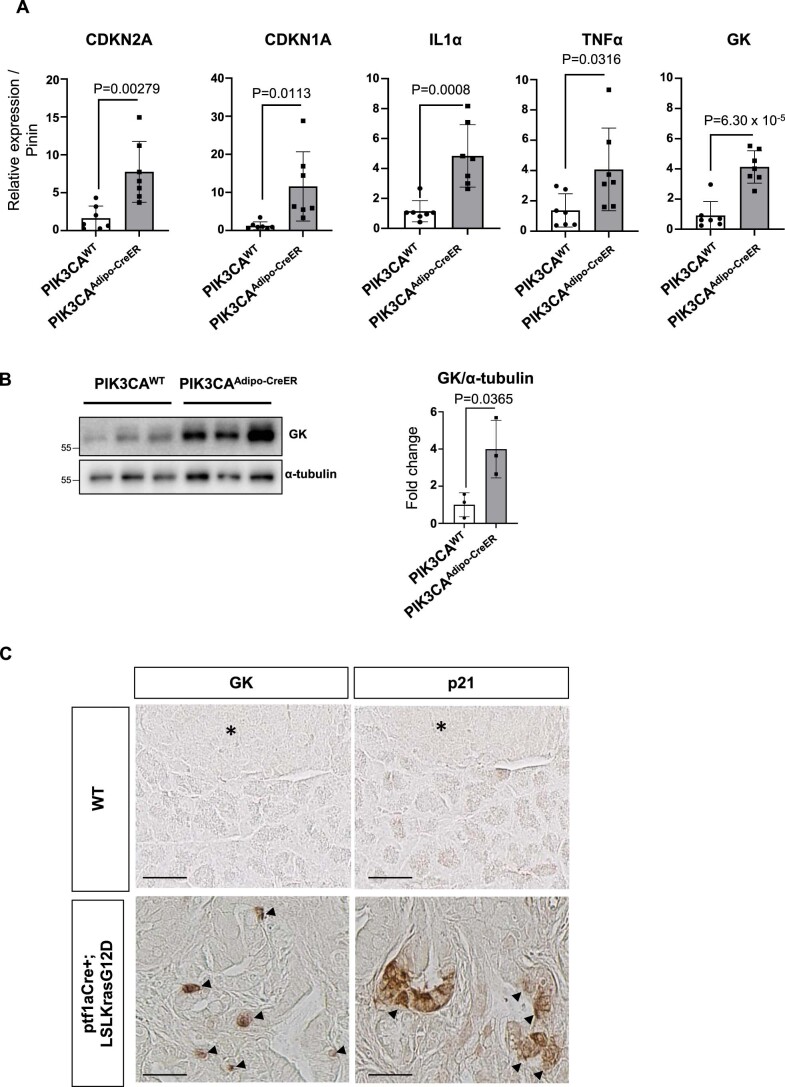
GK upregulation in in-vivo models of senescence. A: Levels of the indicated mRNAs normalized to those of Pinin as measured by RT–qPCR in mouse adipose tissue not expressing (PIK3CAWT) or expressing (PIK3CAAdipo-CreER) the constitutively active PI3KCA mutant. The reported values are relative to those of the PIK3CAWT genotype. Bars represent the means of n = 7 biological replicates (9 males and 5 females) +/- s.d. Indicated p values were calculated using an unpaired two-sided Student’s t-test. B: Left panel: Representative western blot showing the levels of the indicated proteins in mouse adipose tissue not expressing (PIK3CAWT) or expressing (PIK3CAAdipo-CreER) the constitutively active PI3KCA mutant. Right panel: Levels of the GK protein normalized to those of α-tubulin as measured on western blots on total protein extracts of mouse adipose tissue not expressing (PIK3CAWT) or expressing (PIK3CAAdipo-CreER) the constitutively active PI3KCA mutant. Values are normalized to those of the PIK3CAWT sample. Bars represent the means of 3 biological replicates +/- s.d (4 males and 2 females). Indicated p values were calculated using an unpaired two-sided Student’s t-test. C: Immunostaining with an anti-p21 and an anti-GK antibody on sections of the pancreas of WT mice (male) and of mice overexpressing in the pancreas the constitutively active G12D KRas-mutant (male). Asterisks indicate Langerhans islets. Scale bars represent 20 µm. The experiment was repeated independently twice with similar results. Source data

To extend these observations to pathophysiological conditions, we measured GK expression in senescence-prone mouse models (Extended Data Fig. 10). These included PIK3CA^Adipo-CreER^ mice that display white adipose tissue (WAT) hypertrophy upon tamoxifen-induced expression of constitutively active PIK3CA/AKT in WAT^49^ and LSL-KrasG12D^Ptf1a-Cre^ transgenic mice, which develop spontaneous pancreatic premalignant lesions containing senescent cells^50^. PIK3CA mutant transgenic mice showed increased GK expression in their WAT coinciding with upregulated senescence biomarkers p16, p21, IL-1α and tumour necrosis factor α (Extended Data Fig. 10a,b).

In LSL-KrasG12D^Ptf1a-Cre^ transgenic mice, GK and p21 senescence biomarkers were detectable in the pancreatic intraepithelial neoplasia (PanIN) of KrasG12D-expressing mice but not in control wild-type mice (Extended Data Fig. 10c). While these data do not establish a causal relationship between GK-OE and senescence in vivo, they indicate that a GK-dependent switch may operate in tissues undergoing senescence.

## Discussion

In this study, we performed a multi-layered kinetic analysis combining transcriptomics and metabolomics of human fibroblasts senescent cells to reveal common metabolic adaptations and the underlying gene expression mechanisms across various stresses that promote senescence. We identified a universal signature of SAMS, pointing to the accumulation of lactate versus pyruvate, α-KG versus succinate, G3P and pEtN. We focused on G3P and pEtN, which led us to reveal two newly observed mechanisms responsible for their accumulation in senescent cells: the upregulation of GK levels and the post-translational downregulation of Pcyt2, respectively. We found that G3P and pEtN are not only biomarkers of the senescent state but are also potent inducers. Promoting or scavenging G3P and pEtN accumulation by modulating the expression of GK and G3PP and Pcyt2 and ETNPPL, is necessary and sufficient to impact the senescent fate decision. G3P and pEtN levels are homeostatically interdependent and coordinated, suggesting that they represent a core hub node in the balance between NLs and PLs (Fig. 8f).

Our identified metabolic biomarkers are associated with senescence rather than the induction of cellular stress. Of note, exposing cells to cellular stress in the presence of drugs blunting the senescence programme is sufficient to curtail the rise in the level of these biomarkers. The mTOR inhibitor rapamycin is a well-known anti-senescence treatment in vitro^24,26^, promoting longevity in several experimental in vivo models^51^. Here, we report that DMOG also has decisive effects on senescence and the SAMS. The rationale for testing DMOG is the α-KG accumulation in senescent cells, previously observed in cancer cells from KRAS-mutant mouse models of pancreatic cancer upon restoring p53 expression^23^. DMOG acts as an antagonist of α-KG on Fe(II)/α-KG-dependent dioxygenases^27^, a large family of enzymes regulating cell fate through the control of metabolism and epigenetics. DMOG is a potent inducer of HIF-dependent transcription by inhibiting the Fe(II)/α-KG-dependent dioxygenases involved in HIF degradation. Our transcriptomics analysis identified major changes in the hypoxia response during senescence; however, at this stage, we cannot rule out additional targets explaining the effects of DMOG on senescence. Moreover, DMOG has been reported to have HIF-independent effects on mitochondrial metabolism^52^. Future studies should address whether the mechanism of action of DMOG requires HIF and/or other cellular components.

One rationale for this study was that comparing metabolomics and transcriptomics data across various senescent inducers would reveal key steps for senescence initiation. For instance, lipid droplet accumulation is a common feature observed in a wide range of senescence conditions, both in vitro and in vivo; however, the routes leading to lipid droplet accumulation may differ depending on the cellular insults. Increased lipid uptake, de novo lipogenesis, decreased fatty acid oxidation and lipid remodelling by lysosomal PL degradation all trigger lipid droplet formation. Indeed, we identified distinct acylcarnitine derivatives as molecules that lead to the highest discrimination between CS inducers; however, their contribution has been shown to vary in different experimental models^8,12,15^. Oncogenic Ras and hypoxic cells mainly rely on lipid uptake and fatty acid scavenging, whereas PI3K and Akt actively turn on de novo lipid biosynthesis and mitochondrial mutations somewhat impair FAO^11,53,54^. Thus, the modulation of de novo lipogenesis by FAS, SCD1 and ACC activities may have different outcomes when the driver of senescence is oncogenic Ras as opposed to replicative or oxidative stresses^11,16^. Here, we find that the G3P and pEtN accumulation is invariably detected in all the senescence settings that we tested. Moreover, their levels affect lipid droplet formation, suggesting that they represent obligatory steps in this phenotype. These observations can be extended to other systems and cell types in which pEtN and G3P alterations were also reported^10,15,55^. Considering this robust response, we addressed the origins and functional consequences of pEtN and G3P status, as discussed below.

G3P and pEtN are the products of the activation step of glycerol and ethanolamine initiating TAG and PE synthesis. Glycerol is produced intracellularly by lipolysis or via the newly discovered enzyme G3PP^40^ and can also cross the membrane through aquaglyceroporins channels, including AQP3, AQP7, AQP9 and AQP10 in mammals^56^. Etn is a nutrient present in food sources from plants, where it is derived from PE lipolysis or decarboxylation of serine^42^. Etn, like choline, permeates the CTL1 and CTL2 membrane channels^57^. While pEtN specifically enters the Kennedy pathway for PE biosynthesis, G3P is involved in several metabolic pathways, such as lipid synthesis, glycolysis, gluconeogenesis and the electron transport chain. Although we cannot exclude the contribution of multiple pathways, our data suggest that the increase of GK activity and TAG synthesis is a major cause and consequence of G3P accumulation in senescent cells.

Several lines of evidence in the literature suggest that the modulation of G3P and pEtN in vivo affects age-related diseases, including metabolic syndromes. Thus, Pcyt2 expression negatively correlates with obesity^45^. The heterozygous deletion of Pcyt2 in mice leads to hepatic DAG and TAG accumulation, providing a model of non-alcoholic steatohepatitis. Pcyt2 activity declines in aging muscles of mice and humans, consistent with Pcyt2 deficiency causing muscle weakness and aging^58^. Similarly, G3PP suppression increases lipid synthesis, reduces FAO and lowers ATP levels in liver and pancreatic β cells^40,59,60^. In *Caenorhabditis* *elegans*, three phosphoglycolate phosphatase homologue (PGPH) enzymes have been proposed as G3PP orthologues^61^. Their combined deletion increases G3P levels without affecting other proposed PGPH substrates, such as 2-phosphoglycolate, 2-phospholactate and 4-phosphoerythronate. This leads to increased fat deposition and lethality in hyperosmotic stress conditions and high glucose exposure, in which the mutant worms cannot produce glycerol and become hypersensitive to these stresses.

Conversely, OE of PGPH-2 triggers a mild increase in *C.* *elegans* lifespan, accompanied by decreased fat content. Notably, the glycerol channel aqp1 in *C.* *elegans* is implicated in the lifespan-shortening effect of a glucose diet^62^. Although senescence has not been characterized in *C.* *elegans*, some of the pathways regulating lifespan in worms have been linked to a senescence phenotype in higher organisms. In mammals, insulin represses the expression of aquaglyceroporin channels and AQP7-deficient mice display obesity and insulin resistance because their glycerol permeability is affected^63^. Our study linking G3P and pEtN accumulation to the senescence programme in human cells may explain this age-related pathophysiology in vivo.

We propose that G3P and pEtN are central to the homoeostatic switches orchestrating the senescence programme by modifying the balance between TAG and PLs. The high level of coordination and interdependence between these two metabolites is demonstrated by the findings that the GK and G3PP treatments affecting G3P similarly influence pEtN levels, while the Pcyt2 treatment affecting pEtN levels influences G3P levels. Our lipidomic analyses show that the balance shifts toward NLs in senescent cells compared to PLs^15^. In addition, both GK overexpression and Pcyt2 downregulation lead to TAG accumulation. The impairment of Pcyt2 activity in senescent cells has mild effects on the composition of membrane PLs, while promoting TAG accumulation. The latter is consistent with the analysis of Pcyt2 heterozygous mutant mice, displaying TAG accumulation with constant PE levels^45,64^. The reduced flux in the Kennedy pathway for PE biosynthesis is compensated by PS decarboxylation and the reduced turnover of the membrane phospholipids. Of note, the sole PL to be significantly downregulated in senescent cells is PG, again pointing to the interconnection with G3P metabolism.

The coordination between G3P and pEtN is also achieved by interacting with the two master regulators of the senescence programme, p53 and p16/RB. We find that p53 controls GK and Pcyt2 activities, leading to G3P and pEtN accumulation. In turn, these two metabolites implement a downstream senescent response characterized by sharp p16/RB changes in the face of a relatively constant p53 activity. In addition, p53 upregulates GK messenger RNA levels, consistent with previous studies in HepG2 cells expressing shRNA against p53 or treated with Nutlin^65^. Notably, GK expression in HepG2 cells is accompanied by the upregulation of AQP3 and AQP9 in glycerol uptake. The transcriptional effect of p53 may be indirect, as chromatin immunoprecipitation experiments failed to identify p53 response elements in the promoter of these genes^65^. We also cannot exclude the increased stability of the GK transcript in senescent cells. The control of Pcyt2 activity by p53 is not transcriptional but post-translational and is accompanied by the dephosphorylation of the enzyme in senescent cells. Pcyt2 activity is regulated by phosphorylation on several residues, including two putative PKC sites, Ser-197 and Ser-205 (ref. ^43^). Mutation of both serine residues to alanine decreases enzymatic activity, whereas phorbol ester treatment upregulates Pcyt2 activity. Future studies should determine by what mechanism p53 leads to Pcyt2 dephosphorylation, for example, whether it involves kinase inhibition or phosphatase activation. Some PKC isoforms are DAG-dependent, suggesting another possible link with lipid homoeostasis.

Ectopically increasing G3P levels alone can elicit a senescence-like response without additional stresses. Few metabolic adaptations have such a potent effect. Electron transport chain inhibition, malate dehydrogenase knockdown and inhibition of malic enzymes ME1/ME2 can drive a senescence programme directly related to mitochondrial activity^13^; however, GK and Pcyt2 are cytosolic enzymes mainly controlling lipid synthesis in this setting. pEtN, G3P and the metabolites in lipid droplet biosynthesis may impact mitochondrial activity, though this is an indirect response to a cytosolic modification. pEtN has been proposed to inhibit mitochondrial activity through competition with succinate at complex II (or succinate dehydrogenase) of the mitochondrial respiratory chain^66^. Lipid droplets could also alter ER–mitochondrial contact sites, though we did not detect signs of ER stress upon GK and Pcyt2 modulation. The pathway linking G3P and pEtN to the transcriptional and epigenetic machinery regulating senescence remains unknown.

The universal metabolic adaptations across various senescence inducers described in our paper suggest new avenues of therapeutic interventions. GK, Pcyt2 and G3PP have enzymatic activities that are druggable. Meclizine has been reported as a Pcyt2 inhibitor^67^. Thioglycerol, used in our study and (+/-)-2,3-dihydroxypropyl-dichloroacetate act as GK inhibitors^41^. Although their chemistry is not compatible with in vivo treatments, they could serve to model further drug development. GK inhibition is predicted to increase the level of intracellular glycerol, which is less toxic than G3P. Their efflux through aquaglyceroporins would reduce carbon sources for oxidation and lipogenesis, potentially blunting senescence response, inflammatory cytokine production and age-related disorders^56^.

### Limitations of the study

Although we provide compelling evidence that preventing G3P and pEtN accumulation exerts senomorphic effects in cells exposed to senescence-inducing agents, we did not address whether reducing G3P and pEtN levels in cells that are blatantly senescent would result in a similar outcome. Our study does not address the mechanism underlying the effects of TP53 on GK gene expression and PCYT2 phosphorylation that we observe in senescent cells. Although suggested by our data, we lack definitive evidence that p16 has a dominant role in the establishment of senescence when we drive G3P accumulation. We identified the pro-senescent role of G3P and pEtN. The direct target remains to be established. Our in vivo analysis is preliminary and limited to the observation of increased levels of GK protein in senescent tissues. We did not determine whether those changes are associated with an equivalent effect on G3P levels.

## Methods

### Cell culture

WI38 fibroblasts (ATCC; CCL-75) were cultured in DMEM GLUTAMAX, high-glucose (Gibco) containing 10% fetal bovine serum (FBS) and 1× PenStrep (Thermo Fisher) at 37 °C with a 5% atmospheric concentration of O_2_ and CO_2_. The medium was changed every 2 d. Cells were split when they reached a confluency of 70–80%. Experiments were performed on cells at a population-doubling level inferior to 45 divisions (except for the RS model). WI38-ER:RASV12 fibroblasts were generated by retroviral transduction as previously described^20^. RAS OIS was induced by adding 400 nM 4-hydroxytamoxifen (4OHT) to the culture medium. The doxycycline-inducible oncogenic BRAFV600E retroviral construct was a gift from C. Mann (CEA, Gif-sur-Yvette, France). RAF OIS was induced with 100 ng ml^−1^ doxycycline. DDIS was triggered by etoposide treatment at a concentration of 20 µM for 2 d. Cells were washed and incubated with fresh medium without drug. RS was obtained through proliferative exhaustion. For the induction of quiescence, WI38 fibroblasts were cultured in DMEM containing 0.2% FBS. Primary human myoblasts (SkMC) were isolated from a skeletal muscle biopsy of a healthy donor (PromoCell, C-12530, lot 414Z025.11) and were purified with an immunomagnetic sorting system using CD56/NCAM magnetic beads (Miltenyi Biotec, 130-050-401) following the manufacturer’s specifications. The purified CD56-positive myoblasts were seeded in dishes coated with type I collagen (Sigma-Aldrich, C8919) and cultured in the proliferation medium (DMEM high glucose (Sigma, D6429), 20% FBS (Life Technologies, 10270106), 50 µg ml^−1^ gentamicin (Life Technologies, 15750037), 0.5% Ultroser G (PALL, 15950-017)) at 37 °C with 5% CO_2_. All experiments were conducted at Cumulative Population Doubling (CPD)-11 and CPD-29 to avoid replicative senescence and myoblasts were passaged at a cell confluency not exceeding 50% to avoid myogenic differentiation. Retroviral infections were performed as outlined for WI38 fibroblasts. For the indicated treatments of fibroblasts and myoblasts, cells were collected and processed at the indicated time points.

### Reagents for cell culture

We used 4OHT (H7904, Sigma) and doxycycline (D3447, Sigma) as described^20^. Etoposide (E1383, Sigma) was dissolved in 50 mM DMSO, aliquoted and stored at −20 °C. Before use, etoposide was added to fresh medium at a final concentration of 20 µM. Rapamycin (1292, Tocris) was dissolved in 100% ethanol at a 27.3 mM concentration, aliquoted and stored at −20 °C. Before use rapamycin was added to fresh medium from an intermediate 27.3 μM solution at a final concentration of 20 nM. DMOG (D3695, Sigma) was dissolved in water at a 150 mM concentration, aliquoted and stored at −20 °C. Before use, DMOG was added to fresh medium at a final concentration of 1 mM. 1-Thioglycerol (M1753, Sigma) was stored at 4 °C. Before use, 1-thioglycerol was added to fresh medium at a final concentration of 1 mM. Nutlin-3 (S1061, Selleckchem) was dissolved in DMSO at a 10 mM concentration, aliquoted and stored at −80 °C. Before use, Nutlin-3 was added to fresh medium at a final concentration of 10 μM.

### Viral transduction and transfection of siRNAs

Adenoviruses were transduced in cells incubated in an FBS-deprived medium for 3 h. Subsequently, cells were gently washed and incubated with fresh complete medium containing 4OHT, where required, to trigger ER:RASV12 induction. Supplementary Table 12 lists the adenoviruses used. For the transfection of siRNAs, cells plated in 6-cm dishes were transfected with siRNAs at a final concentration of 25 nM using the Transit-X2 Dynamic Delivery System (MIR6003, Mirus) according to the manufacturer’s instructions.

### Targeted LC–MS metabolomics analyses

For metabolomic analysis, the extraction solution was composed of 50% methanol, 30% acetonitrile (ACN) and 20% water. The volume of the extraction solution was adjusted to urea volume (1 ml per 1 × 10^6^ cells). After adding the extraction solution, samples were vortexed for 5 min at 4 °C and centrifuged at 16,000*g* for 15 min at 4 °C. The supernatants were collected and stored at −80 °C until analysis. LC–MS analyses were conducted on a QExactive Plus Orbitrap mass spectrometer equipped with an Ion Max source and a HESI II probe coupled to a Dionex UltiMate 3000 uHPLC system (Thermo). External mass calibration was performed using a standard calibration mixture every 7 d, as recommended by the manufacturer. The 5-μl samples were injected into a ZICpHILIC column (150 × 2.1 mm; internal diameter 5 μm) with a guard column (20 × 2.1 mm; internal diameter 5 μm) (Millipore) for LC separation. Buffer A was 20 mM ammonium carbonate and 0.1% ammonium hydroxide (pH 9.2) and buffer B was ACN. The chromatographic gradient was run at a flow rate of 0.200 μl min^−1^ as follows: 0–20 min, linear gradient from 80% to 20% of buffer B; 20–20.5 min, linear gradient from 20% to 80% of buffer B; and 20.5–28 min, 80% buffer B. The mass spectrometer was operated in full-scan, polarity-switching mode with the spray voltage set to 2.5 kV and the heated capillary held at 320 °C. The sheath gas flow was set to 20 units, the auxiliary gas flow to 5 units and the sweep gas flow to 0 units. The metabolites were detected across a mass range of 75–1,000 *m*/*z* at a resolution of 35,000 (at 200 *m*/*z*) with the automatic gain control target at 106 and the maximum injection time at 250 ms. Lock masses were used to ensure mass accuracy below 5 ppm. Data were acquired with Thermo Xcalibur software (Thermo). The peak areas of metabolites were determined using Thermo TraceFinder software (Thermo), identified by the exact mass of each singly charged ion and by the known retention time on the HPLC column.

Targeted metabolomics analyses were focused on the small polar compounds in central carbon metabolism. Established methods for sample extraction and LC–MS analyses using a pHILIC HPLC column for polar metabolite separation were used^68^. Additional details are in the supplementary files.

### Lipidomics

PL, TAG and DAG species in cells were analysed by Nano-Electrospray Ionization Tandem MS (Nano-ESI-MS/MS) with direct infusion of the lipid extract (Shotgun Lipidomics). A total of 5–10 × 10^6^ cells were homogenized in 500 µl of Milli-Q water using the Precellys 24 Homogenisator (Peqlab) at 6,500 r.p.m. for 30 s. The protein content of the homogenate was determined using bicinchoninic acid. Then, 35 µl (for PL analysis), 100 µl (for TAG analysis) or 20 µl (for DAG analysis) homogenate were diluted to 500 µl with Milli-Q water. For PL analysis, 1.875 ml methanol/chloroform 2:1 (*v*/*v*) and internal standards (125 pmol PC 17:0–20:4, 132 pmol PE 17:0–20:4, 118 pmol PI 17:0–20:4, 131 pmol PS 17:0–20:4 and 62 pmol PG 17:0–20:4; Avanti Polar Lipids) were added. For the analysis of TAG and DAG species, 1.875 ml chloroform/methanol/37% hydrochloric acid 5:10:0.15 (*v*/*v*/*v*) and 20 µl d5-TG Internal Standard Mixture I (for TAGs) or 30 µl each of d5-DG Internal Standard Mixtures I and II (for DAGs) (Avanti Polar Lipids) were used. Conditions of lipid extraction and Nano-ESI-MS/MS analysis have been previously described^69^. PC analysis was performed by scanning for precursors of *m*/*z* 184 Da at a collision energy (CE) of 35 eV. PE, PI, PS and PG measurements were conducted by scanning for neutral losses of *m*/*z* 141, 277, 185 and 189 Da with a CE of 25 eV. The value for the declustering potential was 100 V (ref. ^70^). Scanning was performed in a mass range of *m*/*z* 650–900 Da. TAG and DAG species were detected by scanning for the neutral losses of the ammonium adducts of distinct fatty acids: 271 (16:1), 273 (16:0), 295 (18:3), 297 (18:2), 299 (18:1), 301 (18:0), 321 (20:4) and 345 Da (22:6). For the analysis of TAG species, a mass range of *m*/*z* 750–1,100 Da was scanned with a CE of 40 eV, for DAG species the mass range was *m*/*z* 500–750 Da and the CE 25 eV (ref. ^70^). All scans were conducted in a positive-ion mode at a scan rate of 200 Da s^−1^. Mass spectra were processed by LipidView v.1.2 Software (SCIEX) to identify and quantify lipids. Endogenous lipid species were quantified by referring their peak areas to those of the internal standards. The calculated lipid amounts were normalized to the protein content of the cell homogenate. Additional details are in the supplementary files.

### Isotope labelling of Kennedy and glycerol pathway metabolites

To assess ethanolamine and glycerol uptake, we incubated cells with 100 µg ml^−1^ isotope-labelled Etn (MW + 2) (606294, Sigma) or 1.05 mM labelled glycerol (MW + 3) (489476, Sigma) for 1 h. For pulse–chase experiments, cells were washed with PBS twice after the pulse, then incubated with fresh medium containing an excess of non-labelled Etn (1 mM). Preparation of the extracts and measurement of labelled metabolite levels were performed as described above for ‘Targeted LC–MS metabolomics analyses’.

### Mitochondrial glycerol-3-phosphate dehydrogenase activity

The mitochondrial glycerol-3-phosphate dehydrogenase (EC 1.1.5.3) activity was estimated through the activity of G3P cytochrome c reductase spectrophotometrically measured on cell pellets at 37 °C (Cary 60 double wavelength spectrophotometer, Varian) according to previous work^71^. Levels of protein were estimated by the Bradford test.

### RNA purification reverse transcription and RT–qPCR

Total RNA was isolated using Trizol (QIAGEN) following the manufacturer’s instructions. RNA concentration was determined with a NanoDrop 2000 apparatus (Thermo Fisher Scientific). Reverse transcription was carried out on 100–150 ng of RNA using a SuperScript II RT kit (Invitrogen). Then, 4 μl 1:50 dilutions of the cDNAs were used in RT–qPCR reactions containing a SYBR Green PCR Master Mix (Bio-Rad). The reactions were carried on in a Stratagene MX3005P apparatus (Agilent Technologies). The thermal profile setup was 15 min at 95 °C followed by 40 cycles of alternating steps of 15 s at 95 °C and 30 s at 60 °C. Melt curve analysis was performed at the end of each run. The relative quantification of gene expression was performed using the 2^-ΔΔCT^ method, normalizing with RPS14 as a housekeeping transcript. Supplementary Table 12 lists the oligonucleotides used.

### RNA microarrays

Total RNA was purified using the QIAGEN RNeasy Plus kit according to the manufacturer’s instructions. Then, 100 ng RNA per sample were analysed using Affymetrix Human Transcriptome Arrays v.2.0, according to the manufacturer’s instructions.

### Cellular staining

SABG activity was assessed using the SABG Staining kit (Cell Signaling Technology) following the manufacturer’s instructions. Images were taken using an optical microscope and analysed using ImageJ software. Staining of lipid droplets was performed as previously described^72^. In brief, cells seeded in 24-well plates were fixed with 4% PFA for 15 min, then permeabilized and blocked for 45 min in 200 mM glycine, 3% BSA, 0.01% saponin and 1× PBS. After washing with PBS 1×, cells were incubated for 30 min with LipidTox Red (Thermo Fisher) diluted 1:200 in 0.1% BSA, 0.01% saponin and 1× PBS. All steps were carried out at room temperature. The coverslips were mounted using a mounting medium containing DAPI. Cells were imaged using a Spinning Disk microscope (Zeiss, Zen software) and analysed using ImageJ software.

### Immunohistochemistry

Mice tissues were fixed in 4% PFA, embedded in paraffin and sectioned at 4 μm. Sections were deparaffinized and hydrated before being boiled for 10 min in citrate buffer (pH6). Sections were blocked for 1 h in 0.1% Triton-X100, 0.1% Tween-20, 3% BSA and 5% goat serum in Tris-buffered saline (TBS) and then incubated with primary antibody overnight at 4 °C. Next, sections were incubated with biotinylated secondary antibodies and signal detected with the Vectastain Elite ABC kit (PK-6100; Vector Laboratories) and DAB chromogen system (DAKO). Pictures were acquired using a Nikon Eclipse Ti-S microscope (Nikon) using a ×10 and ×20 magnification.

### Immunoblotting

Cells were lysed in ice-cold lysis buffer (50 mM Tris-Cl, pH 7.4, 138 mM NaCl, 2.7 mM KCl, 5 mM EDTA, 20 mM NaF, 5% glycerol and 1% NP40) supplemented with protease and phosphatase inhibitor mixes (Roche). Protein concentrations were determined using the Bradford reagent (Bio-Rad). Equal amounts of extracts were resolved by 8, 10 or 12% SDS–PAGE and electro-transferred onto a polyvinylidene difluoride (PVDF) membrane (Amersham Biosciences). The preparation of 6% Phos-tag gels, migration and transfer were performed as described^73^. Blots were blocked in 1× TBS supplemented with 5% milk and subsequently incubated overnight at 4 °C in 3% BSA in 1× TBS supplemented with the primary antibodies diluted 1:1,000. After washing in TBS-Tween 0.1%, blots were incubated for 1 h at room temperature in TBS-milk 5% supplemented with HRP-conjugated secondary antibodies (Cell Signaling Technology; anti-mouse, 7076S; anti-rabbit, 7074S) diluted 1:5,000. After washing in TBS-Tween 0.1%, blots were developed with the Immobilon western chemiluminescence HRP substrate (Millipore). Images were acquired with a ChemiDoc Imager from Bio-Rad. Supplementary Table 12 lists the antibodies used.

### Mice

The generation and genotyping of PIK3CA^Adipo-CreER^ mice and LSL-KrasG12D^Ptf1a-Cre^ transgenic mice are described elsewhere^49,50^. The experiments were approved by the Direction Départementale des Services Véterinaires (Prefecture de Police, Paris; authorization no. 75-1313 and APAFIS, 34979). Mice were housed in a 12-h light–dark cycle and fed a standard chow diet (2018 Teklad Global, 18% protein rodent diets; 3.1 kcal g^−1^; Envigo). At the age of 6 weeks, *PIK3CA*^WT^ and *PIK3CA*^Adipo-CreER^ mice received a daily dose of tamoxifen (40 mg kg^−1^) for 5 d and were killed 6 weeks later. LSL-KrasG12D^Ptf1a-Cre^ mice were killed at 2 months of age together with the control mice.

### Analysis of metabolomics data

The data matrices were log-transformed for each batch and an analysis of variance (ANOVA) was performed to determine the statistical significance of metabolite differential accumulation. *P* values were corrected using the false discovery rate (FDR) approach and metabolites with a *q* value <0.05 were considered differentially accumulated. In total, 137 molecules were identified as differentially accumulated, at least in one sample and one experiment. Compound time profiles for each dataset were clustered independently using the WGCNA package^74^, with each sample being represented by the median of its replicates. The ‘soft threshold’ parameter was determined for each batch separately, with the choice of the lowest value leading to a high scale-free topology fit by applying the elbow method, as suggested by the tool authors. We set the parameters ‘minimum cluster size’, ‘deepSplit’ and ‘correlation threshold for cluster merging’ to 3, 3 and 0.60, respectively. We inspected signalling pathways enriched in each WGCNA module for each dataset by performing a hypergeometric test with pathways stored in the KEGG database^75^. We normalized the time profiles for the 46 metabolites identified in all batches with the ComBat tool^76^, using the initial, uninduced samples from each batch to estimate inter-batch effects. We performed an integrated PCA with the R package factoextra. We identified the specificities of the metabolic response elicited by distinct stressors by performing a sparse partial least squares discriminant analysis (sPLS-DA) using the mixOmics package^77^. We selected the 101 metabolites differentially accumulated in at least one time point for the CS fibroblast datasets (RAS OIS, RAF OIS, DDIS and RS) and determined the optimal number of components and features using the function perf. We integrated the RAS OIS metabolic response in fibroblasts and myoblasts by computing the overlap between each module identified for each dataset and by generating river plots connecting these modules with the R package networkD3 (https://cran.r-project.org/web/packages/networkD3/index.html).

### Analysis of transcriptomics data

We downloaded the raw Affymetrix HTA v.2.0 transcriptome data for the RAF-induced senescence and quiescence experiments from the Gene Expression Omnibus (GEO) database (BioProject PRJNA439263, accession codes GSE143248 and GSE112084 (ref. ^20^)). Oncogenic RAS, RAF, DDIS and RS transcriptomes were measured as described above. For each dataset, we normalized expression levels using the robust multichip average tool provided by the oligonucleotide R package^77^ and performed a surrogate variable analysis with the sva^78^ and limma^79^ R packages.

We eliminated internal Affymetrix control probes and annotated the remaining probes using the hta20sttranscriptcluster.db R package. We removed lowly expressed probes, specifically the bottom 40% of genes, considering all samples in a dataset. We applied ANOVA FDR and selected genes with a *q* value lower than 0.05 and 1.5 × log_2_FC for each dataset.

The hierarchical clustering performed on the transcriptome data is similar to the one applied to the metabolome. We clustered genes from each experiment with WGCNA^74^, using their replicates median value for each sample. As mentioned above, we individually determined the ‘soft threshold’ for each dataset. The parameters ‘minimum cluster size’ and ‘deepSplit’ were set to 100 and 3, respectively. The ‘correlation threshold for cluster merging’ parameter was optimized independently for each inducer and set to 0.7 for RAS OIS (fibroblasts), 0.75 for RAS OIS (myoblasts), 0.75 for RAF OIS, 0.8 for DDIS, 0.8 FOR RS and 0.9 for quiescence. Furthermore, we integrated the fibroblast and myoblast RAS OIS response by computing the overlap between each transcriptional cluster and by generating river plots using the networkD3 R package, in a similar procedure as that performed for the metabolome.

We investigated signalling pathways enriched for differential genes in each dataset by performing an over-representation analysis using the R package v.7.5.1.9001 Molecular Signature Database (MSigDB) (https://igordot.github.io/msigdbr/) and clusterProfiler^80^, combined with the MSigDB hallmark gene sets^81,82^. For each dataset, we analysed each coexpression module identified by WGCNA separately. As described above, we also used river plots produced by the R package networkD3 to integrate the gene expression dynamics from both experiments. To assess DMOG and rapamycin-induced changes in SASP gene expression of RAS OIS or DDIS cells, we ranked differentially expressed genes according to their fold change, comparing the samples at the end of each time course (day 7 for RAS OIS + DMOG and day 14 for DDIS + rapamycin) and performed GSEA using a comprehensive, published SASP Atlas as a ref. ^22^.

### Batch-correction methods benchmark

We evaluated five batch-correction (BC) methods reported in the literature to integrate the data from different senescence inducers. Namely, the methods were quantile normalization, implemented by the oligonucleotide R package^77^, a BC technique using the quality control samples from each batch as a reference, as described elsewhere^83^, a third approach based on the average of all samples in a given batch as a reference for normalization^84^, a strategy using samples corresponding to biological replicates in each batch as reference for BC (cells before CS or quiescence induction) and the ComBat tool, which infers the parameters of a linear model for BC using a Bayesian approach^76^. The approaches consisting of using a set of samples average as the normalization reference follow the general form given by equation (1).

1 $$\begin{array}{c}{X}_{{\mathrm{p}},{\mathrm{s}},{\mathrm{b}}}^{{\prime} }={X}_{{\mathrm{p}},{\mathrm{s}},{\mathrm{b}}}\frac{{R}_{\mathrm{p}}}{{C}_{{\mathrm{p}},{\mathrm{s}},{\mathrm{b}}}}\\ with\,{R}_{\mathrm{p}}=\mathop{\mathrm{average}}\limits_{\forall i,\,j}({X}_{p,i,\,j})\end{array}$$

Where *X*′_p,s,b_ and *X*_p,s,b_ are respectively the normalized and raw intensity of peak p at sample s in batch b; *R*_p_ is a scaling factor computed by the average of all detected values for a peak p in all samples in all batches and *C*_p,s,b_ is the correction factor computed on the set of reference samples. The following equations give its computation for each set of reference samples.

2 $$\begin{array}{c}{\mathrm{Quality}}\,{\mathrm{control}}\,{\mathrm{samples}}:{C}_{\mathrm{p},\mathrm{s},\mathrm{b}}=\mathop{\mathrm{average}}\limits_{i\in QC(b)}({X}_{{\mathrm{p}},{\mathrm{i}},{\mathrm{b}}})\\ \mathrm{Uninduced}\,\mathrm{samples}:{C}_{{\mathrm{p}},{\mathrm{s}},{\mathrm{b}}}=\mathop{\mathrm{average}}\limits_{i\in QC=D00(b)}({X}_{{\mathrm{p}},{\mathrm{i}},{\mathrm{b}}})\\ {\mathrm{All}}\,{\mathrm{batch}}\,{\mathrm{samples}}={C}_{{\mathrm{p}},{\mathrm{s}},{\mathrm{p}}}=\mathop{\mathrm{average}}\limits_{\forall i\in b}({X}_{{\mathrm{p}},{\mathrm{i}},{\mathrm{b}}})\end{array}$$

We compared those methods based on the values obtained by the computation of three metrics: relative s.d. (r.s.d.), repeatability and the Bhattacharyya distance.

The r.s.d. consists of the ratio between the s.d. (σ) and the average intensity values (*µ*) measured for each peak p. This value is computed for each sample s over all batches as determined by the following equation^84^.

3 $$\mathrm{r.s.d.}=\frac{{\sigma }_{{\mathrm{p}},{\mathrm{s}}}}{{\mu }_{{\mathrm{p}},{\mathrm{s}}}}$$

Repeatability measures the fraction of the variance between replicates of the same sample s over all batches^85^. Its computation is performed for each measured peak p, dividing the variance between the averages of all replicates for sample s by the variance of the intensity observed in all replicates within the same sample, as shown in equation (5). High repeatability is attained by samples sparsely distributed, with replicates densely clustered. As the variance for replicates within a sample approaches (or surpasses) the variance between samples, this quantity decreases.

4 $$\mathrm{Repeatability}=\frac{{\sigma }_{\mathrm{between};\,{\mathrm{p}},{\mathrm{s}}}^{2}}{{\sigma }_{\mathrm{between};\,{\mathrm{p}},{\mathrm{s}}}^{2}+{\sigma }_{\mathrm{within};\,{\mathrm{p}},{\mathrm{s}}}^{2}}\approx \frac{{\sigma }_{\mathrm{biol};\,{\mathrm{p}},{\mathrm{s}}}^{2}}{{\sigma }_{\mathrm{total};\,{\mathrm{p}},{\mathrm{s}}}^{2}}$$

The Bhattacharyya distance (DB) is an extension of the Malahanobis distance. The Malahanobis distance measures the distance between two sets of points, normalized by their covariance. Therefore, tighter clusters will lead to a higher Malahanobis distance for the same distance between their centre of mass. The DB extends this concept by introducing a factor accounting for a distinct distribution in both sets. This metric was calculated using the fpc R package and is given by^85^:

5 $$\mathrm{DB}=\frac{1}{8}{(\,{\mu }_{1;{\mathrm{s}}}-{\mu }_{2;{\mathrm{s}}})}^{T}{\sum }^{(-1)}(\,{\mu }_{1;{\mathrm{s}}}-{\mu }_{2;{\mathrm{s}}})+\frac{1}{2}\,\mathrm{ln}\left({\det }\frac{{\sum }_{{\mathrm{s}}}}{{\det }{\sum }_{1;{\mathrm{s}}}{\det }{\sum }_{2;{\mathrm{s}}}}\right)$$

Where *µ*_b;s_ corresponds to the centre of mass of sample s for batch b, *Σ*_b;s_ is the covariance matrix for samples replicates in batch b and *Σ*_s_ is the covariance matrix for sample s in all batches.

### Integration of transcriptomics and metabolomics data

Aiming to identify potential non-linear molecular interactions, we computed the Spearman correlation for each gene–metabolite pair in each dataset in an approach inspired by previous work^86^. We calculated the overlap of high correlations (absolute value higher than 0.5) in all datasets with the R package Vennerable. We visualized these overlapping correlations to build gene–metabolite networks with the R packages ComplexHeatmap^87^, CyREST^88^, RCy3 (ref. ^89^) and Cytoscape software^90^. Gene Ontology analysis of G3P-correlating genes was performed on targets correlating positively or negatively with G3P in at least three out of four senescence inducers (quiescence condition excluded).

The analysis was performed on ShinyGO, using the hallmark MSigDB. Curated Reactome analysis was performed on the G3P-correlating gene either positively or negatively. The analysis was performed on ShinyGO, using the Curated Reactome database.

### Statistics

Quantitative data in graphs are presented as the mean ± s.d. unless indicated otherwise in the figure legends. Statistical tests used in this study include unpaired two-sided Student’s *t*-test and one-way ANOVA as indicated in the figure legends. Significant differences are reported as *P* values in the figure legends and the exact values are indicated where appropriate. No statistical method was used to predetermine the sample size, but our sample sizes are similar to those reported in a previous publication^20^. Data derived from time-series Affymetrix microarrays were highly reproducible. All transcriptomics were performed in biological duplicate (WI38 Ras ± DMOG and WI38 etoposide ± rapamycin). RT–qPCR on adenovirus-infected cells was performed in biological triplicate. Metabolomics was performed on biological triplicates for each time point and condition and lipidomics were performed on a minimum of four biological replicates for each condition. Data distribution was assumed to be normal but this was not formally tested. Biological materials (cells and mice) were randomized before experiments. Data collection and analysis were not performed blind to the conditions of the experiments. Outliers were identified and excluded by the ROUT method (default setting) on GraphPad Prism.

### Reporting summary

Further information on research design is available in the Nature Portfolio Reporting Summary linked to this article.

### Supplementary information

Supplementary Information Supplementary methods.

Reporting Summary

Supplementary Table 1 Microarray gene expression data in RAS OIS, RAF OIS, DDIS, RS and quiescence.

Supplementary Table 2 Pathway enrichment analysis of transcriptomes of RAS OIS, RAF OIS, DDIS, RS and quiescence.

Supplementary Table 3 Metabolite levels in RAS OIS, RAF OIS, DDIS, RS and quiescence.

Supplementary Table 4 Pathway enrichment analysis of metabolomes of RAS OIS, RAF OIS, DDIS, RS and quiescence.

Supplementary Table 5 Affymetrix microarray gene expression data in RAS OIS ± DMOG and DDIS ± rapamycin.

Supplementary Table 6 Pathway enrichment analysis of transcriptomes of RAS OIS ± DMOG and DDIS ± rapamycin.

Supplementary Table 7 List of genes overlapping between RAS OIS + DMOG and DDIS + rapamycin as shown in the river plot (related to Extended Data Fig. 5e).

Supplementary Table 8 GSEA for the hallmark SASP for the RAS OIS ± DMOG and DDIS ± rapamycin.

Supplementary Table 9 Gene–metabolite correlations in RAS OIS, RAF OIS, DDIS, RS and quiescence.

Supplementary Table 10 Network metrics for overlapping gene–metabolite correlations in RAS OIS, RAF OIS, DDIS, RS and quiescence.

Supplementary Table 11 Gene Ontology analysis of genes correlating positively or negatively with G3P.

Supplementary Table 12 Lists of vectors, antibodies, primers and siRNA used in this study.

Supplementary data 1 Supplementary lipidomics raw data.

Supplementary data 2 Supplementary metabolomics raw data.

### Source data

Source Data Fig. 2 Statistical source data.

Source Data Fig. 3 Statistical source data.

Source Data Fig. 4 Statistical source data.

Source Data Fig. 4 Unprocessed western blots.

Source Data Fig. 5 Statistical source data.

Source Data Fig. 6 Statistical source data.

Source Data Fig. 6 Unprocessed western blots.

Source Data Fig. 7 Statistical source data.

Source Data Fig. 8 Statistical source data.

Source Data Fig. 8 Unprocessed western blots.

Source Data Extended Data Fig. 3 Statistical source data.

Source Data Extended Data Fig. 4 Statistical source data.

Source Data Extended Data Fig. 6 Statistical source data.

Source Data Extended Data Fig. 6 Unprocessed western blots.

Source Data Extended Data Fig. 7 Statistical source data.

Source Data Extended Data Fig. 7 Unprocessed western blots.

Source Data Extended Data Fig. 8 Statistical source data.

Source Data Extended Data Fig. 8 Unprocessed western blots.

Source Data Extended Data Fig. 9 Statistical source data.

Source Data Extended Data Fig. 9 Unprocessed western blots.

Source Data Extended Data Fig. 10 Statistical source data.

Source Data Extended Data Fig. 10 Unprocessed western blots.

## Data Availability

Metabolomics data that include four senescence onset and quiescence models, RAS OIS cells treated with DMOG or shPCYT2, OE G3PP, PCYT2 or ETNPPL and etoposide-induced cells treated with rapamycin are available in supplementary tables and supplementary data and have also been deposited to the EMBL-EBI MetaboLights database^91^ with identifier MTBLS7118. The complete dataset can be accessed at https://www.ebi.ac.uk/metabolights/MTBLS7118. Lipidomics raw data have been provided in Supplementary Data. Transcriptome raw data for RAS-induced senescence, DNA damage and RS will be published in GEO under accession code GSE248824. Previously published transcriptome data (RAF-induced senescence and quiescence) are hosted on GEO under accession codes GSE143248 and GSE112084. Files of source data are provided for quantifications, statistical analysis and uncropped western blots. Source data are provided with this paper.

## Code availability

The scripts used to preprocess raw transcriptomic data, perform statistical analyses, visualize and integrate metabolomics and transcriptomic data are hosted on Zenodo (https://zenodo.org/records/8199751) as both Rmardkdown and html files. Where applicable, we uploaded the necessary, processed data to generate the figures in this manuscript.
