# Supplementary material for: A homoeostatic switch causing glycerol-3-phosphate and phosphoethanolamine accumulation triggers senescence by rewiring lipid metabolism
Source: Nat Metab. 2024 Feb 19;6(2):323–42. doi: 10.1038/s42255-023-00972-y (PMC10896726; doi:10.1038/s42255-023-00972-y)
Supplement: Supplementary file 1 — Supplementary methods. [file 42255_2023_972_MOESM1_ESM.pdf]

nature metabolism

Article https://doi.org/10.1038/s42255-023-00972-y

A homoeostatic switch causing glycerol-3-phosphate and phosphoethanolamine accumulation triggers senescence by rewiring lipid metabolism

In the format provided by the
authors and unedited

Targeted LC-MS metabolomics analyses:

As a part of the routine analytical pipeline, recommendations of the metabolomics Quality Assurance and quality Control Consortium (mQACC) were applied: the routine quality controls include regular equipment maintenance (Thermo),the use of standard operating procedures for sample extraction, storage and analyses. General practices also include weekly test runs to assure system stability and quality of runs. Several QCs were used (1) pooled interstudy QC, (2) process and extraction blanks, (3) system stability blanks, (4) solvents blanks, (5) long-term reference standard inter-laboratory QC mix to ensure system stability and (6) the samples wereblinded and loaded in randomized order. The analyses of pooled samples QC showedno significant difference in metabolites levels between QCs.

Direct Infusion Workflow

Created by https://lipidomicstandards.org, version v2.2.5

Overall study design

Title of the study	Analysis of glycerolipid and glycerophospholipid species in WI38 fibroblasts exposed to etoposide-mediated DNA damage-induced senescence and replicative senescence induced by proliferative exhaustion		
Document creation date	08/25/2023	Corresponding Email	susanne.brodesser@uk-koeln.de
Principle investigator	Susanne Brodesser, CECAD Lipidomics/Metabolomics Facility	Is the workflow targeted or untargeted?	Targeted
Institution	University of Cologne, Germany	Clinical	No

Lipid extraction

Extraction method	2-phase system	2-phase system	Bligh&Dyer
pH adjustment	None	Were internal standards added prior extraction?	Yes

Analytical platform

MS type	QTrap	Mass window for precursor ion isolation (in Da total isolation window)	0.7
MS vendor	SCIEX	Mass resolution for detected ion at MS2	Low resolution
Ion source	ESI	Resolution at MS2	Unit
Direct type	Chip	Was/Were additional dimension/techniques used	No
MS Level	MS2		

Quality control

Blanks	Yes	Quality control	No
Type of Blanks	Extraction blank, Injection blank		

Method qualification and validation

Method validation	No

Reporting

Are reported raw data uploaded into repository?	Available on request	Raw data upload	Available on request
Are metadata available?	Available on request	Additional comments	Lipid analyses were performed according to Özbalci C, Sachsenheimer T, Brügger B. (2013) Quantitative Analysis of Cellular Lipids by Nano-Electrospray Ionization Mass Spectrometry. Methods Mol Biol 1033:3-20.

Sample Descriptions

Senescence metabolism / Human / Cells

Provided information	-	Additives	None
Temperature handling original sample	4-8 °C	Were samples stored under inert gas?	No
Instant sample preparation	No	Additional preservation methods	No
Storage temperature	-80 °C	Biobank samples	No

Lipid Class Descriptions

1) DG[M+NH4]+ / Lipid identification

Lipid class	DG	Did you presume assumptions for identification?	Yes
MS Level for identification	MS2	Which assumptions were presumed?	Use of target method in LipidView software
Identification level	Molecular species level	Check isomer overlap	Yes
Polarity mode	Positive	Additional dimension/techniques	-
Type of positive (precursor)ion	[M+NH4]+	Lipid Identification Software	LipidView
Fragments for identification	Fragment name: -FA(+NH4)	Data manipulation	Smoothing
Isotope correction at MS2	No	Nomenclature for intact lipid molecule	Yes
MS2 verified by standard	Yes	Nomenclature for fragment ions	Yes
Background check at MS2	Yes	Further identification remarks	DG species were detected by scanning for the neutral losses of the ammonium adducts of distinct fatty acids: 271 (16:1), 273 (16:0), 297 (18:2), 299 (18:1), 301 (18:0), and 321 Da (20:4).

1) DG[M+NH4]+ / Lipid quantification

Quantitative	Yes	Limit of quantification	S/N ratio
MS Level for quantification	MS1	Normalization to reference	Yes
Internal lipid standard(s) MS1		Lipid Quantification Software	LipidView

Internal standard	Endogenous subclass
D5 DG 16:1_16:1	DG 16:1_XX:X
D5 DG 16:0_16:0	DG 16:0_XX:X
D5 DG 18:2_18:2	DG 18:2_XX:X
D5 DG 18:1_18:1	DG 18:1_XX:X
D5 DG 18:0_18:0	DG 18:0_XX:X
D5 DG 20:4_20:4	DG 20:4_XX:X

Type of quantification	Internal standard amount	Batch correction	No
Response correction	No	Further quantification remarks	-
Type I isotope correction	Yes		

2) TG[M+NH4]+ / Lipid identification

Lipid class	TG	Did you presume assumptions for identification?	Yes
MS Level for identification	MS2	Which assumptions were presumed?	Use of target method in LipidView software
Identification level	Species level	Check isomer overlap	Yes
Polarity mode	Positive	Additional dimension/techniques	-
Type of positive (precursor)ion	[M+NH4]+	Lipid Identification Software	LipidView
Fragments for identification		Data manipulation	Smoothing

Fragment name
-FA(+NH4)

Isotope correction at MS2	No	Nomenclature for intact lipid molecule	Yes
MS2 verified by standard	Yes	Nomenclature for fragment ions	Yes
Background check at MS2	Yes	Further identification remarks	TG species were detected by scanning for the neutral losses of the ammonium adducts of distinct fatty acids: 273 (16:0), 295 (18:3), 297 (18:2), 299 (18:1), 301 (18:0), 321 (20:4), and 345 Da (22:6).

2) TG[M+NH4]+ / Lipid quantification

Quantitative	Yes	Limit of quantification	S/N ratio
MS Level for quantification	MS1	Normalization to reference	Yes
Internal lipid standard(s) MS1		Lipid Quantification Software	LipidView

Internal standard	Endogenous subclass
D5 TG 16:0_18:0_16:0	TG 16:0_XX:X, TG 18:0_XX:X
D5 TG 15:0_18:1_15:0	TG 18:1_XX:X
D5 TG 20:4_18:2_20:4	TG 18:2_XX:X, TG 20:4_XX:X
D5 TG 20:2_18:3_20:2	TG 18:3_XX:X
D5 TG 20:5_22:6_20:5	TG 22:6_XX:X

Type of quantification	Internal standard amount	Batch correction	No
Response correction	No	Further quantification remarks	-
Type I isotope correction	Yes		

3) PC[M+H]+ / Lipid identification

Lipid class	PC	Did you presume assumptions for identification?	Yes
MS Level for identification	MS2	Which assumptions were presumed?	Use of target method in LipidView
Identification level	Species level	Check isomer overlap	Yes
Polarity mode	Positive	Additional dimension/techniques	-
Type of positive (precursor)ion	[M+H]+	Lipid Identification Software	LipidView
Fragments for identification		Data manipulation	Smoothing

Fragment name
HG(PC,184)

Isotope correction at MS2	No	Nomenclature for intact lipid molecule	Yes
MS2 verified by standard	Yes	Nomenclature for fragment ions	Yes
Background check at MS2	Yes	Further identification remarks	PC detection was performed by scanning for precursors of m/z 184 Da.

3) PC[M+H]+ / Lipid quantification

Quantitative	Yes	Limit of quantification	S/N ratio
MS Level for quantification	MS1	Normalization to reference	Yes
Internal lipid standard(s) MS1		Lipid Quantification Software	LipidView

Internal standard	Endogenous subclass
PC 17:0_20:4	PC XX:X

Type of quantification	Internal standard amount	Batch correction	No
Response correction	No	Further quantification remarks	-
Type I isotope correction	Yes		

4) PE[M+H]+ / Lipid identification

Lipid class	PE	Did you presume assumptions for identification?	Yes
MS Level for identification	MS2	Which assumptions were presumed?	Use of target method in LipidView software
Identification level	Species level	Check isomer overlap	Yes
Polarity mode	Positive	Additional dimension/techniques	-
Type of positive (precursor)ion	[M+H]+	Lipid Identification Software	LipidView
Fragments for identification	Fragment name: -HG(PE,141)	Data manipulation	Smoothing
Isotope correction at MS2	No	Nomenclature for intact lipid molecule	Yes
MS2 verified by standard	Yes	Nomenclature for fragment ions	Yes
Background check at MS2	Yes	Further identification remarks	PE detection was conducted by scanning for neutral losses of m/z 141 Da.

4) PE[M+H]+ / Lipid quantification

Quantitative	Yes	Limit of quantification	S/N ratio
MS Level for quantification	MS1	Normalization to reference	Yes
Internal lipid standard(s) MS1		Lipid Quantification Software	LipidView
Type of quantification	Internal standard amount	Batch correction	No
Response correction	No	Further quantification remarks	-
Type I isotope correction	Yes		

Internal standard	Endogenous subclass
PE 17:0_20:4	PE XX:X

5) PI[M+NH4]+ / Lipid identification

Lipid class	PI	Did you presume assumptions for identification?	Yes
MS Level for identification	MS2	Which assumptions were presumed?	Use of target method in LipidView software
Identification level	Species level	Check isomer overlap	Yes
Polarity mode	Positive	Additional dimension/techniques	-
Type of positive (precursor)ion	[M+NH4]+	Lipid Identification Software	LipidView
Fragments for identification	Fragment name: -HG(PI,277)	Data manipulation	Smoothing
Isotope correction at MS2	No	Nomenclature for intact lipid molecule	Yes
MS2 verified by standard	Yes	Nomenclature for fragment ions	Yes
Background check at MS2	Yes	Further identification remarks	PI detection was conducted by scanning for neutral losses of m/z 277 Da.

5) PI[M+NH4]+ / Lipid quantification

Quantitative	Yes	Limit of quantification	S/N ratio
MS Level for quantification	MS1	Normalization to reference	Yes
Internal lipid standard(s) MS1		Lipid Quantification Software	LipidView

Internal standard	Endogenous subclass
PI 17:0_20:4	PI XX:X

Type of quantification	Internal standard amount	Batch correction	No
Response correction	No	Further quantification remarks	-
Type I isotope correction	Yes		

6) PS[M+H]+ / Lipid identification

Lipid class	PS	Did you presume assumptions for identification?	Yes
MS Level for identification	MS2	Which assumptions were presumed?	Use of target method in LipidView
Identification level	Species level	Check isomer overlap	Yes
Polarity mode	Positive	Additional dimension/techniques	-
Type of positive (precursor)ion	[M+H]+	Lipid Identification Software	LipidView
Fragments for identification		Data manipulation	Smoothing

Fragment name
-HG(PS,185)

Isotope correction at MS2	No	Nomenclature for intact lipid molecule	Yes
MS2 verified by standard	Yes	Nomenclature for fragment ions	Yes
Background check at MS2	Yes	Further identification remarks	PS detection was conducted by scanning for neutral losses of m/z 185 Da.

6) PS[M+H]+ / Lipid quantification

Quantitative	Yes	Limit of quantification	S/N ratio
MS Level for quantification	MS1	Normalization to reference	Yes
Internal lipid standard(s) MS1		Lipid Quantification Software	LipidView

Internal standard	Endogenous subclass
PS 17:0_20:4	PS XX:X

Type of quantification	Internal standard amount	Batch correction	No
Response correction	No	Further quantification remarks	-
Type I isotope correction	Yes		

7) PG[M+NH4]+ / Lipid identification

Lipid class	PG	Did you presume assumptions for identification?	Yes
MS Level for identification	MS2	Which assumptions were presumed?	Use of target method in LipidView software
Identification level	Species level	Check isomer overlap	Yes
Polarity mode	Positive	Additional dimension/techniques	-
Type of positive (precursor)ion	[M+NH4]+	Lipid Identification Software	LipidView
Fragments for identification		Data manipulation	Smoothing

Fragment name
-HG(PG,189)

Isotope correction at MS2	No	Nomenclature for intact lipid molecule	Yes
MS2 verified by standard	Yes	Nomenclature for fragment ions	Yes
Background check at MS2	Yes	Further identification remarks	PG detection was conducted by scanning for neutral losses of m/z 189 Da.

7) PG[M+NH4]+ / Lipid quantification

Quantitative	Yes	Limit of quantification	S/N ratio
MS Level for quantification	MS1	Normalization to reference	Yes
Internal lipid standard(s) MS1		Lipid Quantification Software	LipidView

Internal standard	Endogenous subclass
PG 17:0_20:4	PG XX:X

Type of quantification	Internal standard amount	Batch correction	No
Response correction	No	Further quantification remarks	-
Type I isotope correction	Yes		
