# Supplementary material for: A homoeostatic switch causing glycerol-3-phosphate and phosphoethanolamine accumulation triggers senescence by rewiring lipid metabolism
Source: Nat Metab. 2024 Feb 19;6(2):323–42. doi: 10.1038/s42255-023-00972-y (PMC10896726; doi:10.1038/s42255-023-00972-y)
Supplement: Supplementary file 2 — Reporting Summary [file 42255_2023_972_MOESM2_ESM.pdf]

nature portfolio

Corresponding author(s): Mario Pende

Last updated by author(s): 21/12/2023

Reporting Summary

Nature Portfolio wishes to improve the reproducibility of the work that we publish. This form provides structure for consistency and transparency in reporting. For further information on Nature Portfolio policies, see our Editorial Policies and the Editorial Policy Checklist.

Statistics

For all statistical analyses, confirm that the following items are present in the figure legend, table legend, main text, or Methods section.

n/a	Confirmed	
☐	☒	The exact sample size (*n*) for each experimental group/condition, given as a discrete number and unit of measurement
☐	☒	A statement on whether measurements were taken from distinct samples or whether the same sample was measured repeatedly
☐	☒	The statistical test(s) used AND whether they are one- or two-sided<br>*Only common tests should be described solely by name; describe more complex techniques in the Methods section.*
☐	☒	A description of all covariates tested
☐	☒	A description of any assumptions or corrections, such as tests of normality and adjustment for multiple comparisons
☐	☒	A full description of the statistical parameters including central tendency (e.g. means) or other basic estimates (e.g. regression coefficient) AND variation (e.g. standard deviation) or associated estimates of uncertainty (e.g. confidence intervals)
☐	☒	For null hypothesis testing, the test statistic (e.g. *F*, *t*, *r*) with confidence intervals, effect sizes, degrees of freedom and *P* value noted<br>*Give P values as exact values whenever suitable.*
☒	☐	For Bayesian analysis, information on the choice of priors and Markov chain Monte Carlo settings
☒	☐	For hierarchical and complex designs, identification of the appropriate level for tests and full reporting of outcomes
☒	☐	Estimates of effect sizes (e.g. Cohen's *d*, Pearson's *r*), indicating how they were calculated

Our web collection on statistics for biologists contains articles on many of the points above.

Software and code

Policy information about availability of computer code

Data collection

ZEN software (version 2.1) for ZEISS microscope (Spinnning Disk microscope). For Lipidomics data collection Nano-Electrospray Ionization Tandem Mass Spectrometry (Nano-ESI-MS/MS) with direct infusion of the lipid extract (Shotgun Lipidomics) was used, and mass spectra were processed by the LipidView Software (SCIEX) (version 1.2). For transcriptomics, Affymetrix Human Transcriptome Arrays 2.0 was used. For metabolomics analyses, data were collected using LC/MS a on a QExactive Plus Orbitrap mass spectrometer equipped with an Ion Max source and a HESI II probe coupled to a Dionex UltiMate 3000 uHPLC system (ThermoFischer). The peak areas of metabolites were determined using Thermo TraceFinder software (ThermoFischer, version 3.3.350.0).

Data analysis

We analyzed metabolome and transcriptome data using the following tools:
R version 4.2.1
Cytoscape version 3.9.0
R packages:
gplots v3.1.3
RCy3 v2.16.0
igraph v1.4.1
stringr v1.5.0
Vennerable v3.1.0.9000
htmlwidgets v1.6.1
networkD3 v0.4
dplyr v1.1.0
enrichplot v1.16.2
msigdbr v7.5.1

clusterProfiler v4.4.4
sva v3.44.0
BiocParallel v1.32.5
genefilter v1.78.0
mgcv v1.8-40
nlme v3.1-159
limma v3.54.2
preprocessCore v1.58.0
pd.hta.2.0 v3.12.2
DBI v1.1.3oligo v1.60.0
oligoClasses v1.58.0
RSQLite v2.3.0
Biostrings v2.66.0
GenomeInfoDb v1.34.9
XVector v0.38.0
hta20transcriptcluster.db v8.8.0
org.Hs.eg.db v3.15.0
AnnotationDbi v1.60.2
IRanges v2.32.0
S4Vectors v0.36.2
Biobase v2.58.0
BiocGenerics v0.44.0
fpc v2.2-10intervals v0.15.2
KEGGREST v1.38.0
WGCNA v1.72-1
fastcluster v1.2.3
dynamicTreeCut v1.63-1
gridExtra v2.3
reshape2 v1.4.4
ggrepel v0.9.3
circlize v0.4.15
factoextra v1.0.7
ggplot2 v3.4.1
ComplexHeatmap v2.14.0
Microscopy images were analyzed using Image J (v 1.53t).
All code in R is available upon request and Rmmd and html files are uploaded to Zenodo.
Histograms were generated by: GraphPad Prism 9.5.0(730). Two-sided student T-tests were performed on GraphPad Prism 9.5.0(730) and Excel. Heatmaps for fig.5D,F,G fig.7E were generated on GraphPad Prism 9.5.0(730). Heatmaps for ED.fig.5 F,G ED.fig.7D ED.fig.8A,B ED.fig.9B,C were generated on www.metaboanalyst.ca website

For manuscripts utilizing custom algorithms or software that are central to the research but not yet described in published literature, software must be made available to editors and reviewers. We strongly encourage code deposition in a community repository (e.g. GitHub). See the Nature Portfolio guidelines for submitting code & software for further information.

Data

Policy information about availability of data

All manuscripts must include a data availability statement. This statement should provide the following information, where applicable:

- Accession codes, unique identifiers, or web links for publicly available datasets
- A description of any restrictions on data availability
- For clinical datasets or third party data, please ensure that the statement adheres to our policy

Metabolomics data which includes four senescence onset and quiescence models; RAS-OIS cells treated with DMOG or shPCYT2, overexpressing G3PP, PCYT2 or ETNPPL and etoposide-induced cells treated with Rapamycin have been deposited to the EMBL-EBI MetaboLights database with the identifier MTBLS7118. The complete dataset can be accessed here https://www.ebi.ac.uk/metabolights/MTBLS7118. Raw data of lipidomics and metabolomics are also available in Supplementary tables and Supplementary data.
Transcriptome raw data for RAS-induced senescence, DNA damage and replicative senescence will be published in the Gene Expression Onmibus (GEO) database (GSE248824). Previously published transcriptome data (RAF-induced senescence and quiescence) are hosted on the GEO website under accession codes GSE143248 and GSE112084.

Human research participants

Policy information about studies involving human research participants and Sex and Gender in Research.

Reporting on sex and gender	Does not apply.
Population characteristics	Does not apply.
Recruitment	Does not apply.
Ethics oversight	Does not apply.

Note that full information on the approval of the study protocol must also be provided in the manuscript.

Field-specific reporting

Please select the one below that is the best fit for your research. If you are not sure, read the appropriate sections before making your selection.

☒ Life sciences ☐ Behavioural & social sciences ☐ Ecological, evolutionary & environmental sciences

For a reference copy of the document with all sections, see nature.com/documents/nr-reporting-summary-flat.pdf

Life sciences study design

All studies must disclose on these points even when the disclosure is negative.

Sample size	No statistical method was used to predetermine sample size. The minimum number of experiments selected to perform statistical analyses is 3. Experiments were independently repeated at least twice as indicated, and mean and the standard deviation were calculated.<br>Samples sizes were determined based also on previously published papers using the same paradigms:<br>-Georgilis A, Klotz S, Hanley CJ, et al. PTBP1-Mediated Alternative Splicing Regulates the Inflammatory Secretome and the Pro-tumorigenic Effects of Senescent Cells. Cancer Cell. 2018;34(1):85-102.e9. doi:10.1016/j.ccell.2018.06.007<br>-Martínez-Zamudio RI, Roux PF, de Freitas JANLF, et al. AP-1 imprints a reversible transcriptional programme of senescent cells [published correction appears in Nat Cell Biol. 2020 Sep 16;:]. Nat Cell Biol. 2020;22(7):842-855. doi:10.1038/s41556-020-0529-5
Data exclusions	Outliers were identified and excluded by the ROUT method (default setting) on GraphPad Prism. Exclusion criteria were not pre-established.
Replication	Experiments were carried out in biological replicates as indicated in the results part (text and figure legends). The reproducibility of the experimental findings were verified by performing additional independent experiments. All attempts at replication were confirmed to be successful.
Randomization	Biological materials (cells and mice) were randomized before experiments.
Blinding	Experiments were performed in non-blinded manner because cells needed to be carefully documented by investigators, so blinding was not always possible during experimental setup, such as in SABG assays. Key observations (fig.1, fig.4F,H and fig.8E, fig.5C) were performed and analysed by different operators. No blinding was involved in other experiments such as Metabolomics, lipidomics, qPCR assays, as machine-based readouts are not subject to investigator bias.

Behavioural & social sciences study design

All studies must disclose on these points even when the disclosure is negative.

Study description	Does not apply.
Research sample	Does not apply.
Sampling strategy	Does not apply.
Data collection	Does not apply.
Timing	Does not apply.
Data exclusions	Does not apply.
Non-participation	Does not apply.
Randomization	Does not apply.

Ecological, evolutionary & environmental sciences study design

All studies must disclose on these points even when the disclosure is negative.

Study description	Does not apply.
Research sample	Does not apply.
Sampling strategy	Does not apply.

Data collection	Does not apply.
Timing and spatial scale	Does not apply.
Data exclusions	Does not apply.
Reproducibility	Does not apply.
Randomization	Does not apply.
Blinding	Does not apply.

Did the study involve field work? ☐ Yes ☐ No

Field work, collection and transport

Field conditions	Does not apply.
Location	Does not apply.
Access & import/export	Does not apply.
Disturbance	Does not apply.

Reporting for specific materials, systems and methods

We require information from authors about some types of materials, experimental systems and methods used in many studies. Here, indicate whether each material, system or method listed is relevant to your study. If you are not sure if a list item applies to your research, read the appropriate section before selecting a response.

Materials & experimental systems

n/a	Involved in the study	
☐	☒	Antibodies
☐	☒	Eukaryotic cell lines
☒	☐	Palaeontology and archaeology
☐	☒	Animals and other organisms
☒	☐	Clinical data
☒	☐	Dual use research of concern

Methods

n/a	Involved in the study	
☒	☐	ChIP-seq
☒	☐	Flow cytometry
☒	☐	MRI-based neuroimaging

Antibodies

Antibodies used

For immunoblotting : Anti-p21 (#sc-6246), anti-p53 (#sc-6243), anti-Cyclin A (#Sc-596), anti-GPD1 (#Sc-376219) were purchased from Santa Cruz. Anti-GK (#ab126599), anti-PCYT2 (#ab135290), anti-GPD2 (#ab188585) were purchased from Abcam. Anti-p16 (#51-16516N) was puchased from BD Pharmingen. Anti-actin (#66009-1-Ig) was purchased from Proteintech. anti-EZH2 (#5246), anti-BMI1 (#6964), anti-SUZ12 (#3737), anti-IRE1a #(3294), anti-eiF2a- p S51 (#9721), anti-Akt-p (Thr308) (#13038), anti-phospho Rb (Ser807/811) (#9308) were purchased from Cell Signaling Technology. anti- BIP (#610978) was purchased from BD Biosciences. Anti-ID1 (sc-488) was purchased from SAnta Cruz technologies. Anti-Glycerol Kinase(ET7110-96) was purchased from Huabio. Anti-a-Tubulin (T9026) was purchased form Sigma. Horseradish peroxidase-conjugated secondary antibodies used were purchased from Cell Signaling Technology (anti-Mouse, 7076S; anti-Rabbit, 7074S)
For immunofluorescence : LipidTox Red (#H34477) was purchased from Thermo Fischer Scientific. Mounting medium with DAPI (#H-1200-10) was purchased from Vectashield.

Validation

Validation statements for all the antibodies used in the study are available at the websites of the following commercial providers:
https://www.scbt.com/fr/p/p21-antibody-f-5
https://www.scbt.com/fr/p/p53-antibody-fl-393
https://www.scbt.com/p/cyclin-a-antibody-c-19
https://www.scbt.com/fr/p/gpd1-antibody-e-7

https://www.cellsignal.com/products/primary-antibodies/phospho-rb-ser807-811-antibody/9308
https://www.abcam.com/en-sa/products/primary-antibodies/glycerol-kinase-antibody-epr6567-ab126599
https://www.abcam.com/products/primary-antibodies/pcyt2-antibody-ab135290.html
https://www.abcam.com/products/primary-antibodies/gpd2-antibody-epr14259-ab188585.html
https://www.bdbiosciences.com/en-us/products/reagents/microscopy-imaging-reagents/immunohistochemistry-reagents/purified-mouse-anti-human-p16-with-control.551154
https://www.ptglab.com/fr/products/Pan-Actin-Antibody-66009-1-Ig.html
https://www.cellsignal.com/products/primary-antibodies/ezh2-d2c9-xp-rabbit-mab/5246
https://www.cellsignal.com/products/primary-antibodies/bmi1-d20b7-xp-rabbit-mab/6964
https://www.cellsignal.com/products/primary-antibodies/suz12-d39f6-xp-rabbit-mab/3737
https://www.cellsignal.com/products/primary-antibodies/ire1a-14c10-rabbit-mab/3294
https://www.cellsignal.com/products/primary-antibodies/phospho-eif2a-ser51-antibody/9721
https://www.cellsignal.com/products/primary-antibodies/phospho-akt-thr308-d25e6-xp-rabbit-mab/13038
https://www.cellsignal.com/products/primary-antibodies/phospho-rb-ser807-811-antibody/9308
https://www.bdbiosciences.com/en-us/products/reagents/western-blotting-and-molecular-reagents/western-blot-reagents/purified-mouse-anti-bip-grp78.610978
https://www.citeab.com/antibodies/800109-sc-488-id1-antibody-c-20
https://www.huabio.com/products/glycerol-kinase-antibody-clone-je55-12-recombinant-monoclonal-et7110-96

Eukaryotic cell lines

Policy information about cell lines and Sex and Gender in Research

Cell line source(s)	WI-38 fibroblasts cell line, purchased from ATCC. Primary human myoblasts (SkMC) were isolated from a skeletal muscle biopsy of a healthy donor (PromoCell #C-12530, Lot 414Z025.11)
Authentication	WI38 and myoblasts cell lines were obtained from original source and were not further authenticated
Mycoplasma contamination	WI38 cells were monthly tested for potential contaminations with mycoplasma using commercial PCR Mycoplasma Detection Kit (ABM, #G-238). All tests were negative.
Commonly misidentified lines (See ICLAC register)	No commonly misidentified cell line was used in the study.

Palaeontology and Archaeology

Specimen provenance	Does not apply.
Specimen deposition	Does not apply.
Dating methods	Does not apply.

☐ Tick this box to confirm that the raw and calibrated dates are available in the paper or in Supplementary Information.

Ethics oversight	Does not apply.

Note that full information on the approval of the study protocol must also be provided in the manuscript.

Animals and other research organisms

Policy information about studies involving animals; ARRIVE guidelines recommended for reporting animal research, and Sex and Gender in Research

Laboratory animals	PIK3CAAdipo-CreER mice and LSL- KrasG12DPtf1a-Cre transgenic mice were used in the study. LSL- KrasG12DPtf1a-Cre mice were sacrificed at 2 months, while PIK3CAAdipo-CreER mice were sacrificed at 12 weeks. Animals were fed ad libitum and housed at a constant ambient temperature in a 12-hour light cycle. PIK3CAAdipo-CreER mice were fed with regular chow food (2018 Teklad global 18% protein rodent diets, 3.1 kcal/g; Envigo).
Wild animals	Does not apply.
Reporting on sex	Only male LSL- KrasG12DPtf1a-Cre and control mice were used in the study. 11 males and 7 females PIK3CAAdipo-CreER mice were used in this study.
Field-collected samples	Does not apply.
Ethics oversight	Prefecture de Police, Paris, France; authorization number 75–1313 and APAFIS #34979 approved the experiments involving the mice.

Note that full information on the approval of the study protocol must also be provided in the manuscript.

Clinical data

Policy information about clinical studies

All manuscripts should comply with the ICMJE guidelines for publication of clinical research and a completed CONSORT checklist must be included with all submissions.

Clinical trial registration	Does not apply.
Study protocol	Does not apply.
Data collection	Does not apply.
Outcomes	Does not apply.

Dual use research of concern

Policy information about dual use research of concern

Hazards

Could the accidental, deliberate or reckless misuse of agents or technologies generated in the work, or the application of information presented in the manuscript, pose a threat to:

No	Yes	
☒	☐	Public health
☒	☐	National security
☒	☐	Crops and/or livestock
☒	☐	Ecosystems
☒	☐	Any other significant area

Experiments of concern

Does the work involve any of these experiments of concern:

No	Yes	
☒	☐	Demonstrate how to render a vaccine ineffective
☒	☐	Confer resistance to therapeutically useful antibiotics or antiviral agents
☒	☐	Enhance the virulence of a pathogen or render a nonpathogen virulent
☒	☐	Increase transmissibility of a pathogen
☒	☐	Alter the host range of a pathogen
☒	☐	Enable evasion of diagnostic/detection modalities
☒	☐	Enable the weaponization of a biological agent or toxin
☒	☐	Any other potentially harmful combination of experiments and agents

ChIP-seq

Data deposition

☐ Confirm that both raw and final processed data have been deposited in a public database such as GEO.

☐ Confirm that you have deposited or provided access to graph files (e.g. BED files) for the called peaks.

Data access links<br>*May remain private before publication.*	Does not apply.
Files in database submission	Does not apply.
Genome browser session<br>(e.g. UCSC)	Does not apply.

Methodology

Replicates	Does not apply.
Sequencing depth	Does not apply.

Antibodies	Does not apply.
Peak calling parameters	Does not apply.
Data quality	Does not apply.
Software	Does not apply.

Flow Cytometry

Plots

Confirm that:

- [ ] The axis labels state the marker and fluorochrome used (e.g. CD4-FITC).
- [ ] The axis scales are clearly visible. Include numbers along axes only for bottom left plot of group (a 'group' is an analysis of identical markers).
- [ ] All plots are contour plots with outliers or pseudocolor plots.
- [ ] A numerical value for number of cells or percentage (with statistics) is provided.

Methodology

Sample preparation	Does not apply.
Instrument	Does not apply.
Software	Does not apply.
Cell population abundance	Does not apply.
Gating strategy	Does not apply.

- [ ] Tick this box to confirm that a figure exemplifying the gating strategy is provided in the Supplementary Information.

Magnetic resonance imaging

Experimental design

Design type	Does not apply.
Design specifications	Does not apply.
Behavioral performance measures	Does not apply.

Acquisition

Imaging type(s)	Does not apply.
Field strength	Does not apply.
Sequence & imaging parameters	Does not apply.
Area of acquisition	Does not apply.

Diffusion MRI [ ] Used [x] Not used

Preprocessing

Preprocessing software	Does not apply.
Normalization	Does not apply.
Normalization template	Does not apply.
Noise and artifact removal	Does not apply.
Volume censoring	Does not apply.

Statistical modeling & inference

Model type and settings	Does not apply.
Effect(s) tested	Does not apply.

Specify type of analysis: ☐ Whole brain ☐ ROI-based ☐ Both

Statistic type for inference (See Eklund et al. 2016)	Does not apply.
Correction	Does not apply.

Models & analysis

n/a	Involved in the study
☒	☐ Functional and/or effective connectivity
☒	☐ Graph analysis
☒	☐ Multivariate modeling or predictive analysis

Functional and/or effective connectivity	Does not apply.
Graph analysis	Does not apply.
Multivariate modeling and predictive analysis	Does not apply.
