# Supplementary figures and images for: A homoeostatic switch causing glycerol-3-phosphate and phosphoethanolamine accumulation triggers senescence by rewiring lipid metabolism
Source: Nat Metab. 2024 Feb 19;6(2):323–42. doi: 10.1038/s42255-023-00972-y (PMC10896726; doi:10.1038/s42255-023-00972-y)

Fig.4F

RAS-OIS

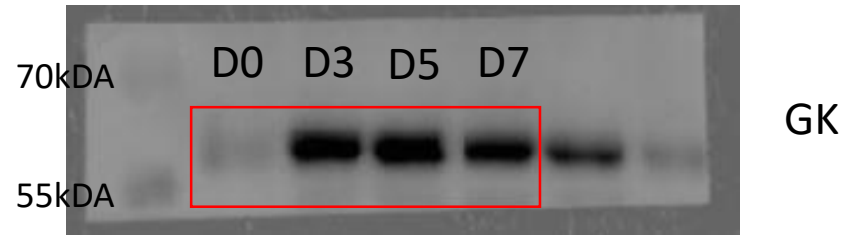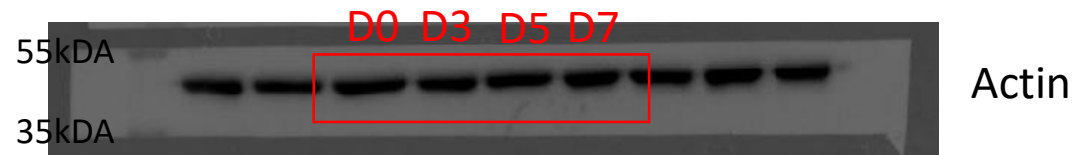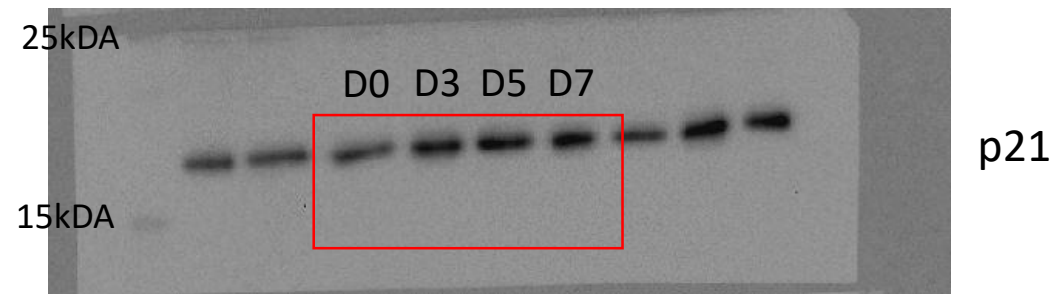

DDIS

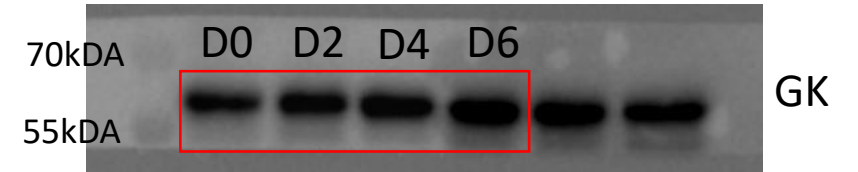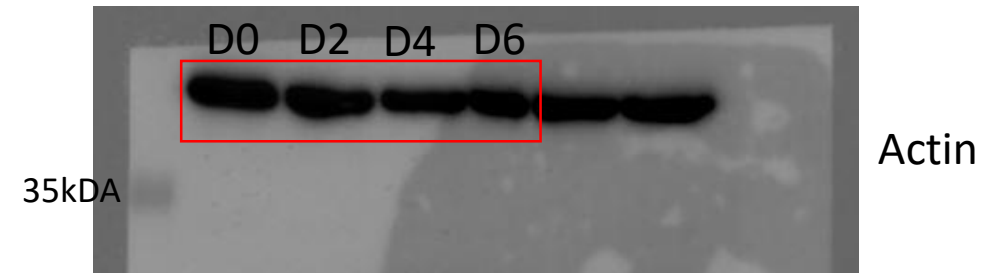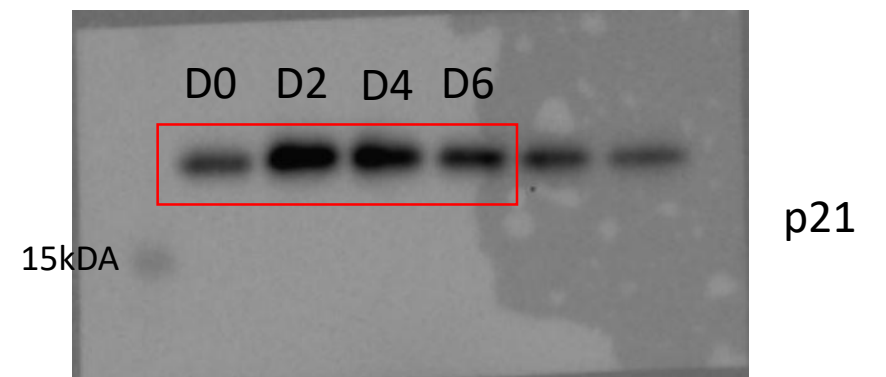

Fig.4H

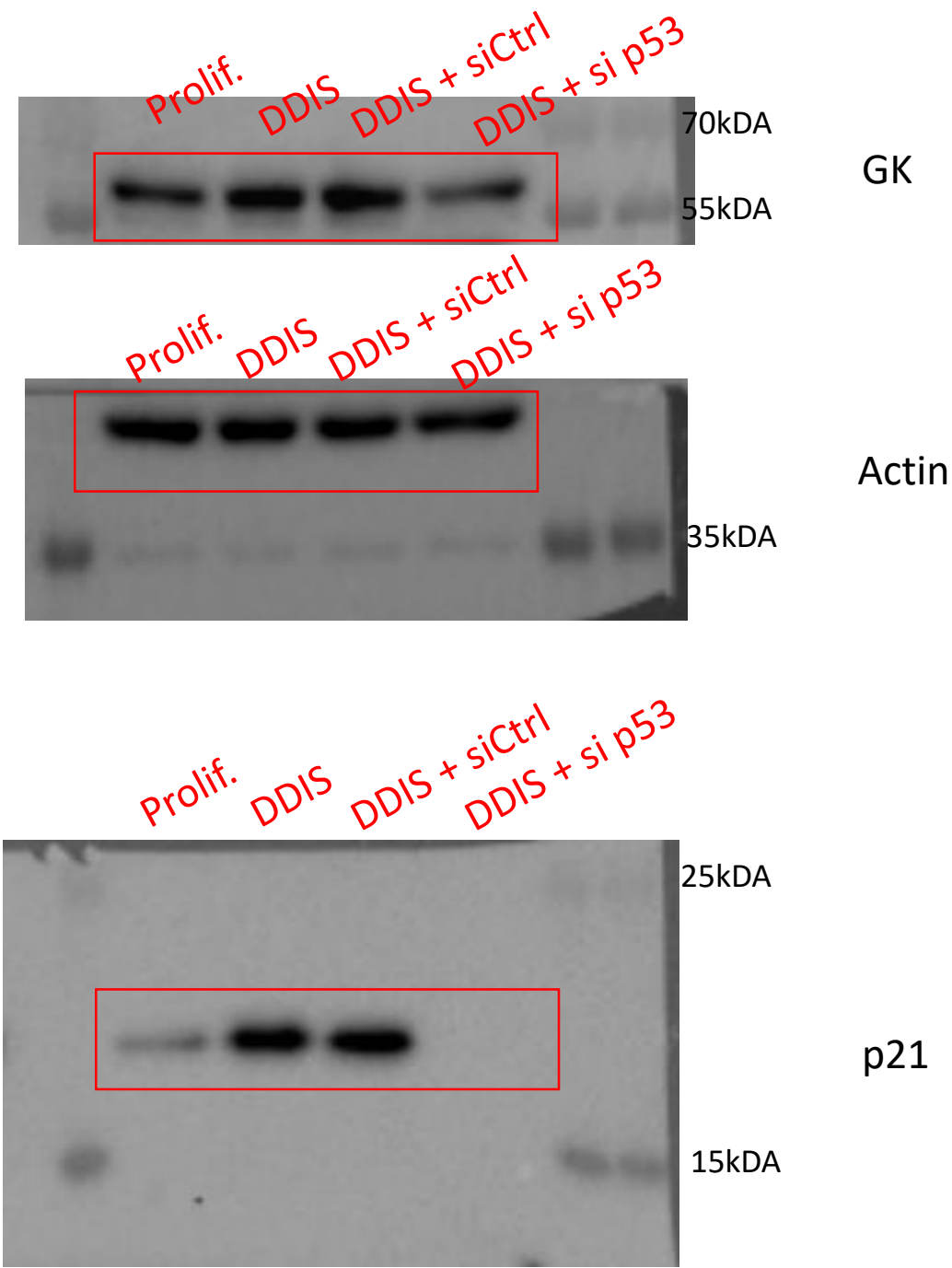

Supplement: Supplementary file 20 — Unprocessed western blots. [file 42255_2023_972_MOESM20_ESM.pdf]

Fig.6C

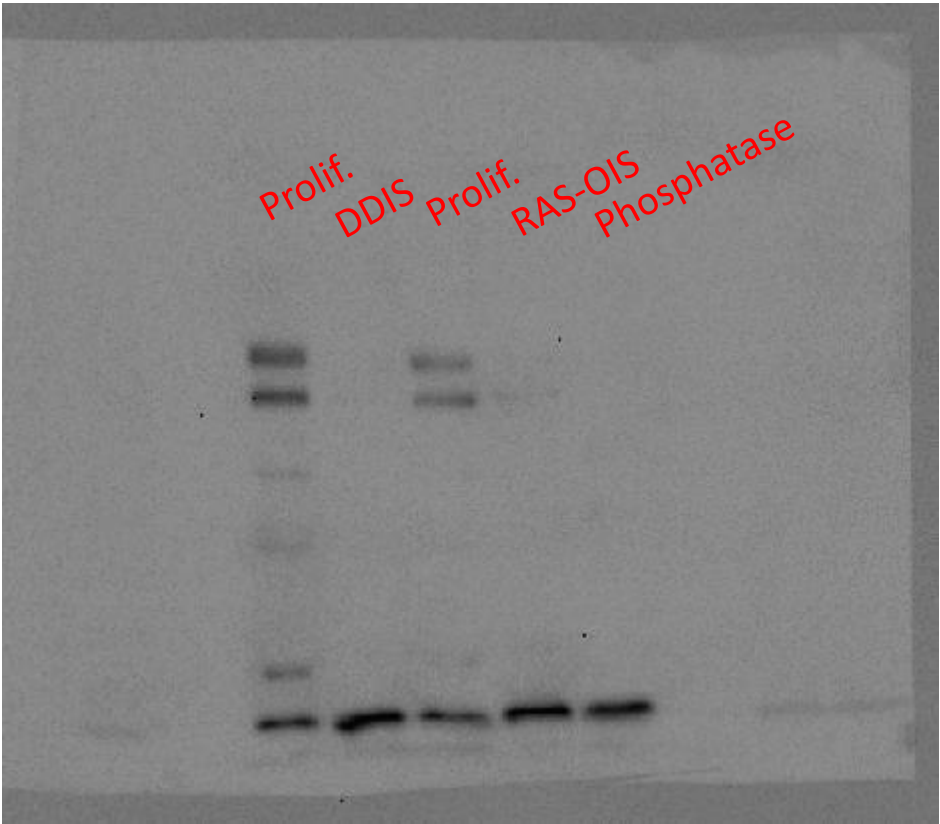

PCYT2  
Phostag

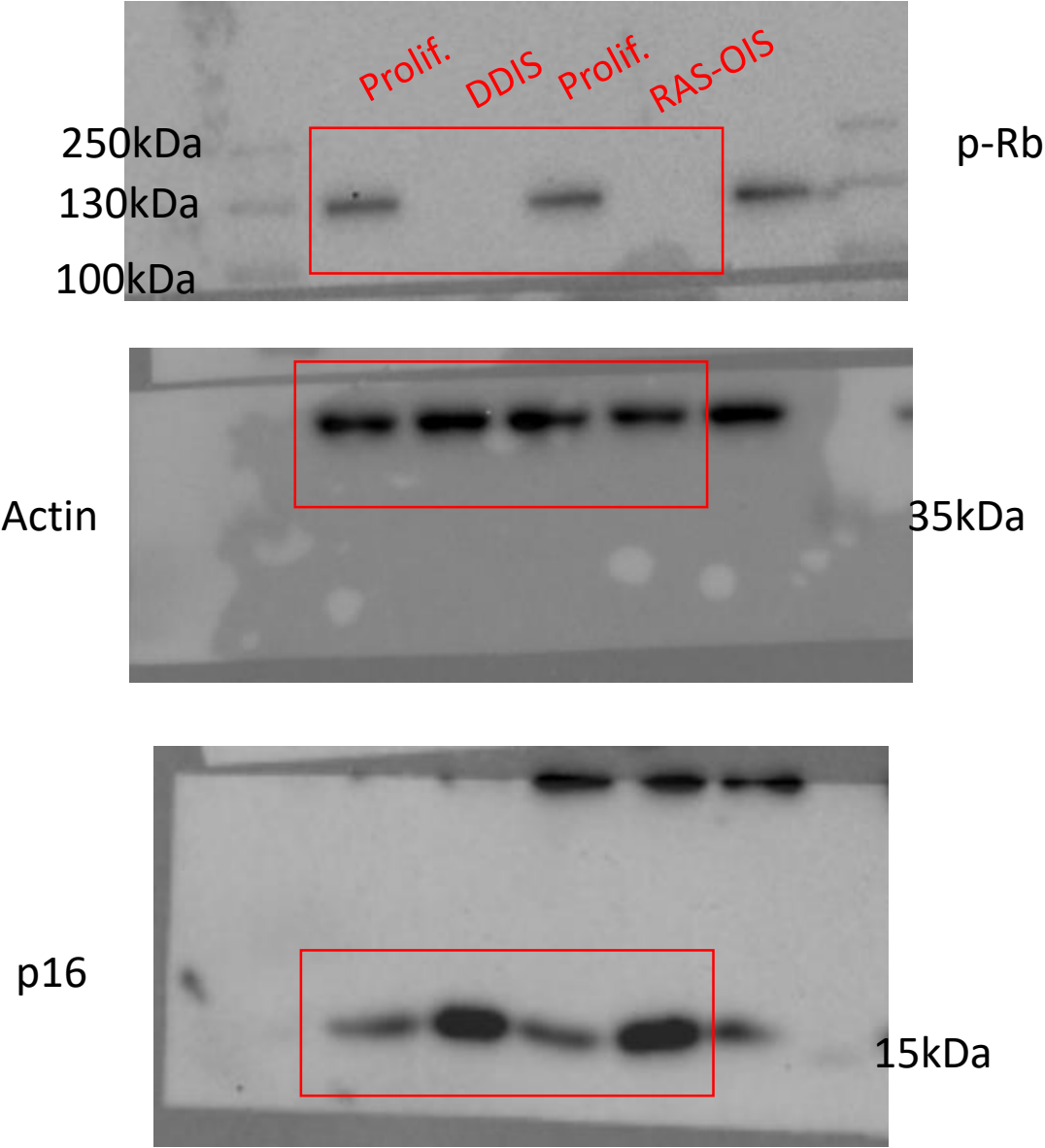

Fig.6D

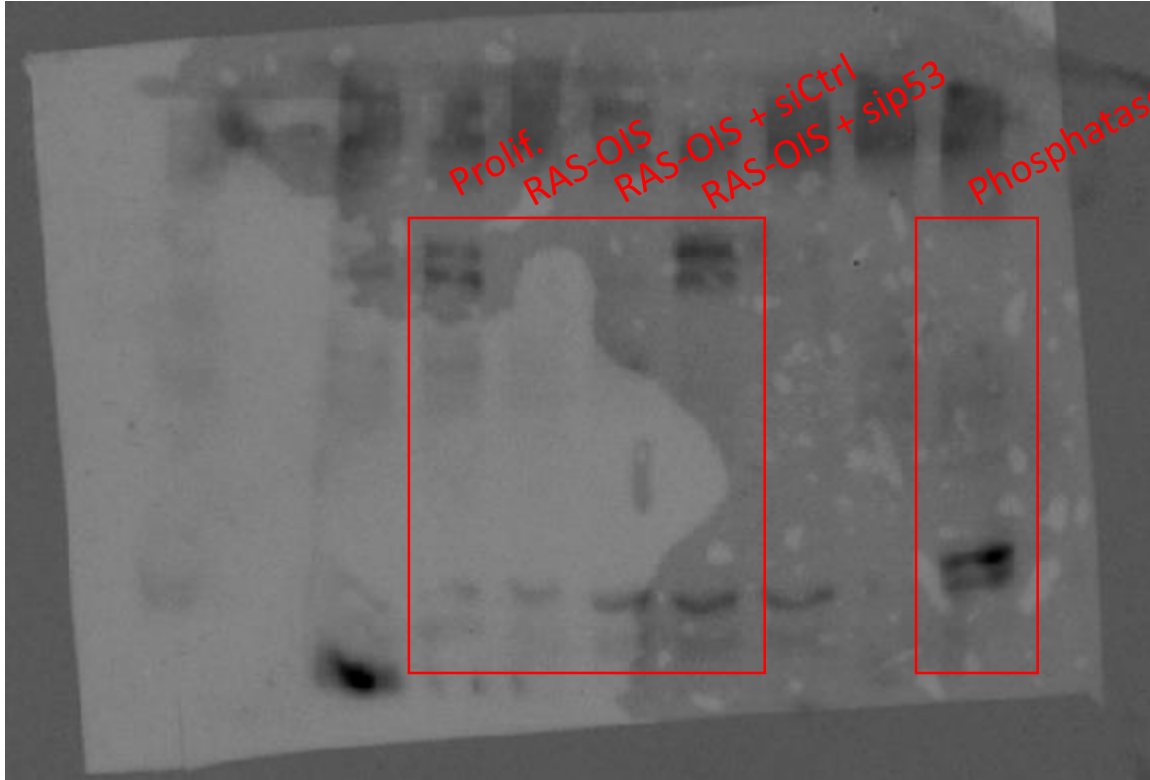

PCYT2  
Phostag

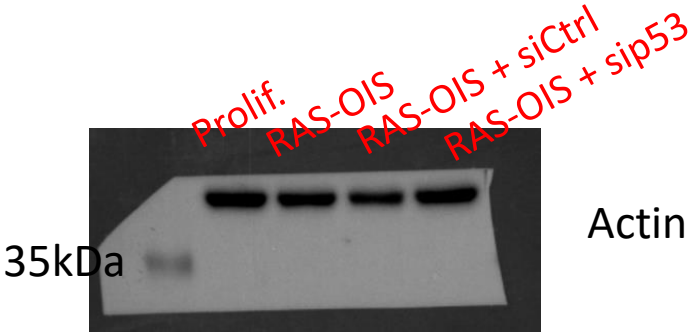

35kDa

Actin

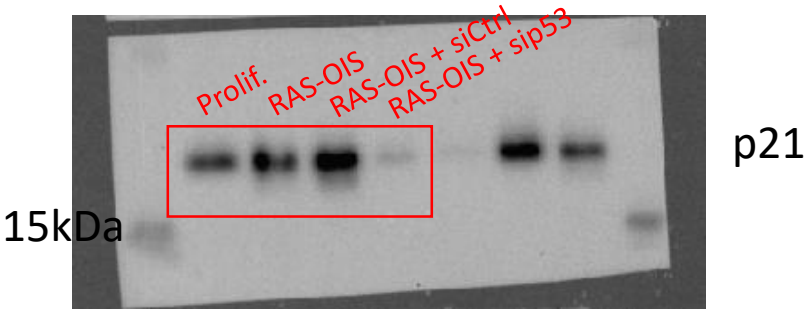

15kDa

p21

Fig.6F

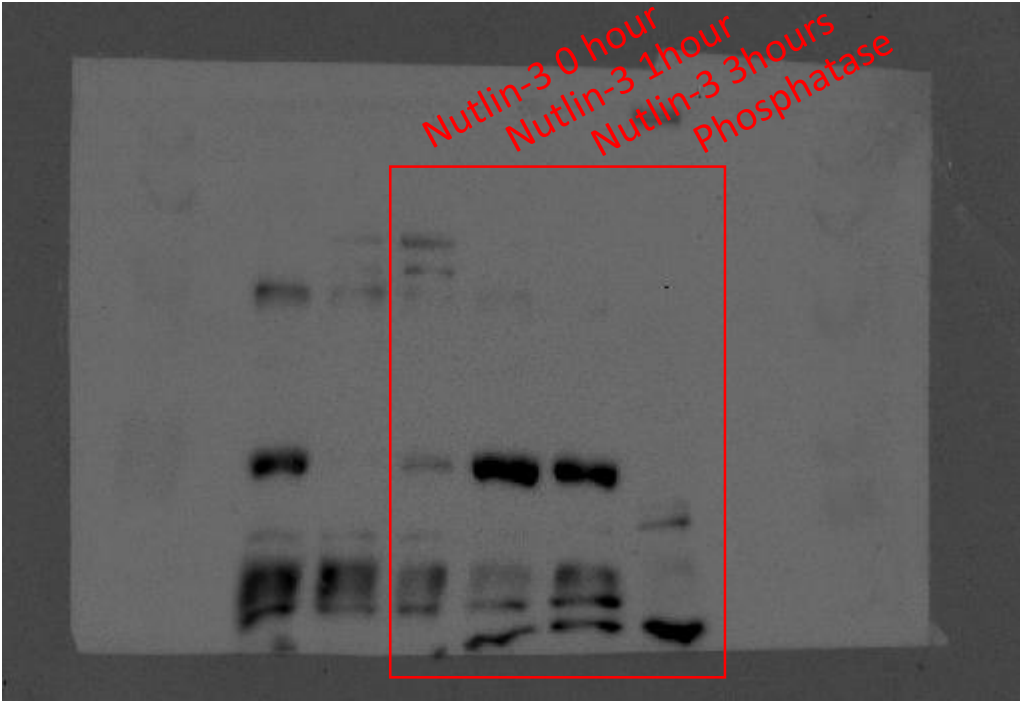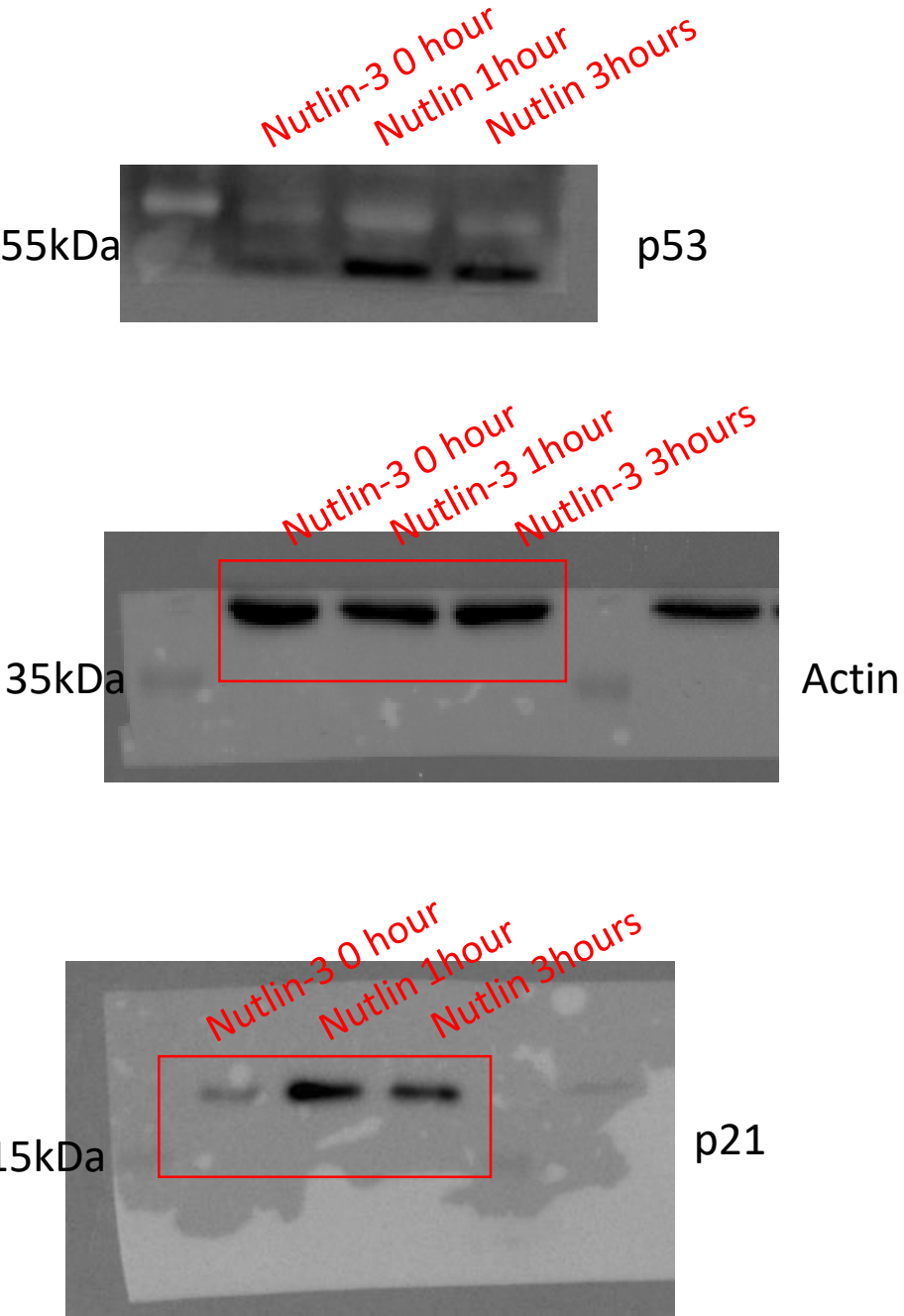

Fig.6H

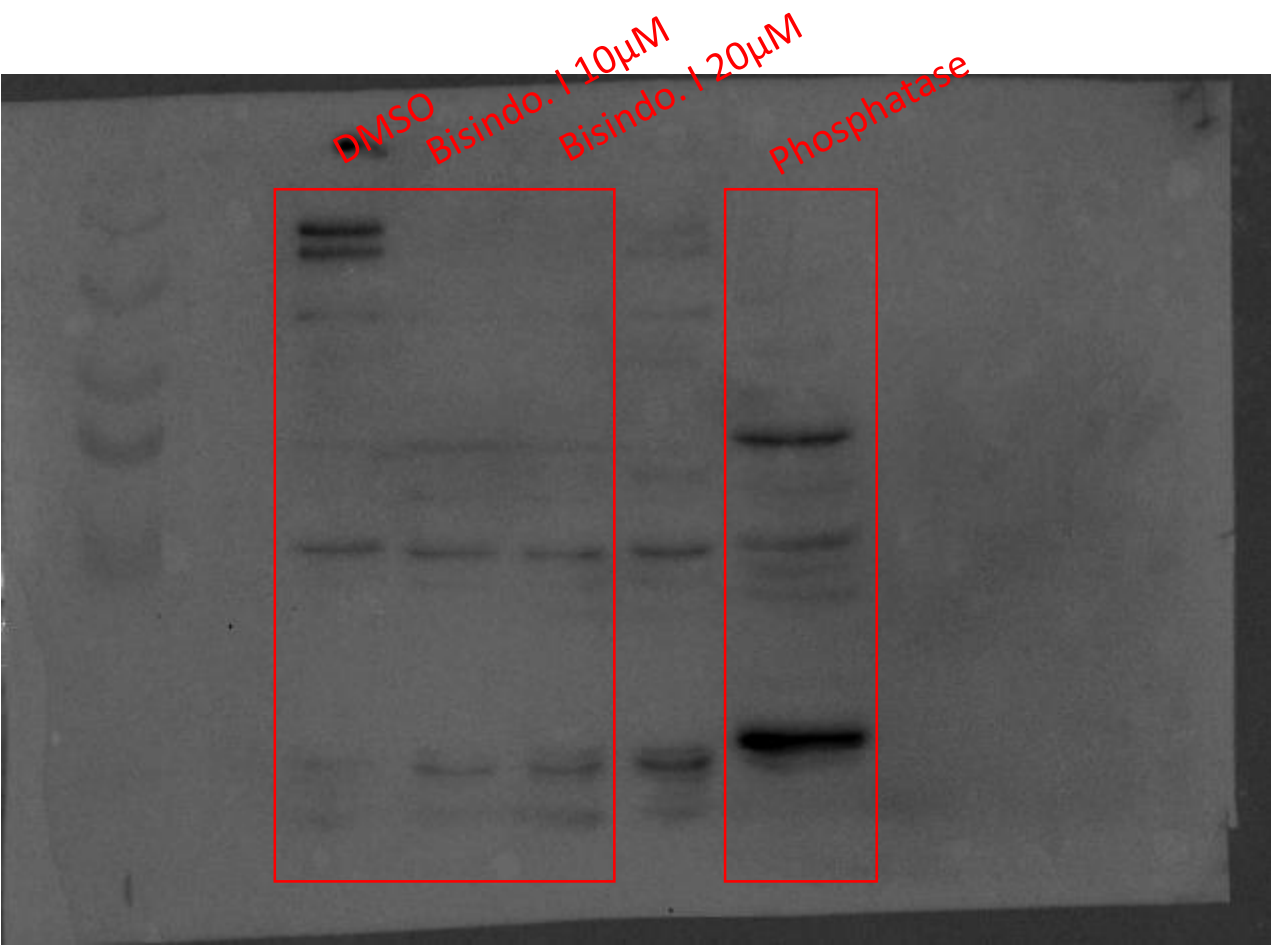

PCYT2  
Phostag

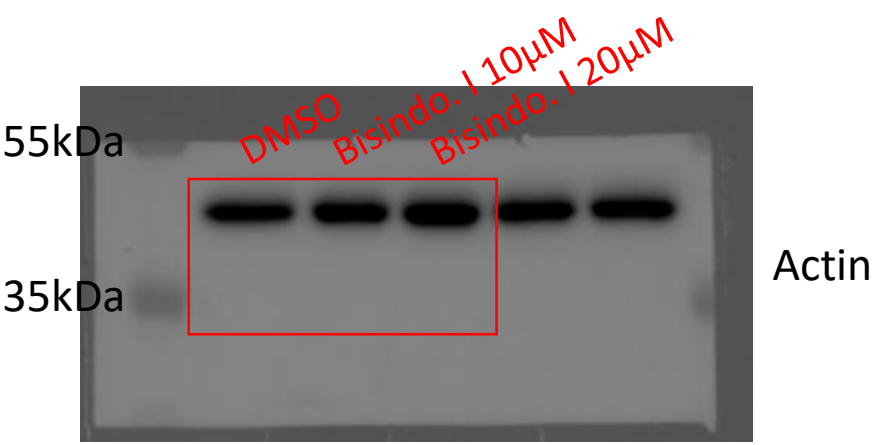

Supplement: Supplementary file 23 — Unprocessed western blots. [file 42255_2023_972_MOESM23_ESM.pdf]

Fig.8D

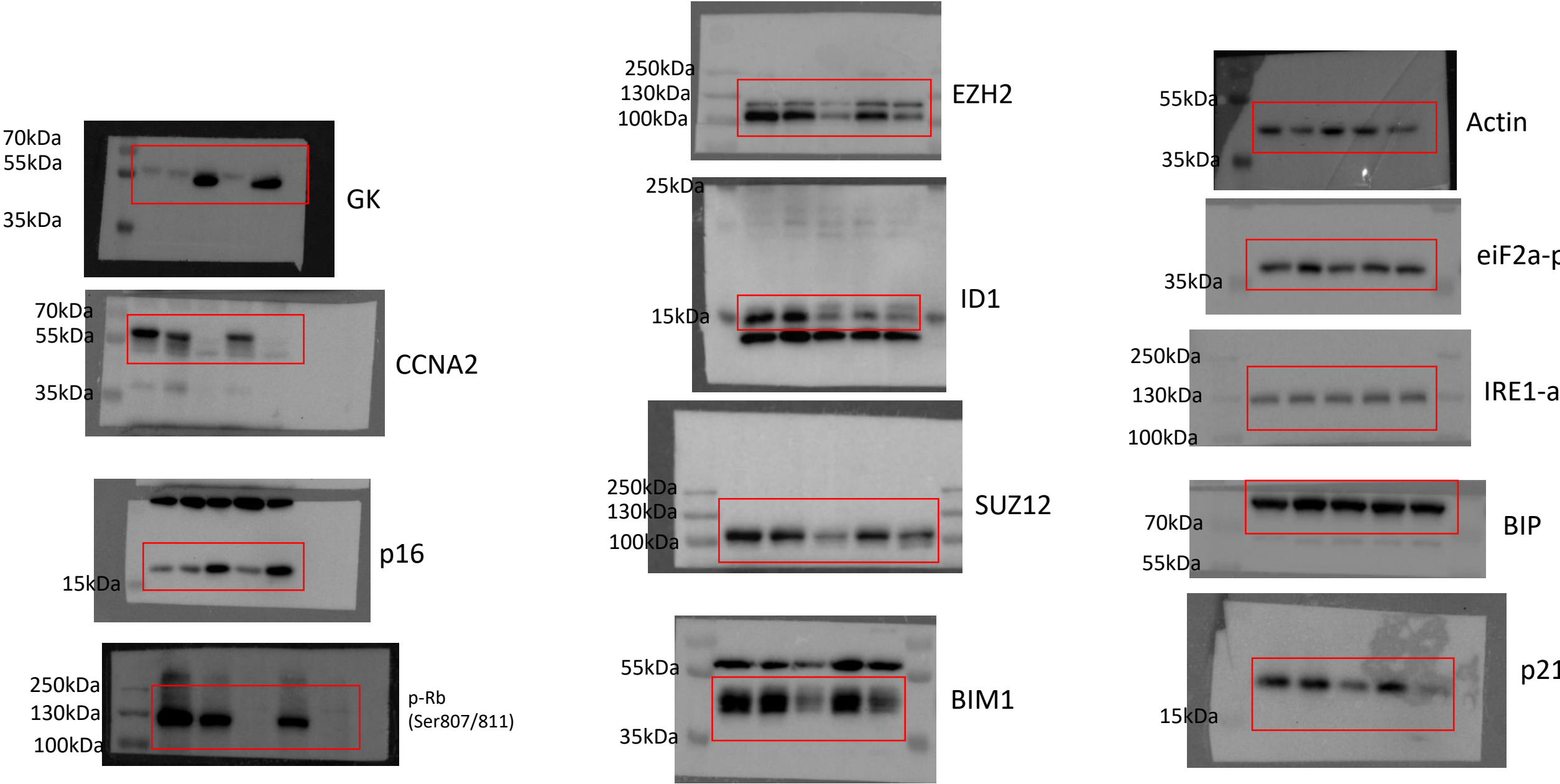

Fig.8E

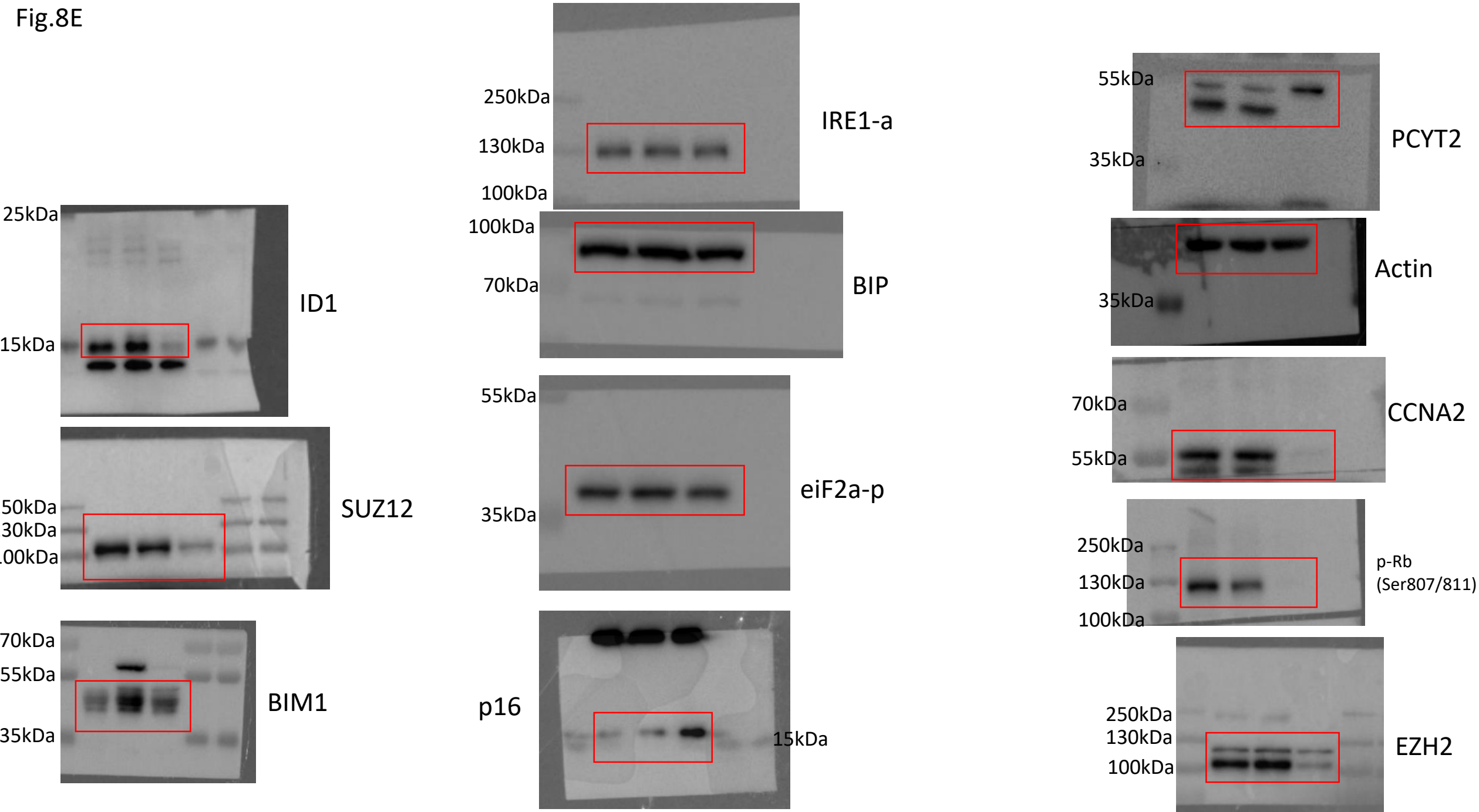

Supplement: Supplementary file 26 — Unprocessed western blots. [file 42255_2023_972_MOESM26_ESM.pdf]

ED Fig.6C

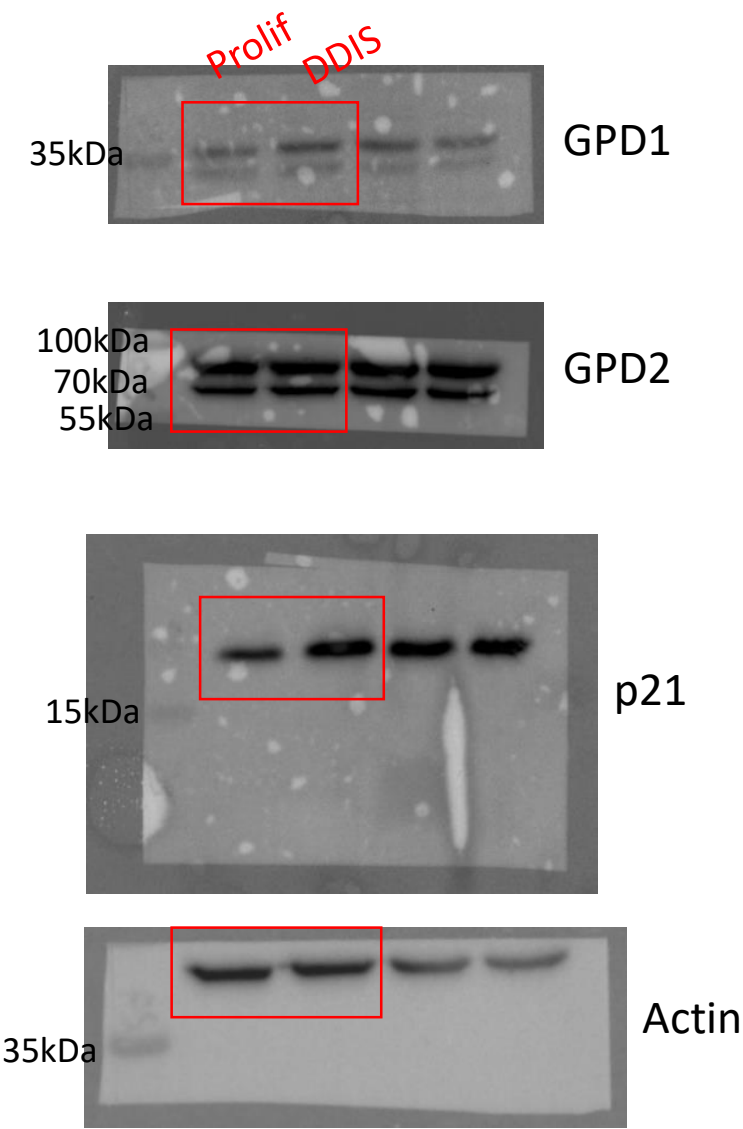

ED Fig.6E

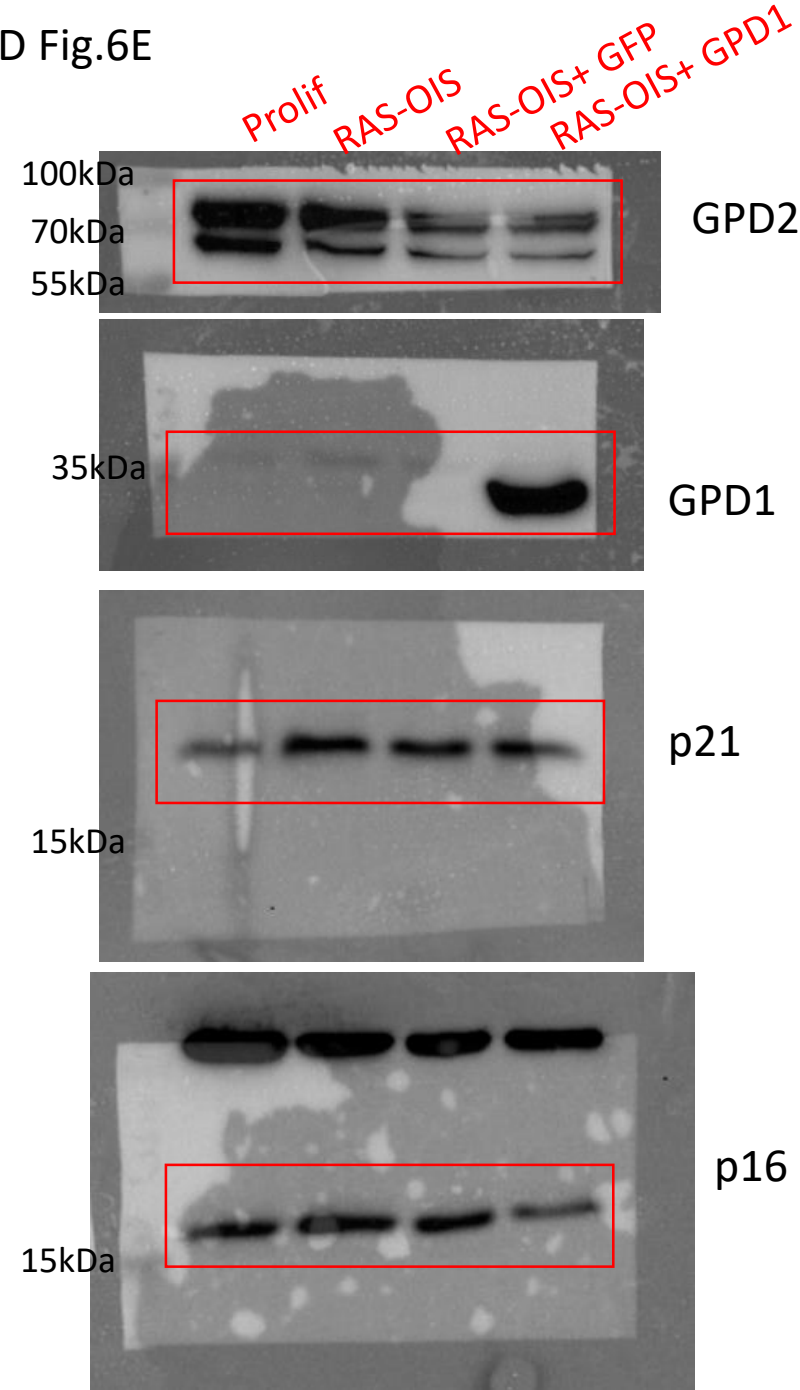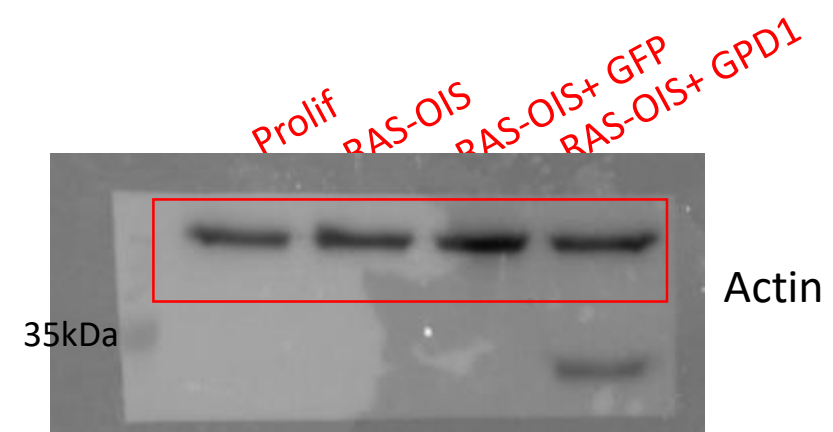

Supplement: Supplementary file 30 — Unprocessed western blots. [file 42255_2023_972_MOESM30_ESM.pdf]

ED Fig.7A

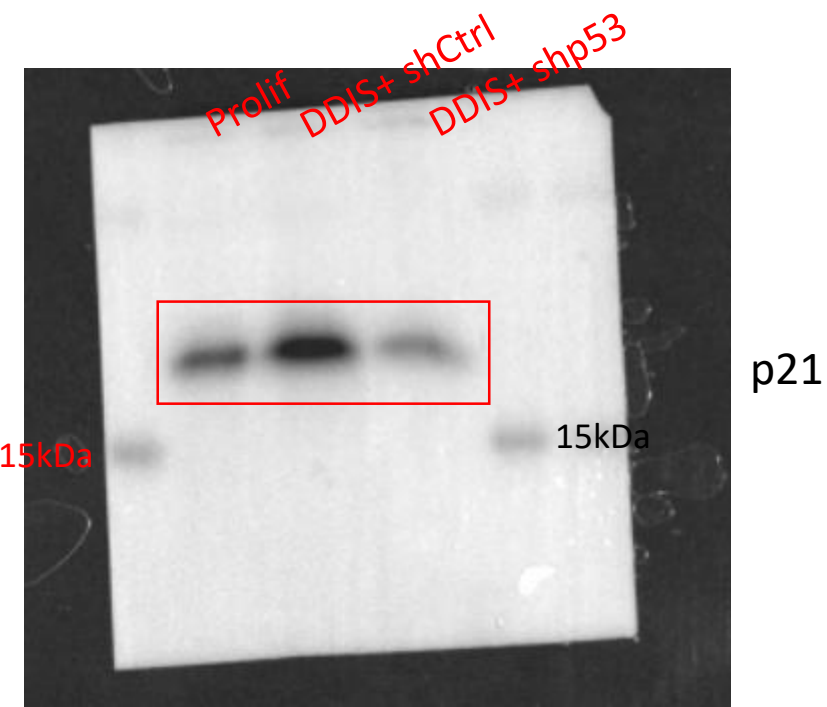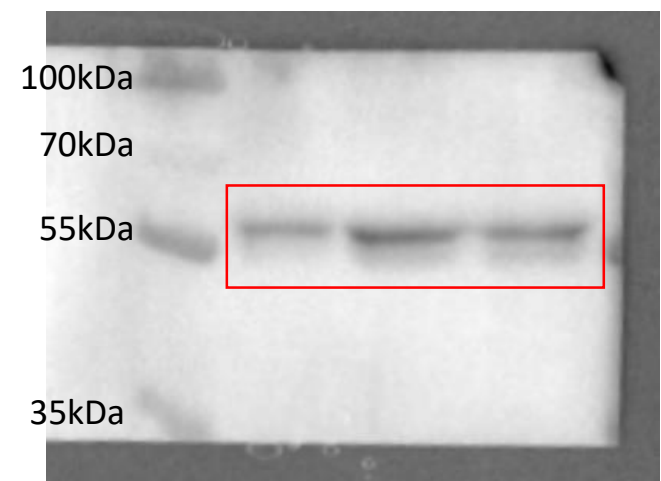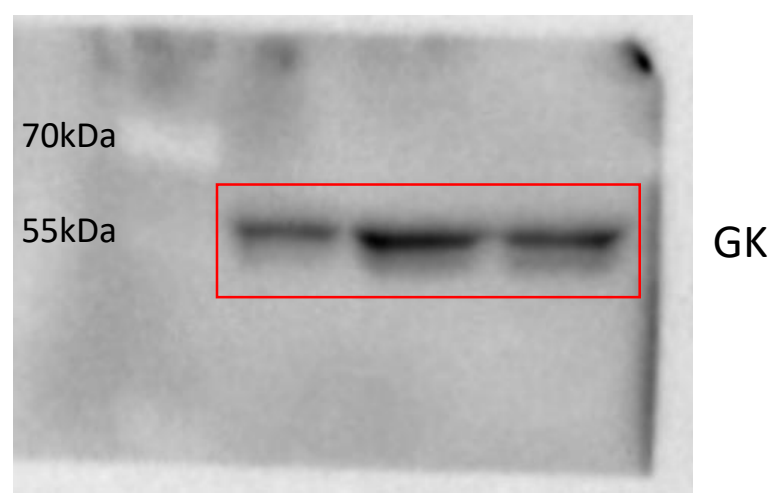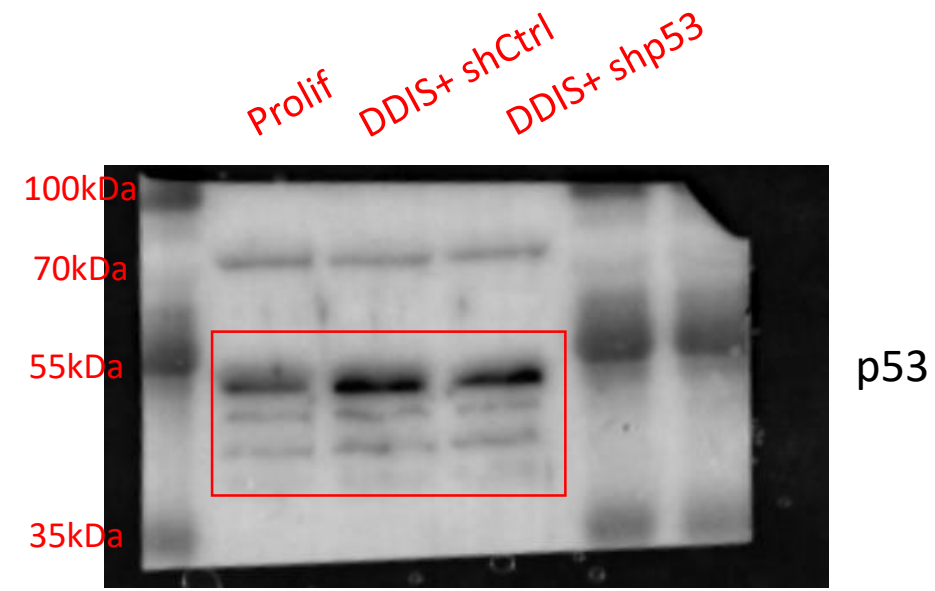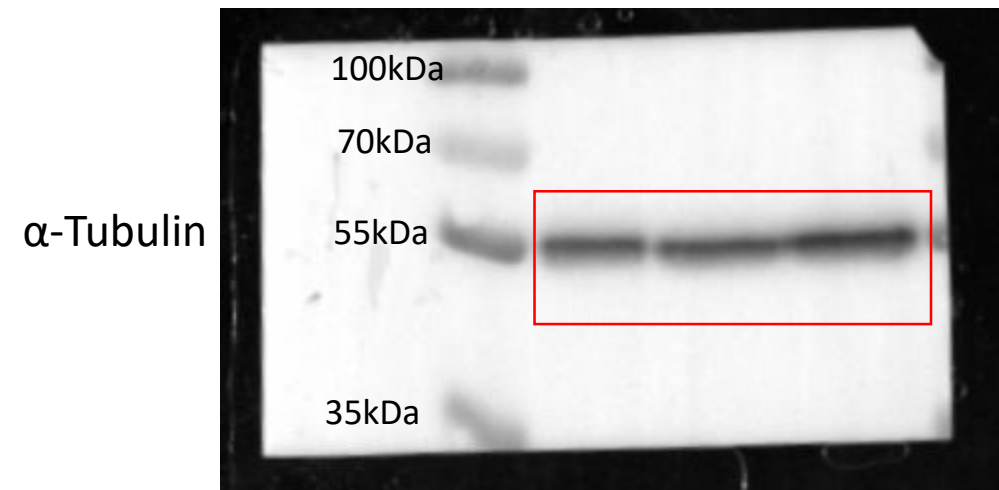

ED Fig.7B

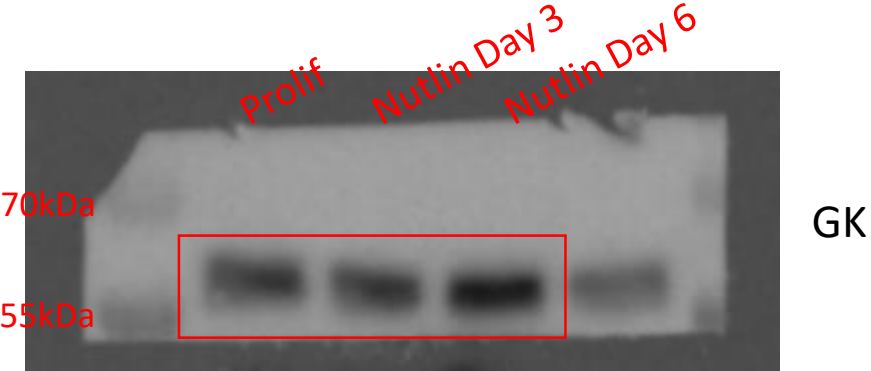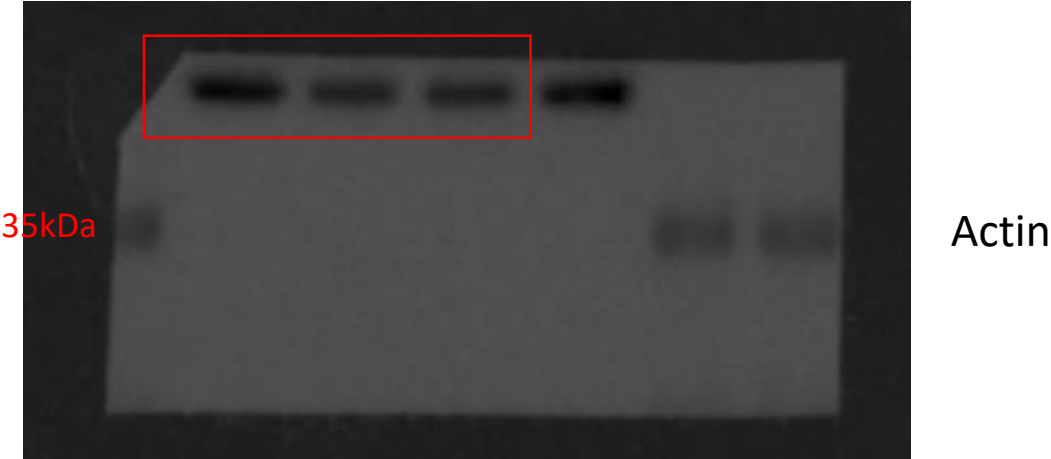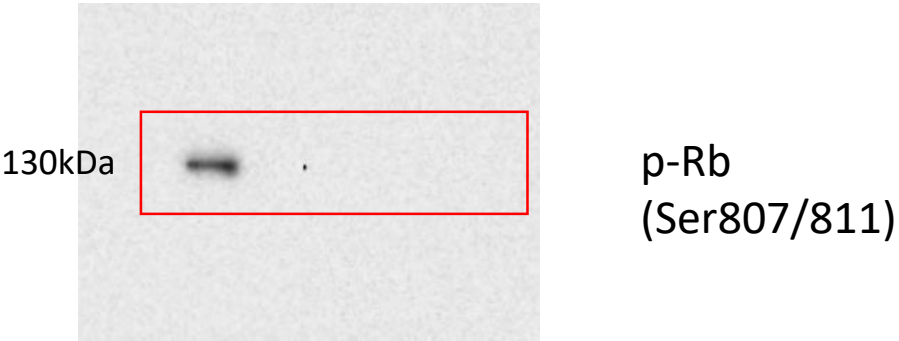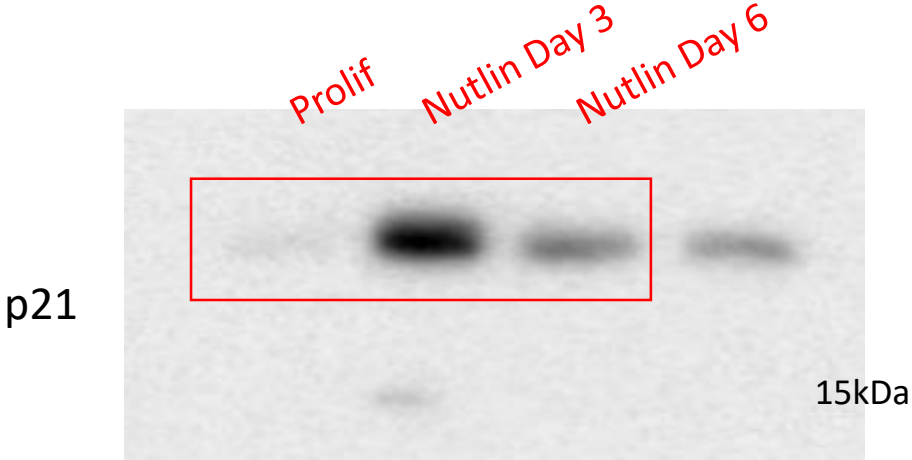

Supplement: Supplementary file 32 — Unprocessed western blots. [file 42255_2023_972_MOESM32_ESM.pdf]

ED Fig.8F

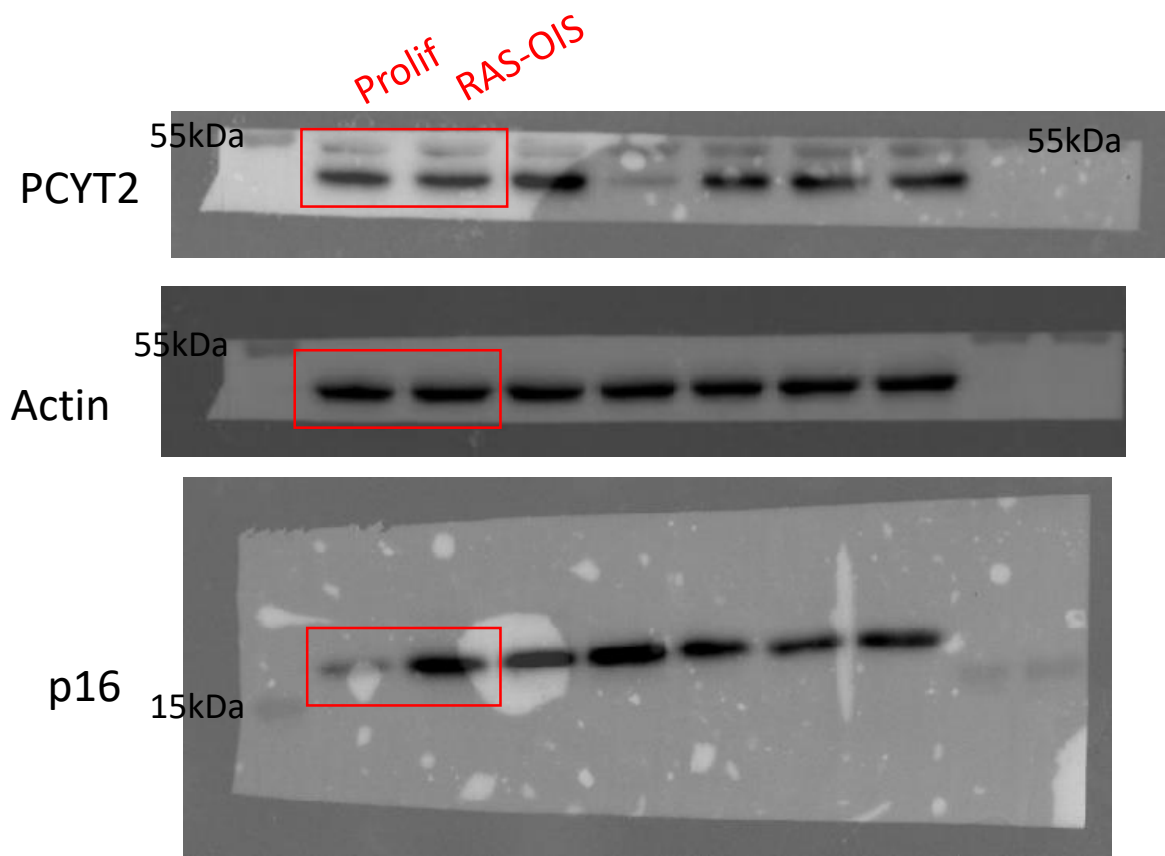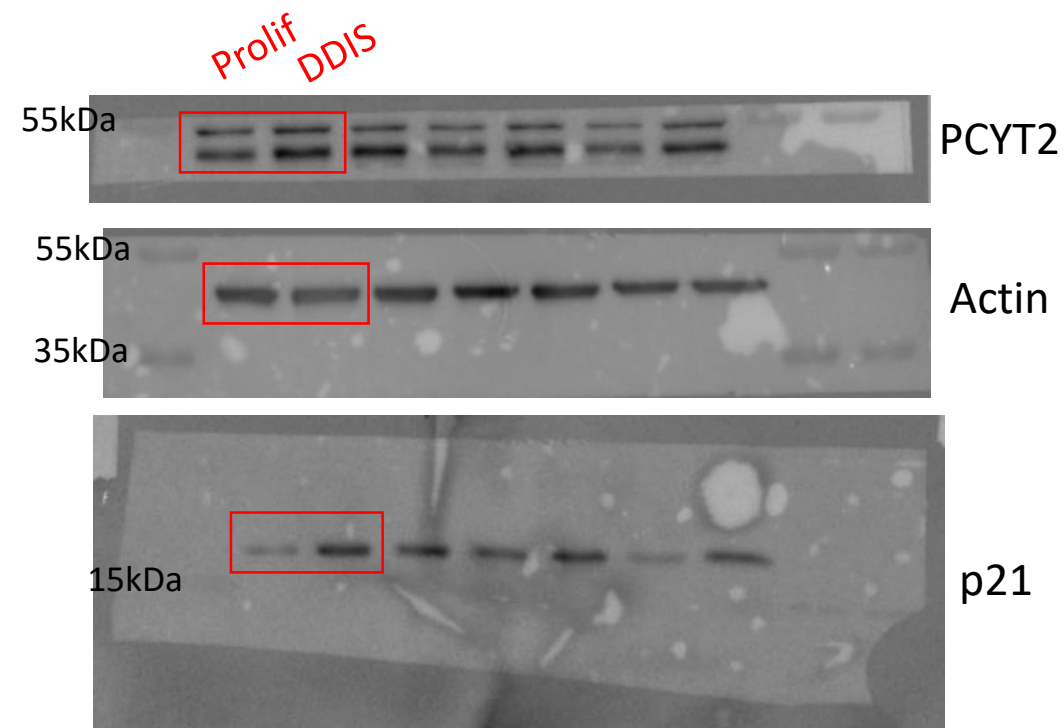

Supplement: Supplementary file 34 — Unprocessed western blots. [file 42255_2023_972_MOESM34_ESM.pdf]

ED Fig.9D

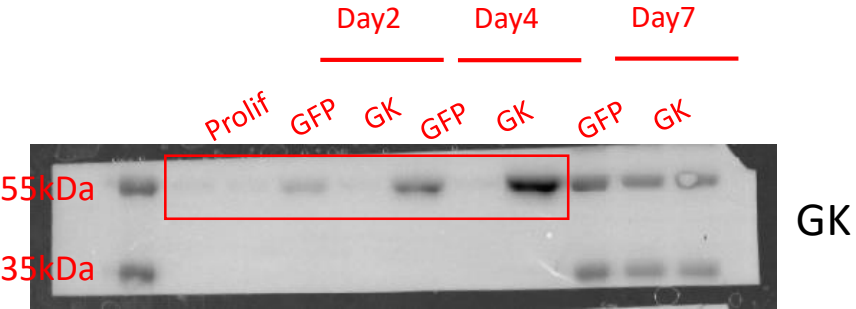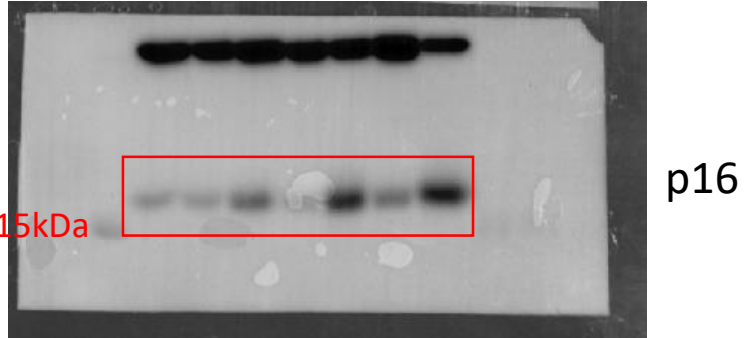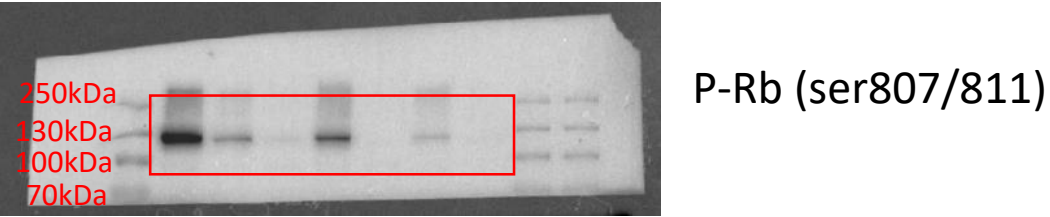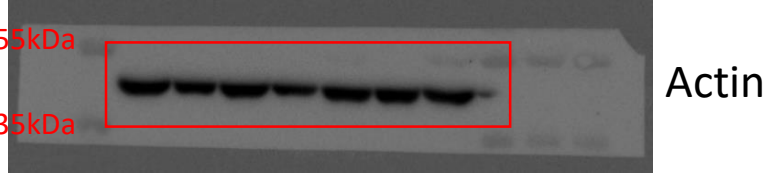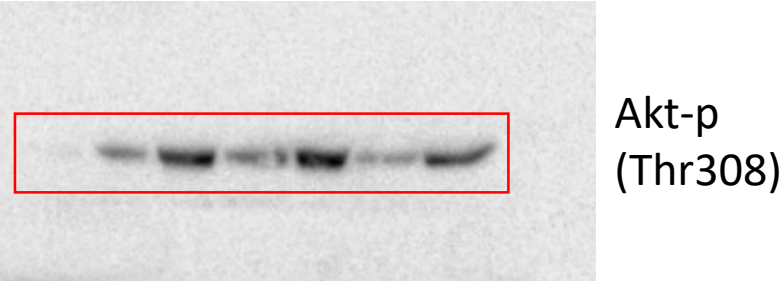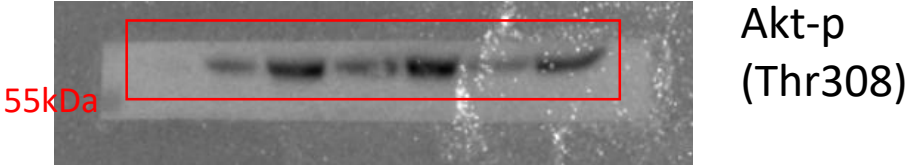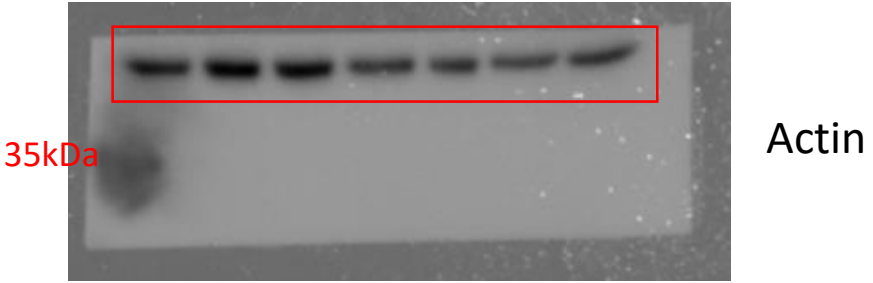

Supplement: Supplementary file 36 — Unprocessed western blots. [file 42255_2023_972_MOESM36_ESM.pdf]

ED Fig.10B

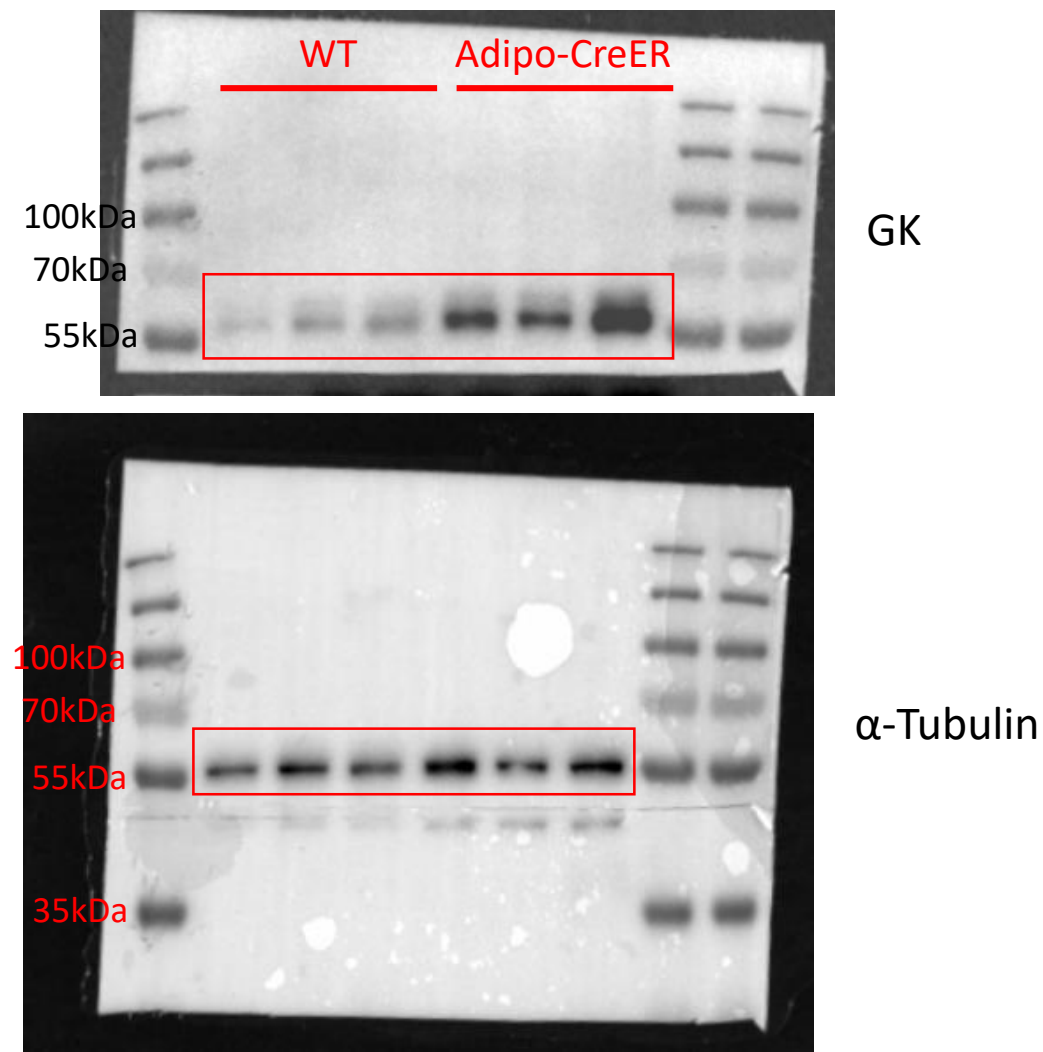

Supplement: Supplementary file 38 — Unprocessed western blots. [file 42255_2023_972_MOESM38_ESM.pdf]
